# Reporting of Telehealth Implementation in Cystic Fibrosis: Scoping Review Using a Novel Theory-Based Evaluation Lens

**DOI:** 10.2196/86194

**Published:** 2026-05-22

**Authors:** Tamara Vagg, Robyn Doherty, Sarath C Ranganathan, Barry J Plant

**Affiliations:** 1 Cork Centre for Cystic Fibrosis Cork University Hospital University College Cork Cork Ireland; 2 The HRB funded Clinical Research Facility University College Cork Cork Ireland; 3 Department of Medicine University College Cork Cork Ireland; 4 Cystic Fibrosis Registry of Ireland University College Dublin Cystic Fibrosis Registry of Ireland Dublin Ireland; 5 Respiratory and Sleep Medicine Department Royal Children's Hospital Melbourne Australia; 6 Respiratory Diseases Research Murdoch Children's Research Institute Melbourne Australia; 7 Department of Paediatrics The University of Melbourne Melbourne Australia

**Keywords:** Guidelines and Checklist for the Reporting on Digital Health Implementations, iCHECK-DH, Template for Intervention Description and Replication for Telehealth, TIDiER-telehealth, scoping review, telehealth, cystic fibrosis, implementation

## Abstract

**Background:**

Many inductive reviews exploring telehealth and its application in health care have identified missing or inconsistently reported implementation data, calling for a standardized approach to telehealth research.

**Objective:**

Using cystic fibrosis (CF) as a case exemplar, this study evaluated the adherence of telehealth research to standardized reporting frameworks through a theory-based evaluation lens to assess implementation reporting quality and identify knowledge gaps and strengths across the literature.

**Methods:**

We conducted an updated systematic review of the PubMed, Scopus, and Web of Science databases using a novel deductive approach to identify relevant scientific papers available in English and focusing on the delivery of telehealth interventions to CF populations as part of or alongside routine CF care. Two relevant reporting checklists were identified in the Equator Network database (Guidelines and Checklist for the Reporting on Digital Health Implementations [iCHECK-DH] and Template for Intervention Description and Replication for Telehealth [TIDiER-telehealth]) to extract data from the papers. Each checklist category was described as being “fully reported” (score=2), “partially reported” (score=1), and “did not report” (score=0) for each paper. An overall score was calculated for adherence to the checklists.

**Results:**

In total, 98 studies published between 2006 and May 2025 were included in this review, with the majority appearing during the COVID-19 pandemic (2021-2022). Most studies were conducted in a single country, predominantly the United States, Australia, and the United Kingdom, and were published in medical journals. Telehealth was variably described, with video call–based models in combination with remote monitoring being most common. The median score was 22/40 (range 11-29, 55.0% adherent) for iCHECK and 15/24 (range 6-23, 62.5% adherent) for TIDiER, demonstrating moderate overall reporting quality. For iCHECK, ≥50% of studies fully reported 6/20 categories, partially reported 9/20 categories, and did not report 3/20 categories. For TIDiER, ≥50% of studies fully reported 4/12 categories, partially reported 6/12 categories, and did not report 1/12 categories, indicating persistent gaps in intervention description despite improved partial reporting.

**Conclusions:**

Key areas, such as justification for telehealth, target populations, and outcomes, are well documented, offering valuable insights into the rationale for and outcomes associated with telehealth. However, implementation processes remain underreported, partly due to the more recent adoption of frameworks like iCHECK and TIDiER. The clinical implications of the current evidence limit the implementation of telehealth in terms of the ability to assess feasibility and readiness for adoption; understand financial implications and plan sustainably; ensure patient safety, data protection, and equity; interpret outcome data in context; and share, replicate, or scale evidence-based models of care. Strengthening the commitment to standardized telehealth reporting will ultimately support clinical decision-making and improve the effective and equitable integration of telehealth into care.

## Introduction

### Background

Telehealth has existed for several decades but experienced a large increase in adoption during the COVID-19 pandemic [1]. Over time, telehealth’s definition has changed to match current technology capabilities and infrastructure contexts. As a result, there is no universally accepted definition of telehealth, and the term is often used interchangeably with other terms, such as telemedicine, eHealth, and virtual monitoring [2,3]. For this review, telehealth refers to interventions/care delivered synchronously by a health professional to a patient over the phone or via video [4]. Much research in this field often aims to synthesize and produce evidence to support the adoption of telehealth in different health contexts, including chronic disease care and management across different disease/condition populations [5-7]. However, similar to the difficulty with terminology and definitions, heterogeneity exists in research approaches, telehealth strategies, data, and reporting, ultimately leading to clinical challenges with implementation and decision-making due to missing or lacking data [8]. An example of this can be seen in studies reviewing telehealth in amyotrophic lateral sclerosis (ALS) care. In 2019, Helleman et al [9] conducted a systematic review on the use of telehealth in ALS care. The authors concluded that telehealth is positively received by patients and carers; however, care teams report mixed feelings for telehealth in ALS care. This is due to implementation barriers surrounding legislation and finances and the need for more data on barriers/facilitators and cost-effectiveness in this space, demonstrating the effects of missing pertinent data [9].

In addition to the identified lack of data exploring the cost-effectiveness of telehealth, in 2022, Houser et al [10] identified a lack of data and research on telehealth practices to manage documentation of telehealth patient visits (eg, the clinical, administrative, and technical information that must be entered into a patient health record). The authors collected survey data from clinical administrators and managers of different physical and mental health facilities in the United States and found that there are inequities in patient access and knowledge of technology, telehealth guidelines are being frequently updated, and telehealth procedures are also being frequently updated, causing difficulty for both patients and care teams. More notably, there are barriers related to associated documentation for reimbursement from health insurers, leading to refusal of telehealth visits and other associated legal issues, again presenting an example of how a lack of data on documentation practices affects telehealth implementation and delivery [10].

These issues with associated telehealth documentation were also found by research conducted in 2017 to explore patient information leaflets on telehealth. Kayyali et al [11] identified information gaps in these leaflets, such as technical assurance, technical support, cost, privacy, safety, and patient choice, related to information about technology reliability, what to do if the technology failed, whom to contact with technical issues, the costs associated with a telehealth visit, and how privacy and confidentiality will be maintained. These information gaps were also believed to contribute negatively to patient experience and the adoption of telehealth services, again demonstrating the need for data to support meaningful and informed patient information leaflet creation as part of telehealth implementation [11].

As patients expressed concern with privacy and safety [11], so too did researchers and care teams. In 2022, Houser et al [12] conducted a complementary systematic review exploring data and privacy concerns in US telehealth research. The authors found that these concerns are caused by three types of key factors: environmental, technological, and operational (eg, broadband infrastructure disparities, the integration of telehealth platforms into electronic health records [EHRs], and staff training). The authors concluded that broad approaches considering multiple domains (technology, digital literacy, accessibility, privacy and safety concerns) are needed to develop best-practice guidelines for the implementation of telehealth into services, presenting an example of pertinent telehealth data that need to be included in telehealth research to support wider synthesis for the development of guidelines [12].

These disparities have continued to be noted across health care services in more recent years. In 2024, Amagai et al [13] retrospectively analyzed EHR data from a large hospital network in the United States to understand telehealth disparities across specialties. The authors noted that telehealth reduces the number of no-shows (did not attend) overall, telehealth use and effectiveness vary depending on specialty, and there are disparities among demographic groups (including race, ethnicity, health insurance type, and access to technology). Moreover, telehealth alone does not eliminate health inequities, and pre-existing structural barriers can persist in telehealth. The authors highlighted that policy makers need to tailor policies to reach different patient cohorts, address the varying specialty needs, and improve equitable telehealth adoption [13], presenting an example of how a “one size fits all” approach to telehealth does not work and more data are needed to understand and combat health inequities.

In 2025, the aforementioned barriers and considerations were continuously noted. For example, Mahdavi et al [14] reported that successful telehealth implementation is strongly influenced by infrastructure, technical support, and training for patients. Oudbier et al [15] built upon this by demonstrating that implementation is not a singular process and relies on ongoing management and review to adapt to the changing needs of patients and care services. In addition, two systematic reviews [16,17] concluded that telehealth is a beneficial component to care delivery, although technology gaps, regulatory uncertainties, and equity issues are ongoing, and that a collaborative approach is needed to develop sustainable and equitable telehealth frameworks that detail comprehensive training programs and investment in technology. Another 2025 systematic review [1] focusing on telehealth during the COVID-19 pandemic identified that evaluations lacked adequate methods and cohort coverage. These telehealth systems, due to the necessity of the pandemic, were rapidly implemented without sufficient data on effectiveness and implementation pathways. Consequently, Mumporeze et al [1] called for future studies to provide more comprehensive data to describe these pathways. These examples demonstrate how research and telehealth inductive reviews vary in what data are found to be important; however, all studies show that data that support frameworks and pathways are essential for sustaining telehealth.

The aforementioned telehealth studies demonstrate that there are important gaps in the evidence base, especially missing or inconsistently reported data, and that certain data play a critical role in guiding long‑term adoption, informing policy development, and shaping implementation strategies that support equitable access and workflow integration. To address these gaps and inconsistencies, emerging standardized reporting methods now provide a means to consistently capture essential research and implementation data, ensuring reproducibility and strengthening the quality of evidence needed to support telehealth scale‑up and policy formation.

### Rationale and Scoping Review Aims

From an implementation science perspective, closing the gap between what we know and what we do becomes difficult when the evidence base is insufficient. In the aforementioned systematic reviews, the authors deduced missing or important telehealth data from the identified literature in a bottom-up approach. However, there is need for a deductive and structured top-down approach that can be replicated across multiple telehealth care contexts to provide a means of identifying both the strengths and the weaknesses of telehealth reporting in a standardized way. In short, previous systematic reviews have worked from the ground up to piece together what is missing and important, and there is need for novel structured top-down approaches to support reproducibility in identifying what is being reported well and what is missing.

This scoping review sought to use such a structured approach to provide a more complete picture of the current state of evidence and identify areas for improvement in the reporting of telehealth implementation projects. To do so, we examined the extent of telehealth implementation for cystic fibrosis (CF) reported up to 2025 using a novel method that leverages standardized reporting frameworks and a theory-based evaluation (TBE) perspective. The findings of this review will offer a framework that can be applied to other chronic diseases.

To achieve the overall study aim, we:

- Conducted a systematic scoping review of available research on telehealth in CF care
- Examined the reporting of telehealth implementation in the identified studies against a standardized reporting frameworks
- Identified the knowledge gaps and strengths of the existing literature

### Implementation of Telehealth in Cystic Fibrosis

CF is a specialized multisystem chronic genetic condition, and its complexity necessitates support from a specialized multidisciplinary care team spanning medicine, nursing, dietetics, physiotherapy, pharmacy, psychology, and social care. Care teams must also regularly engage with additional specialist services (eg, endocrinology, nephrology, cardiology, dermatology, gastroenterology, radiology and imaging, and mental health) to address the diverse complications that can affect multiple organs and give rise to comorbidities, such as liver disease, diabetes, and pancreatic insufficiency [18]. The availability of highly effective modulator therapies has further shifted the clinical landscape, extending life expectancy for many patients with CF and further incorporating other areas of care, such as pregnancy, aging, menopause, and malignancies [19]. As a result, CF care teams now also collaborate more frequently with broader specialties, such as fertility and maternity care, and oncology [20,21].

Given the multifaceted nature of the condition and the need for specialized multidisciplinary teams, telehealth plays an important role in CF care by enabling timely access to specialized support, improving disease management for patients living far from specialist centers, and supporting continuity of care through virtual consultations and remote monitoring [22]. Remote monitoring and telehealth have been explored in CF care since the 1980s [23], offering a long history of engagement with digital health approaches. As such, CF provides an especially relevant model for telehealth implementation.

CF has a clearly defined patient population, which mirrors many chronic conditions that rely on specialist services. This defined cohort brings its own considerations for how telehealth can facilitate disease management and support individuals who are geographically dispersed or distant from specialist centers. CF care also involves long‑term follow‑up across well‑established clinical outcome measures, such as lung function, microbiology, and surveillance for complications [24,25]. CF also carries a substantial burden of treatment; patients with CF typically require three to four reviews annually with specialist centers and must manage a high volume of regular prescribed medications, many of which demand ongoing monitoring [26].

Finally, CF benefits from established international models of care [27] that guide practice in a consistent and uniform manner across geographic locations, creating a stable framework within which telehealth innovations can be tested, implemented, and evaluated. In Australia, telehealth has become so integrated into standard care that it is written into the national standards of care [28].

As across other disease areas, the implementation and integration of telehealth in CF care was especially amplified during the COVID-19 pandemic, with many practice-based examples of its use during that time being published [8]. Since then, although a return to face-to-face care has prevailed [29], an ongoing interest in the potential of telehealth implementation in CF care has remained, with some surmising that future face-to-face clinics may not always be needed [30], paving the way for greater integration of telehealth and other digital health solutions into standard CF care delivery. Furthermore, two large CF networks (the Cystic Fibrosis Learning Network [CFLN] in the United States and the European CF Society Telehealth for CF Working Group [THCF]) are actively exploring safe and equitable implementation into CF care delivery, also presenting an opportunity for wider contextual understanding [4,31].

Although interest in telehealth for CF care continues, the evidence base remains limited, and the absence of implementation standards restricts its broader adoption [32]. Many promising examples exist [8], often as case studies, but systematic reviews show most research focuses on feasibility rather than outcomes [33,34]. Inconsistencies in study design, limited multicenter trials, and no standardized reporting make it difficult to establish best practices for integrating telehealth into CF care and clinical decision-making [8].

## Methods

### Defining a Theoretical and Reporting Framework

We followed a TBE approach for this study. TBE is a method of judging how well programs, such as telehealth, are working, focusing on how they are implemented and the results they achieve [35]. TBE is based on two key ideas [36]:

- Program theory: This explains the assumptions about how a program is supposed to work to achieve its intended goals, or outcomes.
- Implementation theory: This looks at the activities involved in running a program, or how a group carries it out.

**Figure 1:**
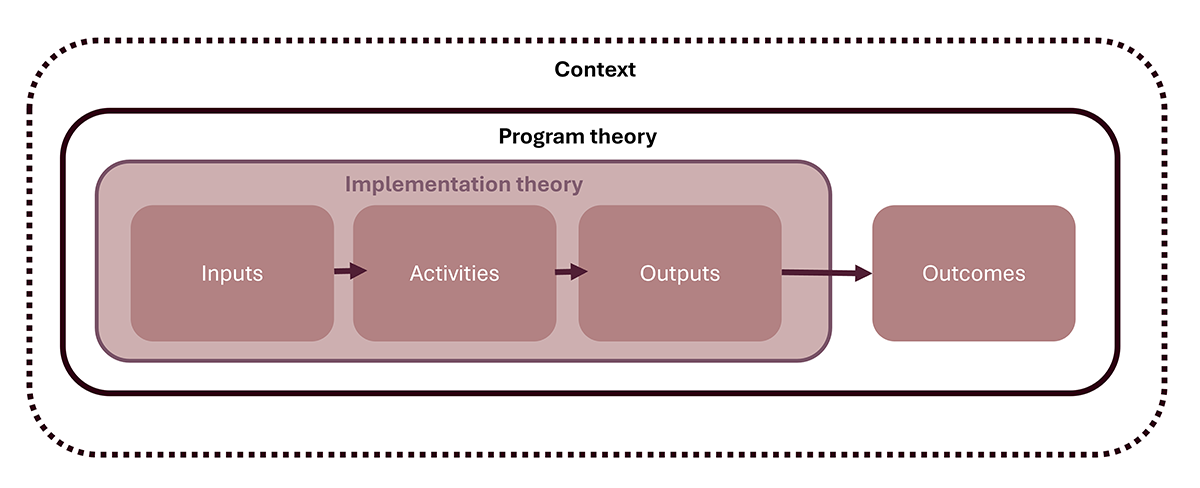
Relationship between implementation theory, program theory, and context.

TBE also considers the role of context and how different settings or conditions can help or hinder the program’s success. It examines the mechanisms (processes or structures) that influence whether a program achieves its goals or has unintended effects (see Figure 1) [37]. By combining these ideas, TBE supports evaluations that look at both outcomes (what was achieved) and implementation (how it was done) in context. This approach helps build evidence about what works, for whom, and in what situations. In the case of telehealth for conditions such as CF, TBE is therefore a valuable tool to fill gaps in the evidence.

With TBE as a guiding principle, two reporting frameworks were selected from the Equator Network database [38] that individually deal with different aspects of implementation theory, program theory, and the context related to telehealth interventions: Guidelines and Checklist for the Reporting on Digital Health Implementations (iCHECK-DH) and Template for Intervention Description and Replication for Telehealth (TIDiER-telehealth). Collectively, the chosen reporting frameworks identify core components of both implementation theory and program theory.

iCHECK-DH [39] (referred to as iCHECK in this study) was published in 2023 and is a digital health implementation reporting tool. Although this framework does not deal specifically with telehealth interventions, it does capture the factors potentially affecting the implementation of telehealth interventions (see the *iCHECK Checklist* section in Multimedia Appendix 1).

TIDiER-telehealth [40] (referred to as TIDiER in this study) published in 2022, is a framework focused on reporting telehealth interventions in outcome-driven studies. It emphasizes detailing the intervention and its outcomes (i.e., program theory) but does not primarily address implementation reporting (see the *TIDieR-Telehealth* section in Multimedia Appendix 1).

Each framework details a standardized reporting checklist. For this review, we reverse-engineered the aforementioned checklists to extract data from the selected studies to describe what information is being reported within this space and to identify what knowledge gaps exist.

### Protocol

In 2023, we undertook a systematic review exploring CF telehealth studies [8] up to May 2021. This scoping review protocol is an extension of the previous systematic review and is not registered. The original systematic review methodology was repeated to gather more recent studies (May 2021-May 2025). This scoping review was reported in accordance with the Preferred Reporting Items for Systematic Reviews and Meta-Analyses Extension for Scoping Reviews (PRISMA-ScR) checklist (Multimedia Appendix 2) [41].

### Eligibility Criteria

The inclusion criteria for the systematic refinement of studies included only scientific papers available in English. Conference abstracts, reviews, and gray literature were excluded from this review. Papers meeting the following criteria were included for review:

- Population: telehealth services delivered to CF populations or reviewed by CF care teams
- Concept: the implementation of any telehealth intervention adhering to the telehealth definition outlined by the THCF [4]:

Telehealth provides safe and accessible care/education through remote consultations between a patient/caregiver and healthcare professional using communication technology (e.g., videoconferencing, smartphone). It can be further enhanced using remote patient monitoring technologies coupled with safe and meaningful digital data transfer and storage.

- Context: telehealth interventions delivered alongside and/or as part of routine CF care

### Information Sources and Search

The search methods used were reported following the Preferred Reporting Items for Systematic Reviews and Meta-Analyses Extension for Reporting Literature Searches (PRISMA-S) checklist (Multimedia Appendix 3) [42]. The databases selected for this review included PubMed, Scopus, and Web of Science as they offer complementary strengths. Although all three databases index similar disciplines and research fields, PubMed was chosen as it predominantly indexes clinical, biomedical, and medical content. Scopus was chosen as it builds upon this to also include technology, business, economics, and finance journals. Finally, the Web of Science was chosen as it indexes many computer science, engineering, and technology journals.

The search string included was “Cystic Fibrosis” and one of the following terms, as described in the original systematic review: “Virtual Care,” “Virtual Health,” “Remote Monitoring,” “TeleMonitoring,” “Digital Health,” “Home Monitoring,” “eHealth,” “telehealth,” “telemedicine”, “wearable devices” [8].

Here is an example of the search string used for the Web of Science database:

“TS=(Cystic Fibrosis AND Virtual Health) OR TS=(Cystic Fibrosis AND Remote Monitoring) OR TS=(Cystic Fibrosis AND TeleMonitoring) OR TS=(Cystic Fibrosis AND Digital Health) OR TS=(Cystic Fibrosis AND home Monitoring) OR TS=(Cystic Fibrosis AND ehealth) OR TS=(Cystic Fibrosis AND telehealth) OR TS=(Cystic Fibrosis AND telemedicine) OR TS=(Cystic Fibrosis AND wearable devices) OR TS=(Cystic Fibrosis AND Virtual Care)”

For a full list of search strings for each database and how these strings were developed, see the *Search Strategy* section in Multimedia Appendix 1.

Other search methods, such as simultaneous multidatabase searching, study registry search, purposeful online browsing, citation searching, or contact with researchers, were not used in this search strategy.

### Selection of Sources of Evidence

Once all sources of evidence were identified, duplicates were removed using formulas in Microsoft Excel. Titles and abstracts were reviewed to ensure papers met the inclusion criteria, as outlined before. Two reviewers (authors TV and RD) blind-reviewed the papers during each stage and resolved any conflicts through consensus discussions at each of the title, abstract, and full-text review stages. Consensus discussions centered primarily on whether the interventions included met the definition of telehealth, as defined before.

### Data Charting and Data Items

The data selected for analysis included (1) study descriptive and (2) checklist data. Study descriptive data included the paper title, author list, year of publication, and journal. For data extraction, all 20 items from iCHECK [39] and 12 items from TIDiER [40] were used (see the data extraction template in Multimedia Appendix 4). The studies were scored against each category available in each checklist: fully reported on a category, score=2; partially reported on a category, score=1; and did not report, score=0. All extracted data were stored in Excel. The same scoring system was used for all category items, and no weighting was provided to any specific category. Although some categories may be seen as more relevant for practical implementation (eg, safety and data governance) and others are not mandatory (as per the checklists), we sought to identify reporting strengths and weaknesses only. Hence, an unweighted category–scoring approach was adopted.

See Table 1 for an example of scoring TIDiER category 8 (When and How Much: “Describe the number of times the intervention was delivered and over what period of time including the number of sessions, their schedule, and their duration, intensity or dose.”)

**Table 1:** Example of “fully reported,” “partially reported,” and “did not report” scoring for one checklist category.

Scoring label (score)	Study	Data example
Fully reported (2)	Graziano et al [43]	“The intervention consisted of four individual telehealth sessions, each lasting 30-40 minutes, delivered via online video platforms. The sessions were conducted during the COVID-19 lockdown in Italy, starting in March 2020. Participants received all four planned sessions. Feasibility and satisfaction were assessed one week after the last session.”
Partially reported (1)	Verkleij et al [44]	“The intervention consists of eight 45-minute sessions. The initial intake, fourth, and last sessions are completed in real time with a therapist, while the remaining five sessions are self-guided. The paper does not specify the exact frequency or total duration over which these sessions are delivered.”
Did not report (0)	Hasan et al [45]	“Not mentioned (the paper does not provide specific details on the number of sessions, their schedule, or duration for the telehealth intervention)”

Data extraction and scoring were performed using Elicit AI Research Assistant 2024 [46]. Elicit is an artificial intelligence (AI) tool that uses large language models (LLMs) to extract data from scientific studies and is reported as being 80%-90% accurate [47]. To extract data, the AI tool is fed prompts that include a label and a description of which data are to be extracted. For this review, we tused he category names and definitions, as described within the checklists, to inform the AI tool. The category names within the checklist were used as data extraction “labels,” and the category definitions from the checklist were used as “label descriptions” (Multimedia Appendix 5).

### Validation of AI Data Extraction

To evaluate the accuracy of the AI tool, two reviewers (TV and RD) also manually extracted and scored data from 18 studies (Table S1 in Multimedia Appendix 6). We calculated the difference between the manual and AI scoring for each paper and each checklist category. No disagreement in scoring was equal to a difference of 0, a minor disagreement was equal to a difference of 1, and major disagreement was equal to a difference of 2. The frequency of “no disagreements,” “mnor disagreements,” and “major disagreements” was calculated (Figure S1 and Figure S2 in Multimedia Appendix 6). We found that the title category had the highest number of major disagreements (5 for iCHECK and 12 for TIDiER), which was found to be related to various digital health terms available to describe telehealth (eg, telemedicine, telecare, and telemonitoring). However, for all other checklist categories, and per manuscript comparisons, the number of major disagreements was low (≤3), with the majority of values being 0 (Figure S1 and Figure S2 in Multimedia Appendix 6). Therefore, Elicit AI was found acceptable for this review.

### Critical Appraisal of Individual Sources of Evidence

Due to the heterogeneity of the studies and variability in associated study data, a quality appraisal tool was not implemented for this review. Instead, we focused on the quantity of information reported according to the iCHECK and TIDiER checklists.

### Synthesis of Results

The maximum total score that a paper can receive with iCHECK is 40 (ie, 20 categories with a maximum score of 2 per category) and with TIDiER is 24 (ie, 12 categories with a maximum score of 2 per category). This maximum score represents a paper that fully reports on all checklist categories, scoring 2 in each category. A paper such as this would be described as 100% adherent to the reporting checklist.

All papers and their checklist scores were stored in Excel. Descriptive statistics were performed on each of the manuscript scores and checklist categories.

### Ethical Considerations

This study did not involve human participants.

## Results

### Selection of Sources of Evidence

**Figure 2:**
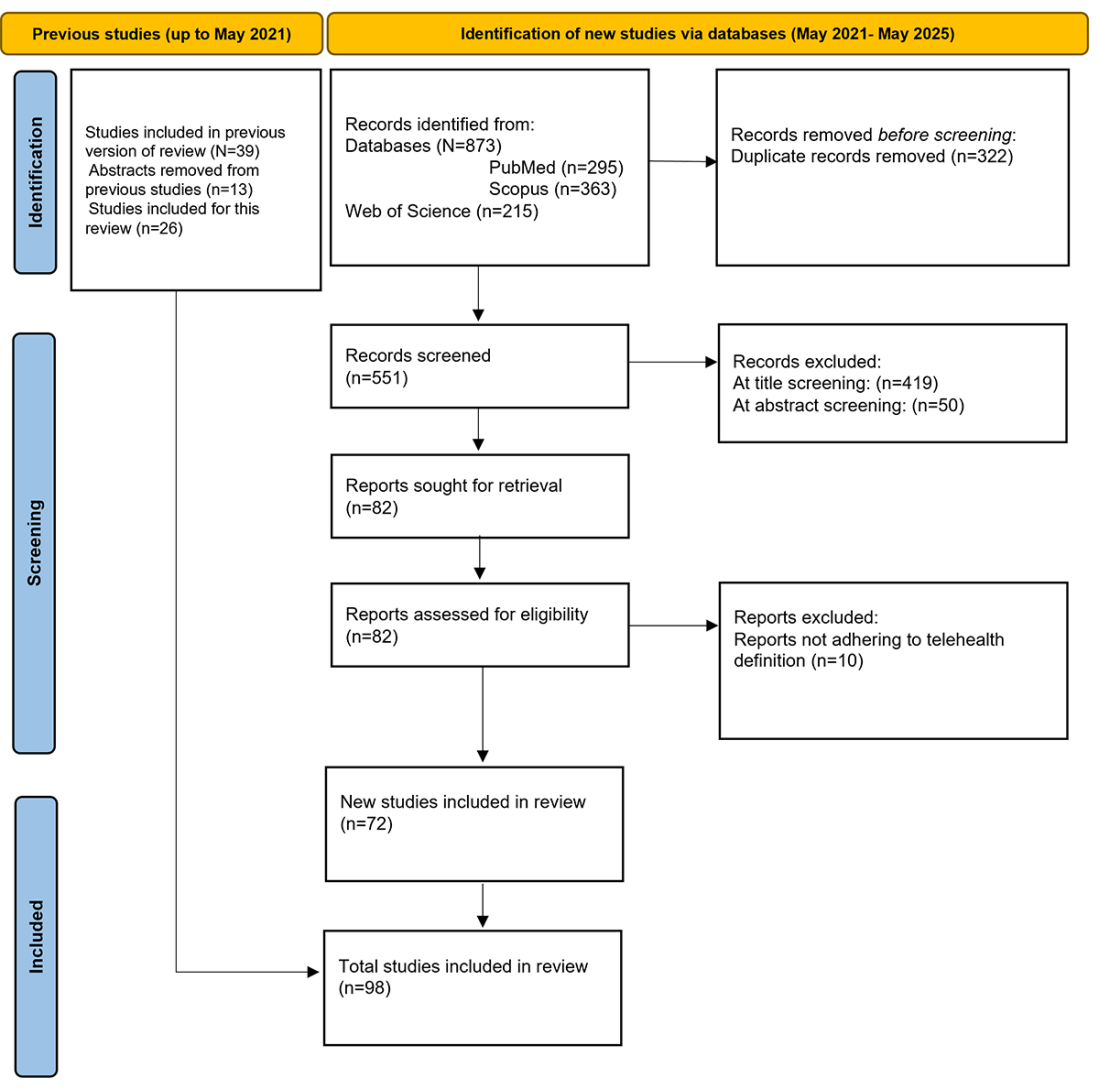
PRISMA flow of the paper selection process using a combination of two reviews. The first considered papers from the beginning to May 2021 (n=26), and the updated review took place from May 2021 to May 2025. PRISMA: Preferred Reporting Items for Systematic Reviews and Meta-Analyses.

From the original systematic review up to May 2021 (N=39), 13 (33%) conference abstracts were removed for this review. In the second phase of the systematic review (May 2021-May 2025), 873 new studies were identified. From this pool, 322 (36.9%) duplicates were removed. The remaining 551 (63.1%) studies underwent systematic refinement, and 419 (76.0%) studies were excluded at title screening, 50 (9.1%) at abstract screening, and 10 (1.8%) at introduction screening, leaving 72 (13.1%) studies that met the inclusion criteria. The 26 studies from the original systematic review and these 72 studies from this scoping review constituted a total of 98 studies from the beginning until May 2025 (see Figure 2). A full list of the final 98 studies [30,43-45,48-141] can be found in Table S2 in Multimedia Appendix 6.

### Characteristics of Sources of Evidence

A summary of the 98 studies (study design, population, country of origin, telehealth format, and key findings) can be seen in Table S3 in Multimedia Appendix 6. Of the 98 studies, 90 (91.8%) were conducted in a single country. The United States (n=42, 46.7%), Australia (n=11, 12.2%), and the United Kingdom (n=8, 8.9%) were the three most common countries. In addition, 6 (6.1%) studies were conducted in two or more countries, and 2 (2.0%) studies did not explicitly describe the country where they were conducted, and this information had to be inferred from author affiliations and ethics committee details. The earliest study was published in 2006 (n=1, 1.0%), with the highest number of studies being published during the pandemic (2021: n=28, 28.6%; 2022: n=16, 16.3%; Figure S3 in Multimedia Appendix 6). Furthermore, 79 (80.6%) studies were published in medical journals and the remaining 19 (19.4%) in digital health journals (Figure S4 in Multimedia Appendix 6). Of the 98 studies, 9 (9.2%) used the term “intervention” within the title and 3 (3.1%) used the term “implementation” (Figure S5 in Multimedia Appendix 6). We found 17 digital health terms used within the titles to describe the telehealth service: “telehealth” (n=41, 41.8%), “telemedicine” (n=14, 14.3%), and “home spirometry” (n=8, 8.2%) were the most common digital health terms used within the titles (Figure S6 in Multimedia Appendix 6). In addition, 80 (81.6%) studies used one digital health term, 10 (10.2%) studies used two terms, and 8 (8.2%) studies used no digital health terms. An example of a title with no digital health terms was “Understanding the Acceptability of the Changing Model of Care in Cystic Fibrosis” [136]. The studies used various telehealth modalities in various combinations (eg, video calls in combination with remote monitoring). The most common telehealth modalities included video calls only (n=22, 22.4%); video calls, phone calls, and remote monitoring (n=20, 20.4%); video calls and remote monitoring (n=16, 16.3%); and phone calls and remote monitoring (n=11, 11.2%).

The population focus across the studies varied from adults with CF, adolescents with CF, and children with CF to their parents/caregivers, partners, and CF care teams, with many of the studies exploring a combination of these populations (eg, adults with CF and parents; adults with CF and CF care teams; or children with CF, adolescents with CF, and parents). Multiple study designs had been used across the 98 papers. The most common study types included cross-sectional studies (n=17, 17.3%), qualitative studies (n=13, 13.3%), randomized control trials (n=9, 9.8%), and observational studies (n=8, 8.2%).

### Synthesis of Results: Standardized Reporting Scores

#### Overall Scores

Table S4 (iCHECK) and Table S5 (TIDiER) in Multimedia Appendix 6 provide a full list of category scores for each of the 98 studies (score=0 is marked in red to assist in visualizing the gaps). The median score calculated for all studies was 22/40 (range 11-29, 55.0% adherent) for iCHECK and 15/24 (range 6-23, 62.5% adherent) for TIDiER.

#### Category Scores for iCHECK

For iCHECK, across the 20 checklist categories, 11 (55%) scored ≥50% for adherence. Of the remaining 9 (45%) categories, 2 (22.2%) scored <10% (as shown in Table 1). In terms of reporting completeness, we calculated percentage scores across “fully reported,” “partially reported,” and “did not report.” For “fully reported,”, 6 (30%) categories scored ≥50%, while 4 (20%) scored of 0. For “partially reported,” 9 (45%) categories scored ≥50%, with 1 (5%) scoring <10%. Finally, for “did not report,” 3 (15%) categories scored ≥50%, with 3 (15%) other categories scoring 0 (Table 2; also see Table S6 and Figure S7A in Multimedia Appendix 6).

**Table 2:** Summary of scores for the iCHECK^a^ checklist.

Categories			Adherence level (%)^b^		Overall adherence to each category (total score), n (%)		Percentage of papers (number) that:				
							Reported completely, n (%)		Partially reported, n (%)		Did not report, n (%)
**Title**											
	Title	Moderate		147 (75.0)		58 (59.1)		31 (31.6)		9 (9.2)	
**Abstract**											
	Abstract	Very good		180 (91.8)		85 (86.7)		10 (10.2)		3 (3.0)	
**Introduction**											
	Context	Moderate		103 (52.5		5 (5.1)		93 (94.9)		0	
	Problem statement	Very good		179 (91.3		81 (82.6)		17 (17.3)		0	
	Similar interventions	Moderate		98 (50.0)		11 (11.2)		76 (77.5)		11 (11.2)	
**Methods**											
	Aims and objectives	Moderate		134 (68.4)		46 (46.9)		42 (42.8)		10 (10.2)	
	Blueprint summary	Moderate		125 (63.7)		46 (46.9)		37 (37.7)		17 (17.3)	
	Technical design	Low		62 (31.6)		2 (2.0)		58 (59.1)		38 (38.7	
	Target	Very good		187 (95.4		89 (90.8)		9 (9.2)		0	
	Data	Low		58 (29.6)		0		58 (59.2)		40 (40.8)	
	Interoperability	Very low		15 (7.6		0		15 (15.3)		83 (84.7)	
	Participating entities	Low		76 (38.7)		1 (1.0)		74 (75.5)		23 (23.4)	
	Budget planning	Very low		12 (6.1)		0		12 (12.2)		86 (87.8)	
	Sustainability	Low		44 (22.4)		0		44 (44.9)		54 (55.1)	
**Results**											
	Coverage	Moderate		87 (44.3)		10 (10.2)		67 (68.3)		21 (21.4)	
	Outcomes	Very good		162 (82.7)		75 (76.5)		12 (12.2)		11 (11.2)	
	Lessons learned	Moderate		96 (48.9)		1 (1.0)		94 (95.9)		3 (3.0)	
	Unintended consequences^c^	Moderate		100 (51.0)		20 (20.4)		60 (61.2)		18 (18.3)	
**Discussion**											
	Conclusion	Very good		165 (84.1		79 (80.6)		7 (7.1)		12 (12.2)	
**General**											
	General^c^	Moderate		118 (60.2)		32 (32.7)		54 (55.1)		12 (12.2)	

^a^iCHECK: Guidelines and Checklist for the Reporting on Digital Health Implementations.

^b^Adherence level (by % range): very low, 0%-20%; low, 20%-40%; moderate, 40%-60%; good, 60%-80%; very good, 80%-99%.

^c^These categories are not mandatory within the original checklist.

#### Category Scores for TIDiER

For TIDiER, across the 12 checklist categories, 8 (66.7%) scored ≥50%. Of the remaining 4 (33.3%) categories, none scored <10% (as shown in Table 1). In terms of reporting completeness, 4 (33.3%) categories scored ≥50%, while 1 (2.8%) scored <10% for “fully reported.” For “partially reported”, 6 (50.0%) categories scored of ≥50%, with 2 (16.7%) scoring <10%. Finally, for “did not report,” 1 (2.8%) category scored ≥50%, with 5 (41.7%) scoring <10% (Table 3; also see Table S6 and Figure S7B in Multimedia Appendix 6).

**Table 3:** Summary of scores for the TIDiER^a^ checklist.

Categories		Adherence level (%)^b^	Overall adherence to each category (total score), n (%)	Percentage of papers (number) that:		
				Reported completely, n (%)	Partially reported, n (%)	Did not report, n (%)
Brief name		Low	70 (35.7)	35 (35.7)	0	63 (64.2)
Why		Very good	176 (89.8)	81 (82.7)	14 (14.3)	3 (3.1)
**What**						
	What (materials)	Moderate	113 (57.7)	19 (19.4)	75 (76.5)	4 (4.1)
	What (procedures)	Very good	174 (88.8)	84 (85.7)	6 (6.1)	8 (8.2)
Who provided		Low	66 (33.7)	5 (5.1)	56 (57.1)	37 (37.8)
How		Very good	181 (92.3)	83 (84.7)	15 (15.3)	0
Where		Good	157 (80.1)	60 (61.2)	37 (37.8)	1 (1.0)
When and how much		Moderate	109 (55.6)	23 (23.5)	63 (64.2)	12 (12.2)
Tailoring^c^		Moderate	103 (52.5)	27 (27.5)	49 (50.0)	22 (22.4)
Modifications^c^		Low	75 (38.3)	23 (23.5)	29 (29.6)	46 (46.9)
**How well**						
	How (planned)^c^	Moderate	92 (46.9)	16 (16.3)	60 (61.2)	22 (22.4)
	How (actual)^c^	Moderate	102 (52.0)	16 (16.3)	54 (55.1)	10 (10.2)

^a^TIDiER-telehealth: Template for Intervention Description and Replication for Telehealth.

^b^Adherence level (by % range): very low, 0%-20%; low, 20%-40%; moderate, 40%-60%; good, 60%-80%; very good, 80%-99%.

^c^These categories are not mandatory within the original checklist.

## Discussion

### Principal Findings

This scoping review examined how telehealth interventions for CF are reported using a TBE perspective with two established frameworks (TIDieR and iCHECK). To the best of our knowledge, this is the first review to use both TIDiER and iCHECK reporting standards in a structured deductive approach to assess the completeness of information reported in the telehealth literature. We found that although studies have consistently described why telehealth is used, whom it targets, and what outcomes are achieved, far less details are provided regarding how telehealth interventions are implemented in practice. Reporting related to technical design, data handling, interoperability, budget planning, sustainability, and operational modifications is notably limited. These gaps restrict our understanding of implementation processes and hinder the ability to interpret or replicate models of telehealth care across different settings. These findings describe deficiencies within telehealth reporting, not deficiencies within the intervention itself or how telehealth was implemented within each study’s context. These reporting deficiencies highlight that reproducing these results in other contexts will be challenging.

### Interpretation of Findings in Relation to the Existing Literature

The use of TBE to underpin the choice of reporting frameworks used in this review highlights gaps in understanding the implementation of telehealth interventions. By integrating program theory, implementation theory, and context, TBE guided us to address the extent of outcome reporting (what was achieved), implementation (how it was done), and contextual factors.

When comparing the two checklists, studies showed slightly greater adherence to the TIDiER (intervention) checklist than to the iCHECK (implementation) checklist. This suggests that available data more often frame telehealth as an isolated intervention rather than as a fully implemented care service pathway. Across both checklists, researchers consistently explain why telehealth is being used, identify target populations, and report study outcomes effectively. This provides valuable insight into the impact of telehealth within specific contexts. However, the most underreported areas relate to the practical aspects of implementation, indicating that evidence-based guidance on how to operationalize telehealth remains limited or unclear.

Technical design, data, interoperability, participating entities, budget planning, sustainability, brief name, who provided, and modifications were some of the lowest scoring categories across both checklists. Interoperability, budget planning, brief name, and modifications received the highest incidences of “did not report”. Except “brief name,” each of the other categories has a potential impact on implementing telehealth into clinical practice. One of the most discussed issues in the wider telehealth literature is that of budget planning [9]. Often, it is believed that telehealth can provide some reductions in cost to the health care system. However, increasing numbers of publications have identified that there may not be any cost-saving benefits [48] and that costs may increase during initial implementation and later during routine service delivery [142]. This is further complicated by how telehealth is intended to be used within care delivery, for example, whether as a service that primarily operates on telehealth (with face-to-face meetings being used for exceptional circumstances) or as the more commonly described hybrid models [30]. Hybrid models often use telehealth in tandem with an existing service and can be additive to traditional services [142] and/or can cause overuse of care, which can further increase costs [143]. Understanding the context in which the telehealth service has been implemented, its intended use within the service, and the budget planning to support that would allow other services to interpret the transferability of the research into their own context without potential unforeseen financial pressure.

Sustainability (financial, economic, environmental, etc) was underreported and remains an area for continued discussion due to knowledge and evidence gaps. Within telehealth, environmental impact data can be both positive and negative [144-146]. For example, there is potential for reduced greenhouse emissions from less travel (patients and care teams), lower hospital equipment use, and less waste from sanitization procedures [144-146]. However, generating and operating digital systems increases energy use, and poorly managed recyclable wearable and portable devices create more waste [147]. A more prominent challenge is sustaining telehealth services over time [148,149]. Following the COVID-19 surge, many services reported a decline in use [150]. Research is ongoing to understand this trend, sometimes described as “telehealth fatigue” [148,151]. Addressing it requires detailed best practices and delivery strategies [151]. Yet, without sustainability data in publications, identifying effective, context-specific strategies remains difficult, potentially contributing to further decline and impacting areas such as budget planning.

Telehealth is inherently multidisciplinary, blending health care with computing and engineering. Papers are often authored by health care professionals and published in medical journals, which may explain why technical categories (eg, technical design, data, interoperability, modifications) are underreported. Although many arguments support expanding these areas, two worth highlighting are data security/protection and technology literacy. As technology becomes more embedded in health care, implementing proper safety protocols for managing health data is essential. Poorly managed telehealth can pose serious privacy risks [12], reducing patient and organizational trust, leading to decreased use [152], and affecting service sustainability. Technology literacy (described as the skills, attitudes, and knowledge to use technology confidently) is another barrier to telehealth adoption and influences health needs [153]. A lack of digital health literacy (technology and health literacy combined) can worsen existing health inequalities and increase the burden on care teams [154-156]. To improve digital health literacy and data security awareness, better reporting of these categories in telehealth studies is needed.

Finally, the COVID-19 pandemic caused an increase in the adoption, use, and distribution of scientific publications describing telehealth [157,158]. Now, the focus is moving from rapid adoption to consideration of implementation barriers and telehealth sustainability challenges [16,159]. Considering this, it is important that future research in this space continue to be published in adherence to a standardized checklist. This will not only provide more robust and consistent evidence for the use of telehealth but also data to allow care services to interpret and transfer this knowledge into their own context in a relevant, safe, and meaningful way.

### Clinical Implications of the Current Evidence Base

Telehealth interventions in the selected studies were described at a clinical level (whom they target, why they are used, and what outcomes they achieve), but the practical realities of implementing them in existing care pathways remain insufficiently detailed or negated entirely. This can lead to several implications for clinical practice. First, without information about workflows, staff roles, technical infrastructure, and required modifications to existing processes, health care teams cannot reliably anticipate the operational impact of adopting a telehealth model. This increases the risk for failed or unsustainable implementations [148,149].

Second, the underreporting of budget planning and sustainability prevents clinicians and service managers from evaluating ongoing resource needs, cost pressures, or unintended cost escalations. This gap is clinically important because budget constraints directly influence workforce allocation, service availability, and long-term viability [9,142,143].

Third, limited reporting on data management, interoperability, and digital literacy considerations impedes care teams’ ability to assess whether the telehealth approach protects patient privacy, accommodates patients with low digital health literacy or access to technology and Wi-Fi (avoiding digital exclusion as part of the digital divide), or exacerbates health inequalities already caused by socioeconomic biases and geographic disparities [12,154-156].

Fourth, without contextual implementation data, clinical leaders cannot distinguish whether an observed improvement (eg*,* reduced admissions, improved adherence) is due to the telehealth model itself or due to local adaptations, resource differences, or specific contextual enablers [16,160].

Finally, insufficient details on modifications, technical design, and operational processes undermine the transferability and scalability of successful telehealth approaches. This slows down evidence uptake and perpetuates fragmented service models [12,161].

### Limitations

First, there are no universally accepted terms and definitions within the digital health space [2,3]. Furthermore, due to the growing interest in said space, more terms are emerging. As a result, the search strategy in this review (10 terms to describe telehealth as per the outlined definition) can be expanded to include other terms with the potential to identify other studies in the future. Similarly, other databases can also be incorporated to widen the search. This review was limited to publications written in English. We also noted that some of the publications in this review predate the selected checklists and also may have been unknown to the authors of more recent publications. This delay in knowledge transfer and research dissemination is common and somewhat expected, as multiple studies have shown that awareness and uptake of new guidelines often lag for years despite active dissemination efforts [162]. Hence, we did not aim to describe how adherent any individual study is to these checklists and instead focused on knowledge gaps across the literature. It should also be noted that some of the categories in the checklists are “not mandatory” (see Tables 2 and 3) and therefore not essential; however, for this review, these were included.

In conceptualizing and structuring this review, the reporting frameworks identified were individually found to be lacking important aspects of TBE and telehealth. The decision to use two frameworks overcame this issue to a certain extent by attempting to collectively present a more comprehensive picture of reporting of the implementation of telehealth interventions.

Furthermore, many of the included studies did not describe their research as either an intervention outcome study or an implementation study, and as such, it was necessary to score all studies against both checklists to remove potential bias.

This translational challenge of intervention versus implementation in the digital health field is becoming increasingly recognized and explored [163]. Projects such as the European Digital Health Technology Assessment framework (EDiHTA) [164] are specifically developing evaluation methodologies, which may, in time, improve the evaluation of digital health tools and the reporting seen in digital health studies.

The use of both manual and AI scoring of studies highlights the challenges in implementing these kinds of frameworks objectively. For example, in manually scoring the papers against each of the frameworks, we noted the need to clarify category definitions to ensure a consistent scoring mechanism was used. Likewise, the AI tool struggled with more simple categories, such as identifying whether the paper titles reported were linked to telehealth. This was mainly seen within the “title” and “brief name” categories, where AI adopted a lenient scoring method identifying papers using various terms to describe telehealth (telemedicine, remote monitoring, virtual consultation, etc). However, manual scoring found that the terms were too broad and varying and many did not use the term “telehealth” or “implementation” specifically. Currently, many of the AI tools designed to support research are approximately >80% accurate [47,165], and the discrepancies seen in this study are to be expected. Future research in this area could potentially improve this accuracy by dedicating additional time and expertise to AI prompt engineering.

### Conclusion

As the volume of telehealth research continues to grow, it is increasingly important to evaluate not just its efficacy but also the robustness of its implementation [166-169]. The TBE approach followed in this study provides a novel deductive and structured means to assess this evidence base using the selected checklists. Collective adherence to established reporting frameworks remains inconsistent, which may limit reproducibility and transferability of findings. Encouragingly, certain checklists categories are well documented, offering valuable insights into telehealth’s potential. However, the underreporting of implementation processes persists, in part due to the relative novelty of frameworks such as TIDiER and iCHECK. These findings reflect a deficiency in reporting telehealth implementation research and the potential translation of this knowledge into other contexts. These reporting deficiencies do not reflect a deficiency in the telehealth intervention or the identified authors’ ability to implement telehealth. However, strengthening the commitment to comprehensive, framework-aligned reporting is essential to improve the effective and equitable integration of telehealth into routine care.

Although CF is positioned as a case exemplar, the implications of this work extend beyond a single condition. Many complex chronic diseases, such as chronic obstructive pulmonary disorder (COPD), heart failure, diabetes, and rheumatologic conditions, share similar challenges relating to fluctuating illness trajectories, multidisciplinary care needs, and variability in patient digital readiness. As such, the findings of this study are also applicable widely to other chronic conditions. The potential impact of the identified underreported sections will carry a similar impact in other telehealth care cases and need to be considered by all when reporting on telehealth implementations so as to strengthen telehealth reporting consistency across diverse clinical contexts. Improving transparency around implementation processes, contextual factors, and adaptations not only enhances the comparability of telehealth studies but also supports the more reliable translation of evidence into real-world practice for a broad range of chronic conditions. Finally, the novel method presented in this review is transferable and can be used to evaluate telehealth reporting in other health care settings and specialties.

## Appendix

Multimedia Appendix 1 Guidelines and Checklist for the Reporting on Digital Health Implementations (iCHECK-DH) checklist, Template for Intervention Description and Replication for Telehealth (TIDieR-telehealth) checklist, and search strategy for the review.

Multimedia Appendix 2 Preferred Reporting Items for Systematic Reviews and Meta-Analyses Extension for Scoping Reviews (PRISMA-ScR) checklist.

Multimedia Appendix 3 Preferred Reporting Items for Systematic Reviews and Meta-Analyses Extension for Reporting Literature Searches (PRISMA-S) checklist.

Multimedia Appendix 4 Data extraction template.

Multimedia Appendix 5 Elicit artificial intelligence (AI) prompts.

Multimedia Appendix 6 Manual reviewer data extraction compared with Elicit artificial intelligence (AI) extraction; summary of studies for the review; summary of the final 98 studies, including study design, population, research objective, country of origin, telehealth format, and key findings; scores for all 98 papers for the Guidelines and Checklist for the Reporting on Digital Health Implementations (iCHECK) and Template for Intervention Description and Replication for Telehealth (TIDiER) checklists; summary of scores for iCHECK and TIDiER checklists; iCHECK and TIDiER frequencies of 3 disagreement types recorded per paper and per checklist category for 18 studies; publication frequency based on years; discipline focus of journals (medical and digital health); number of papers that self-described as implementation or intervention in the title; digital health terms used in paper titles; and overall category scores (%) of iCHECK and TIDiER.

## Abbreviations

AI: artificial intelligence
ALS: amyotrophic lateral sclerosis
CF: cystic fibrosis
EHR: electronic health record
GAI: generative artificial intelligence
iCHECK-DH: Guidelines and Checklist for the Reporting on Digital Health Implementations
PRISMA: Preferred Reporting Items for Systematic Reviews and Meta-Analyses
PRISMA-S: Preferred Reporting Items for Systematic Reviews and Meta-Analyses Extension for Reporting Literature Searches
PRISMA-ScR: Preferred Reporting Items for Systematic Reviews and Meta-Analyses Extension for Scoping Reviews
TBE: theory-based evaluation
THCF: Telehealth for CF Working Group
TIDiER-telehealth: Template for Intervention Description and Replication for Telehealth

## Data Availability

Data are presented in the main paper, and data are also provided in Multimedia Appendices available for download, along with the published paper.
