# Supplementary material for: Reporting of Telehealth Implementation in Cystic Fibrosis: Scoping Review Using a Novel Theory-Based Evaluation Lens
Source: J Med Internet Res. 2026 May 22;28:e86194. doi: 10.2196/86194 (PMC13241801; doi:10.2196/86194)
Supplement: Multimedia Appendix 1 [file jmir_v28i1e86194_app1.docx]

**Multimedia Appendix 1**

## iCHECK Checklist

**Checklist Name:** i CHECK-DH: Guidelines and Checklist for the Reporting on Digital Health Implementations

**Link to Equator-Network Entry:** https://www.equator-network.org/reporting-guidelines/icheck-dh-guidelines-and-checklist-for-the-reporting-on-digital-health-implementations/

**Version and (Date Last Updated):** 1 (May 17, 2023)

**Checklist:**

| section |  | Item | Description |
| --- | --- | --- | --- |
| Title | 1 | Title  (M) | Identification as an implementation report, and description of the implementation in the title and/or keywords |
| Abstract | 2 | Abstract  (M^a^) | Provide a summary of the key elements of the implementation report, including a description of the implementation strategy, the intervention, defining the key elements of the implementation and health outcomes and specify the key KPIs/Outputs. We recommend describing the main aspects of the research in the following order: Background - Objectives - Methods - Implementation (Results) - Conclusions - (Optional: Trial Registration). |
| Introduction | 3 | Context  (M) | Describe the geographical areas, organizations, target populations and implementation context. Consider social, cultural, economic, political, health care and organizational barriers, infrastructures and facilitators that may influence implementation elsewhere. Explicitly highlight whether a national digital health strategy exists and whether implementation is aligned with the strategy.  Describe the stage of the implementation (Developing or Adapting Solution / Piloting and Evidence generation / Package and Advocacy /Acceleration / Deploying / Scaling up / Hand over or Complete)^b^. |
|  | 4 | Problem statement  (M) | Description of the health care or public health problem, challenge, or deficiency that the implementation aims to address. (If applicable, include a reference to the 'health system challenge' of the WHO Classification of Digital Health Interventions [1] in the description) |
|  | 5 | Similar Interventions  (M) | Mention whether this implementation was inspired by another existing one, and if so, what is the added value of your intervention, if any, compared to the initial one? And what, if anything, has been done differently? |
| Methods | 6 | Aims and Objectives  (M) | Describe the main objectives and the overall aim of the implementation. Describe how these will be measured using predefined primary and secondary outcome(s) and key performance indicators for this implementation and the expected intervention(s).  *For example: indicators or proxy-indicators measuring direct health outcomes (e.g., HbA1c for diabetic patients); Key Performance Indicators (e.g., number of users, number of users that are properly trained, user satisfaction); Indicator assessing a particular process (e.g., administrative time for patient admission);*  (If there was no evaluation, provide detailed explanation for reasoning) |
|  | 7 | Blueprint summary  (M) | Describe the design and key features of the intervention and key points of the implementation strategy and roadmap. |
|  | 8 | Technical Design  (M) | Reasons for developing or choosing this tool. Does it combine several tools? Provide a brief description of the tool(s) (functionality and architecture) and how it fits into the health enterprise architecture and investment roadmap (if applicable). Indicate whether the solution is based on an existing solution or has been developed or purchased specifically for this intervention.  Describe the type of technology used (e.g., AI applications), license of the technology (open source, free, commercial, IP ownership etc.), include code documentation (if available), link to the application, link to wiki or project website. |
|  | 9 | Target  (M) | The target refers to the focus or recipient of the intervention. It is the specific person, group, system, or problem that the intervention aims to change or improve. The characteristics of the targeted "site(s)" (locations, staff, resources, etc.) for implementation and any eligibility criteria. The population targeted by the intervention and any eligibility criteria. |
|  | 10 | Data  (M) | Describe the data governance, including life cycle (collection, processing, storage, modification, sharing, suppression), the data ownership (mention whether patients actually have access to the data), data protection measures, confidential use of routine data, expected level of data integration, data for research, cross-border data agreement, if any, the applicable legal framework, and how the project complies with it. Data consent: Has patient consent been obtained? Describe the approach to data protection and cybersecurity (e.g. security by design, privacy by design, etc.) and where the data is hosted. (e.g., in-country, cloud based, hybrid model etc.). Describe, if applicable, the government preferences in terms of data policies. |
|  | 11 | Interoperability  (M) | Describe the interfaces (what other systems does the tool connect to) and the standards that were used (which specific ones and rationale of choice) (e.g., semantic ontologies such ICD as SNOMED, LOINC or technical standards such as HL7 FHIR, etc.). |
|  | 12 | Participating entities  (M) | Describe the implementing organization(s): Type of organisation(s), mission, leadership, vision, etc.  Government involvement: Describe whether the government was involved in the implementation, at what level and at what stage(s).  Partners: Describe all partners (organisations) and their role in the implementation.  Funders: List all actors and stakeholders who have funded or invested in the development of the implementation (if different from the implementation, e.g. using an existing digital health intervention). Indicate their level of involvement in terms of funding.  Mention which entity will own the final product and intellectual property after the implementation phase. |
|  | 13 | Budget Planning  (M) | Describe the planned budget for implementation (include costs such as change management, user training, project management, technology pricing, total cost of ownership). If possible, include actual costs, otherwise describe the range or percentage of the total budget. Indicate the period covered by the budget. Describe the budget for the intervention (e.g. development, purchase or adaptation of a free tool); if possible include real costs, otherwise describe as a percentage of the total budget. Indicate the duration covered by the budget. |
|  | 14 | Sustainability  (M) | Describe the Business model including the sustainability model (financial, economic, environment etc.). If possible, put outcomes in relation to cost to assess sustainability. Describe long term exit strategies, and all dimensions considered to sustain the project after the end of funding. If applicable, describe potential institutionalization of the project. |
| RESULTS | 15 | Coverage  (M) | Describe whether the coverage of implementation is international, national, regional or at the level of e.g. municipalities. If coverage is sub-national, describe the regions. Provide information on the relative importance of the coverage (e.g. % of eligible population covered). |
|  | 16 | Outcomes  (M) | Primary and other outcome(s) of the implementation. Detail the actual outcomes, using the pre-defined outcome measures (if applicable). |
|  |  | Lessons learned  (M) | Describe any lessons learned from the implementation experience that could be used to improve future outcomes. This could include, but is not limited to, success factors, implementation challenges or budget considerations.  Success factors: Describe factors that positively influenced the implementation (e.g. involvement of key stakeholders). Also describe contextual factors that may have positively influenced the results (e.g. new legal requirements that facilitated adoption).  Challenges to implementation: Describe challenges (process-related, such as resistance to change, but also technical). Include contextual factors that may have affected the achievement of outcomes such as an unexpected change of government, or 'opposing key players' who, despite potential participation, may hinder implementation (e.g. software companies managing regional digital health may act as barriers to innovation).  Budget: Describe whether the implementation budget was adhered to, and if not, why not. Also detail the expected operational costs (e.g. licence, maintenance, human resources, updates to in-house developments) to estimate the total cost of ownership. Include real costs, otherwise describe them as a percentage of the total budget.  What recommendations can be drawn from the lessons learned? |
|  | 17 |  |  |
|  | 18 | Unintended consequences  (NM^c^) | Describe unintended consequences (positive or negative), harms or negative side-effects (if any). |
| Discussion | 19 | Conclusion  (M) | Summary of the conclusions and future implications. |
| General | 20 | General  (NM) | If applicable, include statement(s) on regulatory approvals (including, as appropriate, ethical approval, governance approval), trial or study registration (availability of protocol), and conflicts of interest. For implementation reports with a research component, ethical approval or a waiver from an appropriate ethics committee is required. For those without a research component, ethical considerations may still be relevant, but do not necessarily require approval or waiver. Authors may consult Eccles et al [2] for further guidance on ethical considerations in their specific context" |

^a^M: mandatory item.

^b^Stages adapted from the *WHO Digital Health Atlas*.

^c^NM: nonmandatory item.

## TIDieR-Telehealth

**Checklist Name:** TIDieR-telehealth: precision in reporting of telehealth interventions used in clinical trials – unique considerations for the Template for the Intervention Description and Replication (TIDieR)checklist

**Link to Equator-Network Entry:** https://www.equator-network.org/reporting-guidelines/tidier-telehealth-precision-in-reporting-of-telehealth-interventions-used-in-clinical-trials-unique-considerations-for-the-template-for-the-intervention-description-and-replication-tidier-checkli/

**Version and (Date Last Updated):** 1 (June 6, 2022)

**Checklist:**

| Original Item | Additional Considerations for Telehealth Interventions | Examples (Actual and Hypothetical) |
| --- | --- | --- |
| BRIEF NAME | | |
| 1. Provide the name or a phrase that describes the intervention | Should include the word “telehealth” (or a term that very clearly indicates that an intervention is being delivered remotely - e.g., “telemedicine, remote digital health, telephone”) in the brief name that describes the intervention. | Telephone Coaching to Enhance a Home-Based Physical Activity Program for Knee Osteoarthritis: A Randomized Clinical Trial [3].  Telehealth Versus In-Person Acceptance and Commitment Therapy for Chronic Pain: A Randomized Noninferiority Trial [4]. |
| WHY | | |
| 2. Describe any rationale, theory, or goal of the elements essential to the intervention | Provide the rationale for using a telehealth intervention. Is remote delivery used to expand access, or to enhance safety for participants? Is remote delivery an evidence-based option for the intervention? Is the goal to validate use of a traditional in-person intervention delivered in a remote format? | “Stigma and geographic barriers often prevent rural veterans from engaging in these evidence-based treatments. A large portion (37.7%) of VHA [Veterans Health Administration] enrollees diagnosed with PTSD live in rural areas. The objective of this pragmatic effectiveness trial was to test a collaborative care model designed to improve access to and engagement in evidence-based psychotherapy and pharmacotherapy for rural veterans [5].”  “A 12-week standard smoking cessation program is available in Japan; however, it requires face-to-face clinic visits, which has been one of the key obstacles to completing the program, leading to a low smoking cessation success rate. Telemedicine using internet-based video counseling instead of regular clinic visits could address this obstacle [6].” |
| WHAT | | |
| 3. Materials: Describe any physical or informational materials used in the intervention, including those provided to participants or used in intervention delivery or in the training of intervention providers. Provide information on where the materials can be accessed (e.g. online appendix, URL). | What components does the telehealth intervention include (e.g., audio, video, name of platform or software)? What (if any) additional documentation, instruction and/or equipment was provided (loaned or given) to participants? Were participants that did not have access to the equipment or platform excluded? | “digital study materials (training protocol and video, PsychoPy experiment files and stimuli; (link), and analysis scripts (link) are publicly available in an Open Science Framework repository [7].”  “At the end of the training sessions participants received a pair of prism goggles in a sealed opaque bag, a pointing sheet, written instructions, and a link to a video tutorial to take home [7].”  “The details of the telemedicine system myIBDcoach have been described elsewhere (cite)(link). MyIBDcoach is a secured web page with an HTML application for tablet or smartphone. Exclusion criteria were an inability to read or understand the informed consent form, and lack of internet access by computer, tablet, or smartphone [8].”  “Video counseling was delivered via Polycom PVX, a program installed on desktop computers and linked to the University study staff via the Internet. Each participating site received a desktop computer, webcam, and Polycom PVX software. A study technician installed equipment, tested connections with the site delivering the intervention, and trained clinic staff in equipment use and troubleshooting. The technician placed a binder with connection checklists, troubleshooting tips, and emergency phone numbers next to the study equipment. The technician also met with Internet service managers at each site to set up lines of communication for problem-solving connection issues that might arise throughout the trial [9].” |
| 4. Procedures: Describe each of the procedures, activities, and/or processes used in the intervention, including any enabling or support activities. | Were the procedures for this intervention originally developed for in-person or remote delivery (number of minutes, open accessibility or requires sign-up/set-up)? What, if anything, was done to adapt procedures from in-person delivery? | “The study protocol consisted of a manualized, 8-week ACT for chronic pain intervention (see Intervention section) used in previous research (for non-virtual delivery). The in-person and virtual versions of Acceptance and Commitment Therapy (ACT) used a treatment protocol (manual available upon request) that was previously used in a randomized controlled trial (in person) comparing ACT with cognitive and behavior therapy for chronic pain (citation) modified for individual rather than group administration [4].” |
| WHO PROVIDED | | |
| 5. For each category of intervention provider (e.g. psychologist, nursing assistant), describe their expertise, background and any specific training given. | Who delivered the telehealth intervention? Was there any training that went into the delivery of the intervention? Who all was authorized/approved to deliver it and how did they achieve authorization approval (e.g., training, certification process)? | “The off-site telepsychologists delivered 12 sessions of individual CPT (veteran/military version) to interested patients. In addition to monitoring PTSD symptoms for the telepsychologist, the nurse care manager encouraged CPT initiation, attendance, and homework adherence. The off-site telepsychiatrist educated CBOC providers, supervised the TOP care team, and conducted interactive video psychiatric consultations as necessary [5].”  “Once equipment was installed, the study project director conducted clinic staff training with each site via the Polycom system, in order to reinforce skills and build confidence in using the system. During this meeting, the project director reviewed study materials with the clinic staff, focusing on the clinic role in care such as reviewing prescription requests and providing medication prescriptions, as outlined below [9].”  “participants were trained in person in how to carry out the treatment by a research psychologist [7].”  “Study therapists were required to have graduate (at least master’s level) training in psychology. To avoid confounding the effects of mode of treatment with allegiance effects and therapist skill in nonspecific elements of therapy, study therapists conducted in-person as well as virtual delivery of treatment [4].” |
| HOW | | |
| 6. Describe the modes of delivery (e.g., face-to-face or by some other mechanism, such as internet or telephone) of the intervention and whether it was provided individually or in a group. | Indicate whether the intervention was delivered solely through remote methods or in a hybrid (remote + in-person) format. Synchronous versus asynchronous, unidirectional or bidirectional (could the participant/attendee ask questions, respond, interact and if so how - voice, chat, etc.?) | “Immediately after Research Session #2, participants were trained in person in how to carry out the treatment by a research psychologist according to a standardised protocol (available in study materials). Once the researcher was satisfied that the participant understood the treatment procedure, they performed the first treatment during this training session under the guidance of the researcher. After the training session, participants were instructed to perform twice-daily self-guided treatment sessions at home for two weeks [7].” |
| WHERE | | |
| 7. Describe the type(s) of location(s) where the intervention occurred, including any necessary infrastructure or relevant features. | Were clinicians in the clinic or at their home? Were patients in the clinic, another remote clinic, or at home? | “Because most computers were located in dedicated rooms in study clinics, participants could sign in at the clinic reception and go directly to the ... room for their session [9].”  Three off-site PTSD care teams were located at the Veterans Affairs Medical Center (VAMC). Care manager and pharmacist activities were conducted by telephone (to the patient’s home). Psychotherapy and psychiatric consultations were delivered via interactive video (to the community-based outpatient clinic, from the VAMC). All feedback and treatment recommendations (from PTSD care team) were given to CBOC [Community Based Outpatient Clinic] providers via the electronic health record with requests for additional signatures when clinical action was needed [5].  Both interventions were home based. Both groups received a motivational app and remote supervision at home by a coach [10]. |
| WHEN and HOW MUCH | | |
| 8. Describe the number of times the intervention was delivered and over what period of time including the number of sessions, their schedule, and their duration, intensity or dose. | Provide the planned intervention dosing (visits, frequency, duration, etc.) for the trial (expected treatment to meet optimal fidelity) and then also the number of actual visits received. Provide duration and frequency of sessions. | “In addition to the treatment they underwent during training (in person), participants were instructed to perform twice-daily self-guided treatment sessions at home for two weeks, resulting in 29 treatment sessions in total. They were instructed to commence the home-based treatment on the day following Research Session 2, perform one session in the morning and one in the evening, and record the start and end time of each session in a provided logbook [7].” |
| TAILORING | | |
| 9. If the intervention was planned to be personalized, titrated or adapted, then describe what, why, when, and how. | Describe the flexibility of the intervention to allow for any changes in or tailoring of the telehealth intervention for specific patients or groups. | “the PCPs could access the dermatologists online asynchronously via consultation or request a dermatologist to assume care (based on preference). Patients randomized to the online group had the option of accessing dermatologists online asynchronously [11].”  Patients could choose their own device of preference (e.g., phone type, tablet, laptop computer) on which to receive the intervention. |
| MODIFICATIONS | | |
| 10. If the intervention was modified during the course of the study, describe the changes (what, why, when, and how). | If it was planned remotely or in-person and then had to be switched to the other, provide the timing, reasons, and rationale for the change. | The initial mindfulness intervention was planned for 8 sessions in person at the clinic. However, part-way through the trial (after treatment was completed for 87 [60%] patients), clinic closures resulted in a modification to the delivery, necessitating it to be delivered remotely for the remainder of the study. |
| HOW WELL | | |
| 11. Planned: If intervention adherence or fidelity was assessed, describe how and by whom, and if any strategies were used to maintain or improve fidelity, describe them. | Identify any specific strategies used to improve adherence to the telehealth intervention. Was there a plan to monitor and track fidelity of the intervention?? | “Therapist fidelity to Cognitive Processing Therapy (CPT) will be assessed via medical record review by dichotomously classifying each session as per protocol (ie, session 1, impact statement; sessions 2–7, stuck points; session 8, safety; session 9, trust; session 10, power/control; session 11, esteem and impact statement; and session 12, intimacy and impact statement). Overall CPT fidelity was defined as the percentage of sessions delivered per protocol [5].”  To ensure treatment protocol adherence and competence in delivering treatment, therapists received 1 hour of weekly group supervision co-led by the second and senior authors [4].  “Patients’ training adherence was defined as a percentage counted from the total number of accomplished training sessions of an individual participant. Patients in the ITG group recorded the training sessions in the Polar Flow web application using the wrist heart rate monitor [12].” |
| 12. Actual: If intervention adherence or fidelity was assessed, describe the extent to which the intervention was delivered as planned. | Did the telehealth intervention influence actual treatment adherence? Was the fidelity of the telehealth intervention reported? | “Approximately 20% (twenty-two) of the participants in the TELE group received, in addition to the telerehabilitation sessions, one or more face-to-face home visits (mean, 2.3 ± 2.2 visits). The documented reasons for visiting TELE group participants at home were a poor Internet connection or persisting technical problems (six visits), delayed technology installation (twelve visits), an abnormal profile of knee recovery (three visits), unavailability of clinicians (two visits), and anxiety of the participant (one visit). In addition, six participants did not receive the allocated intervention because of dissatisfaction with the result of randomization, a poor Internet connection, and a perception of a complete recovery [13].”  “Among TOP patients attending any CPT sessions, 505 of the 514 sessions (98.2%) were conducted via interactive video, and the mean fidelity score to the CPT protocol was 79.8% [5].” |

## Search Strategy

| **Search Date:** | Originally in May 2024 and repeated in January 2026 |
| --- | --- |
| **Filtering Performed on Database:** | Performed via filter controls on database, the date was adjusted to include all of time up-to May 2025. No other filters were applied. Papers were then manually screened based on stated inclusion/exclusion criteria. |
| **Search String Development** | This search string was originally developed as part of the first systematic review conduced in 2021. The search term strategy was originally developed and workshopped by two senior clinical specialists and researchers [BJP, SCR], and 3 early career researchers (1 clinical [SS] and 1 digital health [TV]), all of whom have received training in literature review methodologies. The searches then underwent several trials within the databases to formulate the below search strings. This was led by TV due to background in computer science and familiarity with Boolean logic and programming.  For this updated review, the search strings were again reviewed and were not modified. |
| **PUBMED SEARCH STRING:** | |
| ("Virtual Care" OR "Virtual Health" OR Virtual*[tiab] OR "Remote Monitoring" OR "Remote Patient Monitoring" OR Telemonitor*[tiab] OR "Digital Health" OR digital health[tiab] OR "Home Monitoring" OR home monitor*[tiab] OR eHealth OR e-health[tiab] OR Telehealth OR Tele-health[tiab] OR Telemedicine OR Tele-medicine[tiab] OR "Wearable Devices" OR wearable*[tiab] ) AND ("Cystic Fibrosis"[Mesh] OR "Cystic Fibrosis"[tiab]) | |
| **WEB OF SCIENCE:** | |
| TS=(Cystic Fibrosis AND Virtual Health) OR TS=(Cystic Fibrosis AND Remote Monitoring) OR TS=(Cystic Fibrosis AND TeleMonitoring) OR TS=(Cystic Fibrosis AND Digital Health) OR TS=(Cystic Fibrosis AND home Monitoring) OR TS=(Cystic Fibrosis AND ehealth) OR TS=(Cystic Fibrosis AND telehealth) OR TS=(Cystic Fibrosis AND telemedicine) OR TS=(Cystic Fibrosis AND wearable devices) OR TS=(Cystic Fibrosis AND Virtual Care) | |
| **SCOPUS:** | |
| ("Cystic Fibrosis" AND "Virtual Health") OR ("Cystic Fibrosis" AND "Remote Monitoring") OR ("Cystic Fibrosis" AND "TeleMonitoring") OR ("Cystic Fibrosis" AND "Digital Health") OR ("Cystic Fibrosis" AND "home Monitoring") OR ("Cystic Fibrosis" AND "ehealth") OR ("Cystic Fibrosis" AND "telehealth") OR ("Cystic Fibrosis" AND "telemedicine") OR ("Cystic Fibrosis" AND "wearable devices") OR ("Cystic Fibrosis" AND "Virtual Care") | |

## References

1. World Health Organization. Classification of Digital Health Interventions v 1.0: a shared language to describe the uses of digital technology for health. https://iris.who.int/server/api/core/bitstreams/5ed36c71-bc74-41e9-8751-ca8bd9a8d823/content
2. Eccles, MP, Weijer, C, Mittman, B. Requirements for ethics committee review for studies submitted to Implementation Science. *Implementat Sci* 2011; 6(1): 1-3.
3. Watanabe AH, Willis C, Ragsdale R, Biskupiak J, Moore K, Brixner D, Young D. Patient perspectives on the use of digital technology to help manage cystic fibrosis. *Pulm Med* 2023; 2023:5082499
4. Shanthikumar S, Ruseckaite R, Corda J, Mulrennan S, Ranganathan S, Douglas T. Telehealth use in Australian cystic fibrosis centers: clinician experiences. *Pediatr Pulmonol* 2023 Oct; 58(10):2906-2915
5. Dixon E, Dick K, Ollosson S, Jones D, Mattock H, Bentley S, et al. Telemedicine and cystic fibrosis: do we still need face-to-face clinics? *Paediatr Respir Rev* 2022; 42: 23-28.
6. Beaufils F, Enaud R, Gallode F, Boucher G, Macey J, Berger P, Fayon M, Bui S. Adherence, reliability, and variability of home spirometry telemonitoring in cystic fibrosis. *Front Pediatr* 2023; 11:1111088.
7. Collaco JM, Albon D, Ostrenga JS, Flume P, Schechter MS, Cromwell EA. Factors associated with receiving CF care and use of telehealth in 2020 among persons with cystic fibrosis in the United States. *J Cyst Fibros* 2023 May; 22(3):456-463.
8. Corinna Morlacchi L, Privitera E, Rossetti V, Santambrogio M, Bellofiore A, Rosso L, Palleschi A, Nosotti M, Blasi F. Telemonitoring: an opportunity in cystic fibrosis lung transplant recipients. *Heliyon* 2023 Oct; 9(10):e19931.
9. Edmondson C, Westrupp N, Short C, Seddon P, Olden C, Wallis C, Brodlie M, Baxter F, McCormick J, MacFarlane S, Brooker R, Connon M, Ghayyda S, Blaikie L, Thursfield R, Brown L, Price A, Fleischer E, Hughes D, Donnelly C, Rosenthal M, Wallenburg J, Brownlee K, Alton EWFW, Bush A, Davies JC. Unsupervised home spirometry is not equivalent to supervised clinic spirometry in children and young people with cystic fibrosis: results from the CLIMB-CF study. *Pediatr Pulmonol* 2023 Oct; 58(10):2871-2880.
10. Stalker HJ, Jonasson AR, Hopfer SM, Collins MS. Improvement in cystic fibrosis newborn screening program outcomes with genetic counseling via telemedicine. *Pediatr Pulmonol* 2023 Dec; 58(12):3478-3486.
11. Layton AM, Irwin AM, Mihalik EC, Fleisch E, Keating CL, DiMango EA, Shah L, Arcasoy SM. Telerehabilitation using fitness application in patients with severe cystic fibrosis awaiting lung transplant: a pilot study. *Int J Telemed Appl* 2021; 2021:6641853.
12. Morsa M, Perrin A, David V, Rault G, Le Roux E, Alberti C, Gagnayre R, Pougheon Bertrand D. Experiences among patients with cystic fibrosis in the mucoexocet study of using connected devices for the management of pulmonary exacerbations: grounded theory qualitative research. *JMIR Form Res* 2024 Jan 23; 8:e38064.
13. Poulsen M, Holland AE, Button B, Jones AW. Preferences and perspectives regarding telehealth exercise interventions for adults with cystic fibrosis: a qualitative study. *Pediatr Pulmonol* 2024 May; 59(5):1217-1226.
