# Supplementary material for: Reporting of Telehealth Implementation in Cystic Fibrosis: Scoping Review Using a Novel Theory-Based Evaluation Lens
Source: J Med Internet Res. 2026 May 22;28:e86194. doi: 10.2196/86194 (PMC13241801; doi:10.2196/86194)
Supplement: Multimedia Appendix 5 [file jmir_v28i1e86194_app5.docx]

## Disclosure of Delegation to Generative AI

The authors declare the use of generative AI in the research and writing process. According to the GAIDeT taxonomy (2025), the following tasks were delegated to GAI tools under full human supervision:

- Data collection

- Data curation and organization

- Data analysis

The GAI tool used was: Elicit Plus 2025-2026.

Responsibility for the final manuscript lies entirely with the authors.

GAI tools are not listed as authors and do not bear responsibility for the final outcomes.

Declaration submitted by: Tamara Vagg

Additional note: We use Elicit AI to extract data from the found manuscripts based on the predefined checklists. Elicit AI extracted data under specified structured answers to allow for scoring.

## Elicit AI Prompts

| **Label** | **Label Description** | **Response** |
| --- | --- | --- |
| **iCHECK** | | |
| Title | Identification as an implementation report, and description of the implementation in the title and/or keywords | Answer Structure = Specified.  Specified possible answers:   - Did not report - Partially Reported - Reported Completely |
| Abstract | Provide a summary of the key elements of the implementation report, including a description of the implementation strategy, the intervention, defining the key elements of the implementation and health outcomes and specify the key KPIs/Outputs. We recommend describing the main aspects of the research in the following order: Background - Objectives - Methods - Implementation (Results) - Conclusions - (Optional: Trial Registration). |  |
| Context | Describe the geographical areas, organizations, target populations and implementation context. Consider social, cultural, economic, political, health care and organizational barriers, infrastructures and facilitators that may influence implementation elsewhere. Explicitly highlight whether a national digital health strategy exists and whether implementation is aligned with the strategy.  Describe the stage of the implementation (Developing or Adapting Solution / Piloting and Evidence generation / Package and Advocacy /Acceleration / Deploying / Scaling up / Hand over or Complete). |  |
| Problem Statement | Description of the health care or public health problem, challenge, or deficiency that the implementation aims to address. (If applicable, include a reference to the 'health system challenge' of the WHO Classification of Digital Health Interventions^^[[1]](#footnote-1)^^ in the description) |  |
| Similar Intervention | Mention whether this implementation was inspired by another existing one, and if so, what is the added value of your intervention, if any, compared to the initial one? And what, if anything, has been done differently? |  |
| Aims & Objectives | Describe the main objectives and the overall aim of the implementation. Describe how these will be measured using predefined primary and secondary outcome(s) and key performance indicators for this implementation and the expected intervention(s).  *For example: indicators or proxy-indicators measuring direct health outcomes (e.g., HbA1c for diabetic patients); Key Performance Indicators (e.g., number of users, number of users that are properly trained, user satisfaction); Indicator assessing a particular process (e.g., administrative time for patient admission);*  (If there was no evaluation, provide detailed explanation for reasoning) |  |
| Blueprint Summary | Describe the design and key features of the intervention and key points of the implementation strategy and roadmap. |  |
| Technical Design | Reasons for developing or choosing this tool. Does it combine several tools? Provide a brief description of the tool(s) (functionality and architecture) and how it fits into the health enterprise architecture and investment roadmap (if applicable). Indicate whether the solution is based on an existing solution or has been developed or purchased specifically for this intervention.  Describe the type of technology used (e.g., AI applications), license of the technology (open source, free, commercial, IP ownership etc.), include code documentation (if available), link to the application, link to wiki or project website. |  |
| Target | The target refers to the focus or recipient of the intervention. It is the specific person, group, system, or problem that the intervention aims to change or improve. The characteristics of the targeted "site(s)" (locations, staff, resources, etc.) for implementation and any eligibility criteria. The population targeted by the intervention and any eligibility criteria. |  |
| Data | Describe the data governance, including life cycle (collection, processing, storage, modification, sharing, suppression), the data ownership (mention whether patients actually have access to the data), data protection measures, confidential use of routine data, expected level of data integration, data for research, cross-border data agreement, if any, the applicable legal framework, and how the project complies with it. Data consent: Has patient consent been obtained? Describe the approach to data protection and cybersecurity (e.g. security by design, privacy by design, etc.) and where the data is hosted. (e.g., in-country, cloud based, hybrid model etc.). Describe, if applicable, the government preferences in terms of data policies. |  |
| Interoperability | Describe the interfaces (what other systems does the tool connect to) and the standards that were used (which specific ones and rationale of choice) (e.g., semantic ontologies such ICD as SNOMED, LOINC or technical standards such as HL7 FHIR, etc.). |  |
| Participating Entities | Describe the implementing organization(s): Type of organisation(s), mission, leadership, vision, etc.  Government involvement: Describe whether the government was involved in the implementation, at what level and at what stage(s).  Partners: Describe all partners (organisations) and their role in the implementation.  Funders: List all actors and stakeholders who have funded or invested in the development of the implementation (if different from the implementation, e.g. using an existing digital health intervention). Indicate their level of involvement in terms of funding.  Mention which entity will own the final product and intellectual property after the implementation phase. |  |
| Budget Planning | Describe the planned budget for implementation (include costs such as change management, user training, project management, technology pricing, total cost of ownership). If possible, include actual costs, otherwise describe the range or percentage of the total budget. Indicate the period covered by the budget. Describe the budget for the intervention (e.g. development, purchase or adaptation of a free tool); if possible include real costs, otherwise describe as a percentage of the total budget. Indicate the duration covered by the budget. |  |
| Sustainability | Describe the Business model including the sustainability model (financial, economic, environment etc.). If possible, put outcomes in relation to cost to assess sustainability. Describe long term exit strategies, and all dimensions considered to sustain the project after the end of funding. If applicable, describe potential institutionalization of the project. |  |
| Coverage | Describe whether the coverage of implementation is international, national, regional or at the level of e.g. municipalities. If coverage is sub-national, describe the regions. Provide information on the relative importance of the coverage (e.g. % of eligible population covered). |  |
| Outcomes | Primary and other outcome(s) of the implementation. Detail the actual outcomes, using the pre-defined outcome measures (if applicable). |  |
| Lessons Learned | Describe any lessons learned from the implementation experience that could be used to improve future outcomes. This could include, but is not limited to, success factors, implementation challenges or budget considerations.  Success factors: Describe factors that positively influenced the implementation (e.g. involvement of key stakeholders). Also describe contextual factors that may have positively influenced the results (e.g. new legal requirements that facilitated adoption).  Challenges to implementation: Describe challenges (process-related, such as resistance to change, but also technical). Include contextual factors that may have affected the achievement of outcomes such as an unexpected change of government, or 'opposing key players' who, despite potential participation, may hinder implementation (e.g. software companies managing regional digital health may act as barriers to innovation).  Budget: Describe whether the implementation budget was adhered to, and if not, why not. Also detail the expected operational costs (e.g. licence, maintenance, human resources, updates to in-house developments) to estimate the total cost of ownership. Include real costs, otherwise describe them as a percentage of the total budget.  What recommendations can be drawn from the lessons learned? |  |
| Unintended Consequences | Describe unintended consequences (positive or negative), harms or negative side-effects (if any). |  |
| Conclusion | Summary of the conclusions and future implications. |  |
| Discussion | Summary of the conclusions and future implications. |  |
| General | If applicable, include statement(s) on regulatory approvals (including, as appropriate, ethical approval, governance approval), trial or study registration (availability of protocol), and conflicts of interest. For implementation reports with a research component, ethical approval or a waiver from an appropriate ethics committee is required. For those without a research component, ethical considerations may still be relevant, but do not necessarily require approval or waiver. Authors may consult [this article](https://implementationscience.biomedcentral.com/articles/10.1186/1748-5908-6-32) for further guidance on ethical considerations in their specific context" |  |
| **TIDiER** | | |
| Brief Name | Provide the name or a phrase that describes the intervention. Should include the word “telehealth” (or a term that very clearly indicates that an intervention is being delivered remotely - e.g., “telemedicine, remote digital health, telephone”) in the brief name that describes the intervention. | Answer Structure = Specified.  Specified possible answers:   - Did not report - Partially Reported - Reported Completely |
| Why | Describe any rationale, theory, or goal of the elements essential to the intervention. Provide the rationale for using a telehealth intervention. Is remote delivery used to expand access, or to enhance safety for participants? Is remote delivery an evidence-based option for the intervention? Is the goal to validate use of a traditional in-person intervention delivered in a remote format? |  |
| What 1 | Materials: Describe any physical or informational materials used in the intervention, including those provided to participants or used in intervention delivery or in the training of intervention providers. Provide information on where the materials can be accessed (e.g. online appendix, URL). What components does the telehealth intervention include (e.g., audio, video, name of platform or software)? What (if any) additional documentation, instruction and/or equipment was provided (loaned or given) to participants? Were participants that did not have access to the equipment or platform excluded? |  |
| What 2 | Procedures: Describe each of the procedures, activities, and/or processes used in the intervention, including any enabling or support activities. Were the procedures for this intervention originally developed for in-person or remote delivery (number of minutes, open accessibility or requires sign-up/set-up)? What, if anything, was done to adapt procedures from in-person delivery? |  |
| Who Provided | For each category of intervention provider (e.g. psychologist, nursing assistant), describe their expertise, background and any specific training given. Who delivered the telehealth intervention? Was there any training that went into the delivery of the intervention? Who all was authorized/approved to deliver it and how did they achieve authorization approval (e.g., training, certification process)? |  |
| How | Describe the modes of delivery (e.g., face-to-face or by some other mechanism, such as internet or telephone) of the intervention and whether it was provided individually or in a group. Indicate whether the intervention was delivered solely through remote methods or in a hybrid (remote + in-person) format. Synchronous versus asynchronous, unidirectional or bidirectional (could the participant/attendee ask questions, respond, interact and if so how - voice, chat, etc.?) |  |
| Where | Describe the type(s) of location(s) where the intervention occurred, including any necessary infrastructure or relevant features. Were clinicians in the clinic or at their home? Were patients in the clinic, another remote clinic, or at home? |  |
| When & How Much | Describe the number of times the intervention was delivered and over what period of time including the number of sessions, their schedule, and their duration, intensity or dose. Provide the planned intervention dosing (visits, frequency, duration, etc.) for the trial (expected treatment to meet optimal fidelity) and then also the number of actual visits received. Provide duration and frequency of sessions. |  |
| Tailoring | If the intervention was planned to be personalized, titrated or adapted, then describe what, why, when, and how. Describe the flexibility of the intervention to allow for any changes in or tailoring of the telehealth intervention for specific patients or groups. |  |
| Modifications | If the intervention was modified during the course of the study, describe the changes (what, why, when, and how). If it was planned remotely or in-person and then had to be switched to the other, provide the timing, reasons, and rationale for the change. |  |
| How Well 1 | Planned: If intervention adherence or fidelity was assessed, describe how and by whom, and if any strategies were used to maintain or improve fidelity, describe them. Identify any specific strategies used to improve adherence to the telehealth intervention. Was there a plan to monitor and track fidelity of the intervention? |  |
| How Well 2 | Actual: If intervention adherence or fidelity was assessed, describe the extent to which the intervention was delivered as planned. Did the telehealth intervention influence actual treatment adherence? Was the fidelity of the telehealth intervention reported? |  |

1. [↑](#footnote-ref-1)
