# Supplementary material for: Reporting of Telehealth Implementation in Cystic Fibrosis: Scoping Review Using a Novel Theory-Based Evaluation Lens
Source: J Med Internet Res. 2026 May 22;28:e86194. doi: 10.2196/86194 (PMC13241801; doi:10.2196/86194)
Supplement: Multimedia Appendix 6 [file jmir_v28i1e86194_app6.docx]

**Table S1.** Manual reviewer data extraction compared with Elicit AI extraction.

| iCHECK | | | | | | | | | | | | | | | | | | | | | | | | | | | | | | | | | | | | | | | | | | | | | | | | | | | | | | | | | | | |
| --- | --- | --- | --- | --- | --- | --- | --- | --- | --- | --- | --- | --- | --- | --- | --- | --- | --- | --- | --- | --- | --- | --- | --- | --- | --- | --- | --- | --- | --- | --- | --- | --- | --- | --- | --- | --- | --- | --- | --- | --- | --- | --- | --- | --- | --- | --- | --- | --- | --- | --- | --- | --- | --- | --- | --- | --- | --- | --- | --- |
| Study ID* |  | T | A | | | I | | | |  | | | |  | | | M | | |  | | |  | | | |  | | |  |  | | | |  | | | |  |  | | | R | |  | | |  | |  | | | | | | D | | | G |
|  |  | T | A | | | C | | | | P | | | | S | | | AO | | | BS | | | TD | | | | T | | | D | I | | | | PE | | | | BP | S | | | C | | O | | | LL | | UQ | | | | | | C | | | G |
| 1 | TV | 0 | | 1 | | | | 1 | | | 2 | | 2 | | | 2 | | | 1 | | | 1 | | | 2 | | | | 1 | | | | 0 | | | 2 | | 0 | | 1 | | | | 2 | | | 2 | | 2 | | | 1 | | 2 | | | | 1 | |
|  | Elicit | 2 | | 2 | | | | 1 | | | 2 | | 2 | | | 1 | | | 2 | | | 1 | | | 2 | | | | 1 | | | | 0 | | | 1 | | 0 | | 1 | | | | 1 | | | 2 | | 1 | | | 1 | | 2 | | | | 1 | |
| 2 | TV | 0 | | 1 | | | | 2 | | | 2 | | 2 | | | 2 | | | 1 | | | 1 | | | 2 | | | | 1 | | | | 0 | | | 2 | | 0 | | 0 | | | | 2 | | | 2 | | 2 | | | 1 | | 2 | | | | 1 | |
|  | Elicit | 1 | | 2 | | | | 1 | | | 2 | | 1 | | | 2 | | | 2 | | | 0 | | | 2 | | | | 1 | | | | 0 | | | 1 | | 0 | | 0 | | | | 1 | | | 2 | | 1 | | | 0 | | 2 | | | | 1 | |
| 3 | TV | 0 | | 1 | | | | 1 | | | 2 | | 2 | | | 2 | | | 0 | | | 1 | | | 2 | | | | 1 | | | | 0 | | | 1 | | 0 | | 1 | | | | 2 | | | 2 | | 2 | | | 2 | | 2 | | | | 1 | |
|  | Elicit | 1 | | 2 | | | | 1 | | | 2 | | 1 | | | 1 | | | 1 | | | 1 | | | 2 | | | | 1 | | | | 0 | | | 1 | | 0 | | 1 | | | | 1 | | | 2 | | 1 | | | 1 | | 2 | | | | 1 | |
| 4 | TV | 0 | | 1 | | | | 1 | | | 2 | | 2 | | | 2 | | | 1 | | | 1 | | | 2 | | | | 0 | | | | 0 | | | 1 | | 0 | | 1 | | | | 2 | | | 2 | | 2 | | | 0 | | 2 | | | | 0 | |
|  | Elicit | 2 | | 2 | | | | 1 | | | 1 | | 1 | | | 1 | | | 1 | | | 1 | | | 2 | | | | 0 | | | | 1 | | | 1 | | 0 | | 1 | | | | 1 | | | 0 | | 1 | | | 0 | | 1 | | | | 0 | |
| 5 | TV | 0 | | 1 | | | | 1 | | | 2 | | 2 | | | 2 | | | 2 | | | 1 | | | 2 | | | | 0 | | | | 0 | | | 1 | | 0 | | 1 | | | | 2 | | | 2 | | 2 | | | 2 | | 2 | | | | 2 | |
|  | Elicit | 2 | | 2 | | | | 1 | | | 2 | | 1 | | | 2 | | | 1 | | | 1 | | | 2 | | | | 1 | | | | 0 | | | 0 | | 0 | | 1 | | | | 1 | | | 2 | | 1 | | | 0 | | 2 | | | | 1 | |
| 6 | TV | 0 | | 1 | | | | 1 | | | 2 | | 2 | | | 2 | | | 2 | | | 1 | | | 2 | | | | 0 | | | | 1 | | | 0 | | 1 | | 0 | | | | 2 | | | 2 | | 2 | | | 2 | | 2 | | | | 1 | |
|  | Elicit | 1 | | 2 | | | | 1 | | | 1 | | 1 | | | 2 | | | 1 | | | 1 | | | 2 | | | | 0 | | | | 1 | | | 1 | | 1 | | 0 | | | | 1 | | | 2 | | 1 | | | 1 | | 2 | | | | 1 | |
| 7 | TV | 0 | | 1 | | | | 1 | | | 2 | | 2 | | | 2 | | | 2 | | | 0 | | | 2 | | | | 0 | | | | 0 | | | 0 | | 0 | | 0 | | | | 2 | | | 2 | | 2 | | | 2 | | 2 | | | | 1 | |
|  | Elicit | 1 | | 2 | | | | 1 | | | 2 | | 1 | | | 1 | | | 1 | | | 0 | | | 2 | | | | 0 | | | | 0 | | | 0 | | 0 | | 0 | | | | 0 | | | 2 | | 1 | | | 2 | | 2 | | | | 1 | |
| 8 | TV | 0 | | 1 | | | | 1 | | | 2 | | 1 | | | 2 | | | 2 | | | 1 | | | 2 | | | | 1 | | | | 0 | | | 1 | | 0 | | 0 | | | | 2 | | | 2 | | 1 | | | 0 | | 2 | | | | 2 | |
|  | Elicit | 1 | | 2 | | | | 1 | | | 1 | | 1 | | | 0 | | | 0 | | | 0 | | | 1 | | | | 1 | | | | 0 | | | 1 | | 0 | | 1 | | | | 1 | | | 2 | | 1 | | | 1 | | 2 | | | | 2 | |
| 9 | TV | 2 | | 1 | | | | 1 | | | 2 | | 1 | | | 2 | | | 1 | | | 0 | | | 2 | | | | 0 | | | | 0 | | | 2 | | 0 | | 1 | | | | 2 | | | 2 | | 2 | | | 2 | | 2 | | | | 0 | |
|  | Elicit | 2 | | 2 | | | | 1 | | | 2 | | 1 | | | 0 | | | 0 | | | 1 | | | 2 | | | | 0 | | | | 0 | | | 1 | | 0 | | 1 | | | | 1 | | | 1 | | 1 | | | 1 | | 2 | | | | 0 | |
| 10 | TV | 0 | | 1 | | | | 1 | | | 2 | | 2 | | | 2 | | | 1 | | | 1 | | | 2 | | | | 0 | | | | 0 | | | 2 | | 0 | | 1 | | | | 2 | | | 2 | | 2 | | | 2 | | 2 | | | | 2 | |
|  | Elicit | 1 | | 2 | | | | 1 | | | 1 | | 1 | | | 1 | | | 1 | | | 1 | | | 2 | | | | 0 | | | | 0 | | | 1 | | 0 | | 1 | | | | 2 | | | 1 | | 1 | | | 1 | | 2 | | | | 1 | |
| 11 | TV | 0 | | 1 | | | | 1 | | | 2 | | 1 | | | 2 | | | 2 | | | 1 | | | 2 | | | | 0 | | | | 0 | | | 2 | | 0 | | 0 | | | | 2 | | | 2 | | 1 | | | 0 | | 2 | | | | 2 | |
|  | Elicit | 2 | | 2 | | | | 1 | | | 1 | | 1 | | | 1 | | | 1 | | | 0 | | | 2 | | | | 1 | | | | 0 | | | 1 | | 0 | | 0 | | | | 1 | | | 2 | | 1 | | | 0 | | 2 | | | | 1 | |
| 73 | RD | 1 | | 1 | | | | 2 | | | 2 | | 2 | | | 2 | | | 1 | | | 1 | | | 2 | | | | 1 | | | | 1 | | | 1 | | 0 | | 0 | | | | 2 | | | 1 | | 1 | | | 1 | | 2 | | | |  | |
|  | Elicit | 2 | | 2 | | | | 1 | | | 2 | | 1 | | | 2 | | | 2 | | | 1 | | | 2 | | | | 1 | | | | 1 | | | 1 | | 0 | | 0 | | | | 1 | | | 2 | | 1 | | | 0 | | 2 | | | |  | |
| 74 | RD | 1 | | 1 | | | | 2 | | | 2 | | 0 | | | 1 | | | 2 | | | 1 | | | 1 | | | | 1 | | | | 0 | | | 1 | | 0 | | 1 | | | | 2 | | | 2 | | 1 | | | 1 | | 2 | | | |  | |
|  | Elicit | 2 | | 2 | | | | 1 | | | 2 | | 1 | | | 1 | | | 2 | | | 1 | | | 2 | | | | 1 | | | | 0 | | | 1 | | 0 | | 1 | | | | 1 | | | 2 | | 1 | | | 1 | | 2 | | | |  | |
| 75 | RD | 0 | | 2 | | | | 1 | | | 1 | | 0 | | | 1 | | | 1 | | | 0 | | | 2 | | | | 0 | | | | 0 | | | 0 | | 0 | | 0 | | | | 2 | | | 1 | | 1 | | | 1 | | 2 | | | |  | |
|  | Elicit | 2 | | 0 | | | | 1 | | | 2 | | 1 | | | 1 | | | 1 | | | 1 | | | 2 | | | | 1 | | | | 0 | | | 1 | | 0 | | 1 | | | | 1 | | | 2 | | 1 | | | 1 | | 2 | | | |  | |
| 76 | RD | 1 | | 1 | | | | 1 | | | 2 | | 1 | | | 2 | | | 2 | | | 2 | | | 2 | | | | 1 | | | | 1 | | | 1 | | 0 | | 0 | | | | 2 | | | 2 | | 1 | | | 1 | | 2 | | | |  | |
|  | Elicit | 2 | | 2 | | | | 1 | | | 2 | | 1 | | | 2 | | | 2 | | | 1 | | | 2 | | | | 1 | | | | 1 | | | 1 | | 0 | | 1 | | | | 1 | | | 2 | | 1 | | | 1 | | 2 | | | |  | |
| 78 | RD | 2 | | 0 | | | | 1 | | | 2 | | 1 | | | 2 | | | 2 | | | 1 | | | 2 | | | | 0 | | | | 0 | | | 2 | | 1 | | 1 | | | | 2 | | | 2 | | 1 | | | 1 | | 2 | | | |  | |
|  | Elicit | 2 | | 2 | | | | 1 | | | 2 | | 1 | | | 2 | | | 2 | | | 1 | | | 2 | | | | 0 | | | | 1 | | | 1 | | 1 | | 1 | | | | 1 | | | 2 | | 1 | | | 2 | | 2 | | | |  | |
| 80 | RD | 1 | | 2 | | | | 1 | | | 2 | | 1 | | | 2 | | | 2 | | | 1 | | | 2 | | | | 0 | | | | 1 | | | 1 | | 1 | | 1 | | | | 1 | | | 2 | | 1 | | | 1 | | 2 | | | |  | |
|  | Elicit | 2 | | 2 | | | | 1 | | | 2 | | 2 | | | 2 | | | 1 | | | 1 | | | 2 | | | | 0 | | | | 0 | | | 1 | | 0 | | 1 | | | | 1 | | | 2 | | 1 | | | 1 | | 2 | | | |  | |
| 81 | RD | 2 | | 0 | | | | 1 | | | 2 | | 2 | | | 2 | | | 2 | | | 1 | | | 2 | | | | 0 | | | | 0 | | | 1 | | 0 | | 1 | | | | 1 | | | 2 | | 1 | | | 1 | | 2 | | | |  | |
|  | Elicit | 2 | | 2 | | | | 1 | | | 1 | | 1 | | | 2 | | | 2 | | | 1 | | | 2 | | | | 0 | | | | 0 | | | 1 | | 0 | | 0 | | | | 1 | | | 2 | | 1 | | | 1 | | 2 | | | |  | |
| TiDiER | | | | | | | | | | | | | | | | | | | | | | | | | | | | | | | | | | | | | | | | | | | | | | | | | | | | | | | | | | | |
|  |  | BN | | | | | Wy | | What | | | | | | | | | WP | | | | | | How | | | | Where | | | | | | When & HM | | | | | | | Tailoring | | | | | Mods | | | | | | | How Well | | | | | | |
|  |  |  | | | | |  | | W1 | | | | | | W2 | | |  | | | | | |  | | | |  | | | | | |  | | | | | | |  | | | | |  | | | | | | | H1 | | | | H2 | | |
| 1 | TV | 0 | | | 2 | | | | | | | 1 | | | | 0 | | | | | 1 | | | | | 2 | | | | | | 2 | | | | | 1 | | | | | 2 | | | | | 2 | | | | 1 | | | | 0 | | | | |
|  | Elicit | 2 | | | 2 | | | | | | | 1 | | | | 0 | | | | | 1 | | | | | 2 | | | | | | 2 | | | | | 1 | | | | | 2 | | | | | 2 | | | | 1 | | | | 0 | | | | |
| 2 | TV | 2 | | | 2 | | | | | | | 1 | | | | 2 | | | | | 1 | | | | | 2 | | | | | | 2 | | | | | 2 | | | | | 2 | | | | | 0 | | | | 1 | | | | 1 | | | | |
|  | Elicit | 2 | | | 2 | | | | | | | 1 | | | | 2 | | | | | 1 | | | | | 2 | | | | | | 2 | | | | | 2 | | | | | 2 | | | | | 0 | | | | 1 | | | | 1 | | | | |
| 3 | TV | 2 | | | 2 | | | | | | | 2 | | | | 2 | | | | | 0 | | | | | 2 | | | | | | 1 | | | | | 1 | | | | | 0 | | | | | 0 | | | | 0 | | | | 1 | | | | |
|  | Elicit | 0 | | | 2 | | | | | | | 2 | | | | 2 | | | | | 0 | | | | | 2 | | | | | | 1 | | | | | 1 | | | | | 0 | | | | | 0 | | | | 0 | | | | 1 | | | | |
| 4 | TV | 2 | | | 2 | | | | | | | 1 | | | | 2 | | | | | 0 | | | | | 2 | | | | | | 2 | | | | | 0 | | | | | 1 | | | | | 0 | | | | 1 | | | | 1 | | | | |
|  | Elicit | 2 | | | 2 | | | | | | | 1 | | | | 2 | | | | | 0 | | | | | 2 | | | | | | 2 | | | | | 0 | | | | | 1 | | | | | 1 | | | | 1 | | | | 1 | | | | |
| 5 | TV | 2 | | | 2 | | | | | | | 1 | | | | 2 | | | | | 0 | | | | | 2 | | | | | | 2 | | | | | 1 | | | | | 0 | | | | | 0 | | | | 1 | | | | 1 | | | | |
|  | Elicit | 0 | | | 2 | | | | | | | 1 | | | | 2 | | | | | 0 | | | | | 2 | | | | | | 2 | | | | | 1 | | | | | 0 | | | | | 0 | | | | 1 | | | | 1 | | | | |
| 6 | TV | 2 | | | 2 | | | | | | | 1 | | | | 2 | | | | | 1 | | | | | 1 | | | | | | 1 | | | | | 1 | | | | | 1 | | | | | 0 | | | | 1 | | | | 2 | | | | |
|  | Elicit | 0 | | | 2 | | | | | | | 1 | | | | 2 | | | | | 1 | | | | | 1 | | | | | | 1 | | | | | 1 | | | | | 1 | | | | | 0 | | | | 1 | | | | 2 | | | | |
| 7 | TV | 1 | | | 2 | | | | | | | 0 | | | | 2 | | | | | 0 | | | | | 2 | | | | | | 1 | | | | | 1 | | | | | 1 | | | | | 2 | | | | 1 | | | | 2 | | | | |
|  | Elicit | 0 | | | 2 | | | | | | | 0 | | | | 2 | | | | | 0 | | | | | 2 | | | | | | 1 | | | | | 1 | | | | | 1 | | | | | 2 | | | | 1 | | | | 2 | | | | |
| 8 | TV | 2 | | | 2 | | | | | | | 1 | | | | 2 | | | | | 0 | | | | | 2 | | | | | | 1 | | | | | 0 | | | | | 0 | | | | | 0 | | | | 0 | | | | 0 | | | | |
|  | Elicit | 0 | | | 1 | | | | | | | 1 | | | | 2 | | | | | 0 | | | | | 2 | | | | | | 1 | | | | | 0 | | | | | 0 | | | | | 0 | | | | 0 | | | | 0 | | | | |
| 9 | TV | 2 | | | 2 | | | | | | | 1 | | | | 2 | | | | | 0 | | | | | 2 | | | | | | 1 | | | | | 1 | | | | | 1 | | | | | 0 | | | | 1 | | | | 0 | | | | |
|  | Elicit | 0 | | | 2 | | | | | | | 1 | | | | 2 | | | | | 0 | | | | | 2 | | | | | | 1 | | | | | 1 | | | | | 1 | | | | | 0 | | | | 1 | | | | 0 | | | | |
| 10 | TV | 2 | | | 2 | | | | | | | 1 | | | | 2 | | | | | 0 | | | | | 1 | | | | | | 2 | | | | | 1 | | | | | 0 | | | | | 1 | | | | 1 | | | | 1 | | | | |
|  | Elicit | 0 | | | 2 | | | | | | | 1 | | | | 2 | | | | | 0 | | | | | 1 | | | | | | 1 | | | | | 1 | | | | | 0 | | | | | 1 | | | | 1 | | | | 1 | | | | |
| 11 | TV | 2 | | | 2 | | | | | | | 1 | | | | 2 | | | | | 1 | | | | | 2 | | | | | | 2 | | | | | 1 | | | | | 0 | | | | | 0 | | | | 1 | | | | 2 | | | | |
|  | Elicit | 0 | | | 2 | | | | | | | 1 | | | | 2 | | | | | 1 | | | | | 2 | | | | | | 2 | | | | | 1 | | | | | 0 | | | | | 0 | | | | 1 | | | | 2 | | | | |
| 73 | RD | 2 | | | 1 | | | | | | | 1 | | | | 1 | | | | | 1 | | | | | 2 | | | | | | 2 | | | | | 2 | | | | | 2 | | | | | 0 | | | | 1 | | | | 1 | | | | |
|  | Elicit | 2 | | | 2 | | | | | | | 1 | | | | 2 | | | | | 0 | | | | | 2 | | | | | | 2 | | | | | 1 | | | | | 1 | | | | | 0 | | | | 1 | | | | 1 | | | | |
| 74 | RD | 2 | | | 2 | | | | | | | 2 | | | | 2 | | | | | 0 | | | | | 2 | | | | | | 1 | | | | | 1 | | | | | 0 | | | | | 0 | | | | 0 | | | | 1 | | | | |
|  | Elicit | 0 | | | 2 | | | | | | | 2 | | | | 2 | | | | | 0 | | | | | 2 | | | | | | 1 | | | | | 1 | | | | | 0 | | | | | 0 | | | | 0 | | | | 1 | | | | |
| 75 | RD | 2 | | | 2 | | | | | | | 1 | | | | 2 | | | | | 1 | | | | | 1 | | | | | | 1 | | | | | 1 | | | | | 1 | | | | | 0 | | | | 0 | | | | 0 | | | | |
|  | Elicit | 2 | | | 2 | | | | | | | 1 | | | | 2 | | | | | 0 | | | | | 2 | | | | | | 1 | | | | | 1 | | | | | 1 | | | | | 0 | | | | 0 | | | | 1 | | | | |
| 76 | RD | 2 | | | 2 | | | | | | | 1 | | | | 2 | | | | | 2 | | | | | 2 | | | | | | 1 | | | | | 2 | | | | | 2 | | | | | 2 | | | | 2 | | | | 2 | | | | |
|  | Elicit | 0 | | | 2 | | | | | | | 2 | | | | 2 | | | | | 1 | | | | | 2 | | | | | | 2 | | | | | 1 | | | | | 1 | | | | | 2 | | | | 1 | | | | 1 | | | | |
| 78 | RD | 2 | | | 2 | | | | | | | 1 | | | | 2 | | | | | 1 | | | | | 1 | | | | | | 1 | | | | | 1 | | | | | 1 | | | | | 0 | | | | 1 | | | | 2 | | | | |
|  | Elicit | 0 | | | 2 | | | | | | | 1 | | | | 2 | | | | | 1 | | | | | 1 | | | | | | 1 | | | | | 1 | | | | | 1 | | | | | 0 | | | | 1 | | | | 2 | | | | |
| 80 | RD | 2 | | | 2 | | | | | | | 2 | | | | 2 | | | | | 1 | | | | | 2 | | | | | | 2 | | | | | 2 | | | | | 2 | | | | | 0 | | | | 2 | | | | 1 | | | | |
|  | Elicit | 0 | | | 2 | | | | | | | 2 | | | | 1 | | | | | 1 | | | | | 2 | | | | | | 2 | | | | | 2 | | | | | 2 | | | | | 1 | | | | 2 | | | | 2 | | | | |
| 81 | RD | 2 | | | 2 | | | | | | | 2 | | | | 2 | | | | | 1 | | | | | 2 | | | | | | 2 | | | | | 2 | | | | | 2 | | | | | 1 | | | | 1 | | | | 1 | | | | |
|  | Elicit | 2 | | | 2 | | | | | | | 1 | | | | 2 | | | | | 1 | | | | | 2 | | | | | | 1 | | | | | 2 | | | | | 2 | | | | | 1 | | | | 1 | | | | 2 | | | | |

*See Table S2 for ID full reference TV + RD = Reviewer Initials.

**Table S2.** Summary of studies [1-98] for the review.

| Study ID | Title | Authors | Journal | Year |
| --- | --- | --- | --- | --- |
| 1 | Development and evaluation of an internet-based cognitive behavioral therapy intervention for anxiety and depression in adults with cystic fibrosis (eHealth CF-CBT): An international collaboration | Marieke Verkleij, Anna M Georgiopoulos, Deborah Friedman [1] | Internet Interventions | 2021 |
| 2 | Psychological interventions during COVID pandemic: Telehealth for individuals with cystic fibrosis and caregivers | Sonia Graziano, Francesca Boldrini, Dario Righelli, Francesco Milo, \| Vincenzina, Lucidi, Alexandra Quittner, Paola Tabarini [2] | Pediatric Pulmonology | 2021 |
| 3 | Patient and family perceptions of telehealth as part of the cystic fibrosis care model during COVID-19 | J Davis, A Nesmith, R Perkins, J Bailey, C Siracusa, N Chaudary, M Powers, G S Sawicki, G M Solomon [3] | Journal of Cystic Fibrosis | 2021 |
| 4 | Managing Cystic Fibrosis related diabetes via telehealth during COVID-19 pandemic | Sana Hasan, M Cecilia Lansang, Mohammad Salman Khan, Elliot Dasenbrook [4] | Journal of Clinical & Translational Endocrinology | 2021 |
| 5 | Physiotherapy via telehealth for acute respiratory exacerbations in paediatric cystic fibrosis | Cloe Benz, Anna Middleton, Alison Elliott, Adrienne Harvey [5] | Journal of Telemedicine and Telecare | 2021 |
| 6 | Health care costs related to home spirometry in the eICE randomized trial | Natalie Franz, Hannah Rapp, Ryan N Hansen, Laura S Gold, Christopher H Goss, Noah Lechtzin, Larry G Kessler [6] | Journal of Cystic Fibrosis | 2021 |
| 7 | Change in CF care during COVID-19 pandemic: Single-center experience in a middle-income setting | Elpis Hatziagorou, \| Ilektra, Vasiliki Avramidou, Asterios Kampouras, \| Venetia Tsara, John Tsanakas [7] | Pediatric Pulmonology | 2021 |
| 8 | Transitioning to telehealth during the coronavirus disease 2019 pandemic: Perspectives from partners of women with cystic fibrosis and healthcare providers | Jessica Corcoran, Caitlin Marley Campbell, Sigrid Ladores [8] | Chronic Illness | 2021 |
| 9 | Barriers and facilitators to implementing telehealth services during the COVID-19 pandemic: A qualitative analysis of interviews with cystic fibrosis care team members R | Aricca D Van Citters, Olivia Dieni, Peter Scalia, Christopher Dowd, Kathryn A Sabadosa, Jill D Fliege, Manu Jain, Robert W Miller, Clement L Ren [9] | Journal of Cystic Fibrosis | 2021 |
| 10 | Remote monitoring in telehealth care delivery across the U.S. cystic fibrosis care network | R Thida Ong, Aricca D Van Citters, Christopher Dowd, Jason Fullmer, Rhonda List, Shine-Ann Pai, Clement L Ren, Peter Scalia, George M Solomon, Gregory S Sawicki [10] | Journal of Cystic Fibrosis | 2021 |
| 11 | The Role of Telehealth Services in Children with Cystic Fibrosis During Coronavirus Disease 2019 Outbreak | O ¨mer, Faruk Bes Xer, Ebru Pelin, Ug ˘ur, Karabog ˘a, Evrim Hepkaya, Ays Xe Ayzıt, Kılınc ¸sakallı, Ahsen Do, Tu ¨nmez, ¨rkmen, Tug ˘c ¸e, Damla Dilek, Haluk C Xokug ˘ras, Fu ¨gen, C Xullu, C Xokug ˘ras [11] | Telemedicine journal and e-health | 2021 |
| 12 | Patient and Provider Experience With Cystic Fibrosis Telemedicine Clinic | Kelvin D Macdonald, Elizabeth R Gibb, Kalen Hendra, Fatima Neemuchwala, Marilynn Chan, Ngoc P Ly [12] | Frontiers in Pediatrics | 2021 |
| 13 | Patient and family experience of telehealth care delivery as part of the CF chronic care model early in the COVID-19 pandemic R | George M Solomon, Julianna Bailey, James Lawlor, Peter Scalia, Gregory S Sawicki, Christopher Dowd, Kathryn A Sabadosa, Aricca Van Citters [13] | Journal of Cystic Fibrosis | 2021 |
| 14 | Design and methods for understanding the state of cystic fibrosis care amid the COVID-19 pandemic R | Christopher Dowd, Aricca D Van Citters, Olivia Dieni, Anne Willis, Leslie Powell, Kathryn A Sabadosa [14] | Journal of Cystic Fibrosis | 2021 |
| 15 | Satisfaction and Concerns with Telemedicine Endocrine Care of Patients with Cystic Fibrosis | Rahat Ahmed, Margaret Greenfield, Christopher P Morley, Marisa Desimone [15] | Telemedicine Reports | 2022 |
| 16 | The effect of telerehabilitation on quality of life, anxiety, and depression in children with cystic fibrosis and caregivers: A single-blind randomized trial | Özge Kenis-Coskun, \| Ahsen, N Aksoy, Eda N Kumaş, Aybike Yılmaz, Elifnur Güven, Hatice H Ayaz, \| Tuğçe Sözer, \| Almala, P Ergenekon, Evrim Karadağ-Saygı [16] | Pediatric Pulmonology | 2022 |
| 17 | Cystic fibrosis learning network telehealth innovation lab during the COVID-19 pandemic: a success QI story for interdisciplinary care and agenda setting | Dana Albon, Lacrecia Thomas, Lindsay Hoberg, Sophia Stamper, Lindsay Somerville, Prigi Varghese, Ella Balasa, Matthew Roman, Maria T Britto, Melanie Miner, Emily Gehring, Clifford Gammon, Raouf S Amin, Michael Seid, Mike Powers [17] | BMJ Open Quality | 2022 |
| 18 | Management of Cystic Fibrosis during COVID-19: Patient Reported Outcomes based remote follow-up among CF patients in Denmark -A feasibility study | Lotte Rodkjaer, Majbritt Jeppesen, Liv Schougaard, Lotte Rodkjaer [18] | Journal of Cystic Fibrosis | 2022 |
| 19 | Telemedicine of patients with cystic fibrosis during the COVID-19 pandemic Teleconsultas de pacientes com fibrose cística durante a pandemia de COVID-19 | Rafaella Lima Ferreira Costa, Rebeca Ferreira Costa, Christine Pereira Gonçalves, Renata Wrobel, Folescu Cohen, Nelbe Nesi Santana [19] | Revista Paulista de Pediatria | 2022 |
| 20 | A prospective randomised controlled mixed-methods pilot study of home monitoring in adults with cystic fibrosis | Edward F Nash, Jocelyn Choyce, Victoria Carrolan, Edwin Justice, Karen L Shaw, Alice Sitch, Hema Mistry, Joanna L Whitehouse [20] | Therapeutic Advances in Respiratory Disease | 2022 |
| 21 | Pilot RCT of a telehealth intervention to reduce symptoms of depression and anxiety in adults with cystic fibrosis | Christina J Bathgate, Kristin M Kilbourn, Nora H Murphy, Frederick S Wamboldt, Kristen E Holm [21] | Journal of Cystic Fibrosis | 2021 |
| 22 | Cystic Fibrosis Respiratory Microbiology Monitoring during a Global Pandemic: Lessons Learned from a Shift to Telehealth | AN Franciosi, PG Wilcox, BS Quon [22] | Annals of the American Thoracic Society | 2021 |
| 23 | Evaluation of Mobile Phone-based Tele-monitoring of Cystic Fibrosis Patients during the COVID-19 Pandemic: A 3-year Experience in Iran | Lida Fadaizadeh, Maryam Hassanzad, Ali Valinejadi, Seyed Mohammad, Jafar Taheri, Poopak Farnia, Nima Hassanzad, Hossein Ali Ghaffaripoor, Noushin Baghaei, Mahdieh Arian, Masoumeh Ansari, Zahra Daneshmandi, Elham Sadat, Sadati, Parisa Honarpisheh, Mahsa Rekabi, Leila Mohammadpour, Ali Akbar Velayati [23] | Biomedical and Biotechnology Research Journal | 2022 |
| 24 | Real-World Outcomes in Cystic Fibrosis Telemedicine Clinical Care in a Time of a Global Pandemic | Lindsay A L Somerville, Rhonda P List, Martina H Compton, Heather M Bruschwein, Deirdre Jennings, Marieke K Jones, Rachel K Murray, Elissa R Starheim, Katherine M Webb, Lucy S Gettle, Dana P Albon, A L Somerville [24] | Chest | 2021 |
| 25 | Clinical usefulness Initiating home spirometry for children during the COVID-19 pandemic -A practical guide | C H Richardson, N J Orr, S L Ollosson, S J Irving, I M Balfour-Lynn, S B Carr [25] | Paediatric Respiratory Reviews | 2022 |
| 26 | Pilot of a therapist-guided digital mental health intervention (eHealth CF-CBT) for adults with cystic fibrosis | Marieke Verkleij, Anna M Georgiopoulos, Heleen Barendrecht, Deborah Friedman [26] | Pediatric Pulmonology | 2023 |
| 27 | Patient Perspectives on the Use of Digital Technology to Help Manage Cystic Fibrosis | Alexandre H Watanabe, Connor Willis, Russell Ragsdale, Joseph Biskupiak, Karlene Moore, Diana Brixner, David Young [27] | Pulmonary Medicine | 2023 |
| 28 | Telehealth use in Australian cystic fibrosis centers: Clinician experiences | Shivanthan Shanthikumar, \| Rasa, Ruseckaite, Jen Corda Bpt, Siobhain Mulrennan, \| Sarath Ranganathan, Tonia Douglas [28] | Pediatric Pulmonology | 2023 |
| 29 | Mini-symposium: CF in the age of modulators (Part I) Telemedicine and cystic fibrosis: Do we still need face-to-face clinics? | E Dixon, K Dick, S Ollosson, D Jones, H Mattock, S Bentley, C Saunders, J Matthews, B Dobra, J King, C Edmondson, J C Davies [29] | Paediatric Respiratory Reviews | 2022 |
| 30 | Adherence, reliability, and variability of home spirometry telemonitoring in cystic fibrosis | Gary James Connett, Kelvin D Macdonald, Vikas Goyal, Fabien Beaufils, Raphaël Enaud, François Gallode, Grégory Boucher, Julie Macey, Patrick Berger, Michael Fayon, Stéphanie Bui [30] | Frontiers in Pediatrics | 2023 |
| 31 | Factors associated with receiving CF care and use of telehealth in 2020 among persons with Cystic Fibrosis in the United States | J M Collaco, D Albon, J S Ostrenga, P Flume, M S Schechter, E A Cromwell [31] | Journal of Cystic Fibrosis | 2022 |
| 32 | Telemonitoring: An opportunity in cystic fibrosis lung transplant recipients | Letizia Corinna Morlacchi, Emilia Privitera, Valeria Rossetti, Martina Santambrogio, Angela Bellofiore, Lorenzo Rosso, Alessandro Palleschi, Mario Nosotti, Francesco Blasi [32] | Heliyon | 2023 |
| 33 | Unsupervised home spirometry is not equivalent to supervised clinic spirometry in children and young people with cystic fibrosis: Results from the CLIMB-CF study | Claire Edmondson, Nicole Westrupp, Christopher Short, Paul Seddon, Catherine Olden, Colin Wallis, \| Malcolm Brodlie, \| Francis Baxter, Jonathan Mccormick, Susan Macfarlane, Richard Brooker, Margaret Connon, \| Salim Ghayyda, Lesley Blaikie, Rebecca Thursfield, Lynsey Brown, April Price, Erin Fleischer, Daniel Hughes, Christine Donnelly, Mark Rosenthal, John Wallenburg, Keith Brownlee, Eric W F W Alton, Andrew Bush, Jane C Davies, Claire Orcid, Edmondson [33] | Pediatric Pulmonology | 2023 |
| 34 | Improvement in cystic fibrosis newborn screening program outcomes with genetic counseling via telemedicine | Heather J Stalker, Amy R Jonasson, Sidney M Hopfer, Melanie Sue, Melanie Sue Collins [34] | Pediatric Pulmonology | 2023 |
| 35 | Telerehabilitation Using Fitness Application in Patients with Severe Cystic Fibrosis Awaiting Lung Transplant: A Pilot Study | Aimee M Layton, Andrew M Irwin, Erin C Mihalik, Emily Fleisch, Claire L Keating, Emily A Dimango, Lori Shah, Selim M Arcasoy [35] | International Journal of Telemedicine and Applications | 2021 |
| 36 | Experiences Among Patients With Cystic Fibrosis in the MucoExocet Study of Using Connected Devices for the Management of Pulmonary Exacerbations: Grounded Theory Qualitative Research | Maxime Morsa, Amélie Perrin, Valérie David, Gilles Rault, Enora Le Roux, Corinne Alberti, Rémi Gagnayre, Dominique Pougheon [36] | JMIR Formative Research | 2022 |
| 37 | Preferences and perspectives regarding telehealth exercise interventions for adults with cystic fibrosis: A qualitative study | Megan Poulsen, Bhlthsc Mphysioprac, Anne E Holland, Brenda Button, Arwel W Jones [37] | Pediatric Pulmonology | 2024 |
| 38 | The impact of telehealth based care on paediatric cystic fibrosis outcomes | Kristene Rimbaldo, Katherine B Frayman, Shivanthan Shanthikumar [38] | Journal of Cystic Fibrosis | 2023 |
| 39 | Impact of cystic fibrosis multidisciplinary virtual clinics on patient experience, time commitments and costs | Jody M Bell, Tiffany J Dwyer, Michelle Cunich, Ruth L Dentice, Owen Hutchings, Helen E Jo, Edmund M Lau, Wai Y Lee, Samantha A Nolan, Phillip Munoz, Freya Raffan, Karishma Shah, Miranda Shaw, Nicole A Taylor, Simone K Visser, Veronica A Yozghatlian, Keith K H Wong, Sheila Sivam [39] | Internal medicine journal (Print) | 2023 |
| 40 | Case Study: Providing Evidence-Based Behavioral and Nutrition Treatment to a Toddler With Cystic Fibrosis and Multiple Food Allergies via Telehealth | Carrie Piazza-Waggoner, Kathleen S Ferguson, Cori Daines, James D Acton, Scott W Powers [40] | Pediatric Pulmonology | 2006 |
| 41 | Q A feasibility study of home telemedicine for patients with cystic fibrosis awaiting transplantation | Olive M Wilkinson, Frances Duncan-Skingle, Jennifer A Pryor, Margaret E Hodson [41] | Journal of Telemedicine and Telecare | 2008 |
| 42 | The application of telemedicine in the follow-up of lung transplantation in a patient with cystic fibrosis | F Murgia, B Corona, F Bianciardi, P Romano, I Tagliente, S Bella [42] | Clinica Terapeutica | 2014 |
| 43 | Emerging Alternatives to Conventional Clinic Visits in the Era of COVID-19: Adoption of Telehealth at VCU Adult Cystic Fibrosis Center | Caitlin Womack, Ruhan Farsin, Mahsa Farsad, Nauman Chaudary [43] | International Journal of General Medicine | 2020 |
| 44 | Learning to breathe with Tai Chi online -qualitative data from a randomized controlled feasibility study of patients with cystic fibrosis | Patricia Ronan, Awais Mian, Siobhán B Carr, Susan L Madge, Ava Lorenc, Nicola Robinson [44] | European Journal of Integrative Medicine | 2020 |
| 45 | Tele-Exercise as a Promising Tool to Promote Exercise in Children With Cystic Fibrosis | Jen Jen Chen, Dan M Cooper, Fadia Haddad, Anna Sladkey, Eliezer Nussbaum, Shlomit Radom-Aizik [45] | Frontiers in Public Health | 2018 |
| 46 | Telehealth clinics increase access to care for adults with cystic fibrosis living in rural and remote Western Australia | Jamie Wood, Siobhain Mulrennan, Kylie Hill, Nola Cecins, Sue Morey, Sue Jenkins [46] | Journal of Telemedicine and Telecare | 2017 |
| 47 | The feasibility of online video calling to engage patients with cystic fibrosis in exercise training | Owen W Tomlinson, James Shelley, Jayne Trott, Ben Bowhay, Rohan Chauhan, Christopher D Sheldon [47] | Journal of Telemedicine and Telecare | 2019 |
| 48 | Impact of home spirometry on medication adherence among adolescents with cystic fibrosis | Aarti Shakkottai, Niko Kaciroti, Lauren Kasmikha, Samya Z Nasr [48] | Pediatric Pulmonology | 2018 |
| 49 | The use of telehealth (text messaging and video communications) in patients with cystic fibrosis: A pilot study | Michal Gur, Vered Nir, Anna Teleshov, Ronen Bar-Yoseph, Eynav Manor, Gizelle Diab, Lea Bentur [49] | Journal of Telemedicine and Telecare | 2017 |
| 50 | Eliciting the Impact of Digital Consulting for Young People Living With Long-Term Conditions (LYNC Study): Cognitive Interviews to Assess the Face and Content Validity of Two Patient-Reported Outcome Measures | Jackie Sturt, Rebecca Dliwayo, Vera Forjaz, Kathryn Hamilton, Carol Bryce, Joseph Fraser, Frances Griffiths, ©jackie Sturt, Rebecca Thandiwe, Vera Dliwayo, Kathryn Forjaz, Carol Hamilton, Joseph Bryce, Frances Fraser, Griffiths [50] | Journal of Medical Internet Research | 2018 |
| 51 | Telephone monitoring and home visits significantly improved the quality of life, treatment adherence and lung function in children with cystic fibrosis | Chrysochoou, Elisavet‐Anna, Elpis Hatziagorou, Fotis Kirvassilis, and John Tsanakas [51] | Acta Paediatrica | 2017 |
| 52 | Virtual medication tours with a pharmacist as part of a cystic fibrosis telehealth visit | Nicole Warda, Shannon M Rotolo [52] | Journal of the American Pharmacists Association | 2021 |
| 53 | Use of telemedicine in cystic fibrosis e381 Clinical and microbiological monitoring of Cystic Fibrosis patients, three years of follow-up via Tele-Medicine: an empirical research | R V De Biase, L Cristiani, C Paglia, F Alghisi, B Giordani, V Lucidi, S Bella [53] | Clinica Terapeutica | 2020 |
| 54 | Favorable Clinician Acceptability of Telehealth as Part of the Cystic Fibrosis Care Model during the COVID-19 Pandemic | Ryan C Perkins, Jaclyn Davis, Andrew Nesmith, Julianna Bailey, Michael R Powers, Nauman Chaudary, Christopher Siracusa, Ahmet Uluer, George M Solomon, Gregory S Sawicki [54] | Annals of the American Thoracic Society | 2021 |
| 55 | A Feasibility Study of Urgent Implementation of Cystic Fibrosis Multidisciplinary Telemedicine Clinic in the Face of COVID-19 Pandemic: Single-Center Experience | Martina Compton, Morgan Soper, Bonnie Reilly, Lucy Gettle, Rhonda List, Molly Bailey, Heather Bruschwein, Lindsay Somerville, Dana Albon [55] | Telemedicine journal and e-health | 2020 |
| 56 | Development of an intervention to increase adherence to nebuliser treatment in adults with cystic fibrosis: CFHealthHub | M A Arden, M Hutchings, P Whelan, S J Drabble, D Beever, J M Bradley, D Hind, J Ainsworth, C Maguire, H Cantrill, A O'cathain, M Wildman [56] | Pilot and Feasibility Studies | 2020 |
| 57 | Remote support by multidisciplinary teams: A crucial means to cope with the psychological impact of the SARS-COV-2 pandemic on patients with cystic fibrosis and inflammatory bowel disease in Lombardia | Rita Maria Nobili, Simone Gambazza, Maria Simonetta Spada, Anna Luisa Tutino, Anna Marta Bulfamante, Alessandra Mariani, Anna Brivio, Loredana Moioli, Erika Rizzato, \| Naire Sansotta, Laura Claut, Nadia Faelli, \| Lorenzo Norsa, Carla Colombo [57] | International Journal of Clinical Practice | 2021 |
| 58 | Telehealth use in cystic fibrosis during COVID-19: Association with race, ethnicity, and socioeconomic factors R | Dana Albon, Aricca D Van Citters, Thida Ong, Olivia Dieni, Christopher Dowd, Anne Willis, Kathryn A Sabadosa, Peter Scalia, Kimberly Reno, Gabriela R Oates, Michael S Schechter [58] | Journal of Cystic Fibrosis | 2021 |
| 59 | Telerehabilitation for Lung Transplant Candidates and Recipients During the COVID-19 Pandemic: Program Evaluation | Lisa Wickerson, Bscpt, Denise Helm, Chaya Gottesman, Dmitry Rozenberg, Lianne G Singer, Shaf Keshavjee, Aman Sidhu [59] | JMIR mHealth and uHealth | 2021 |
| 60 | Video Consultation During the COVID-19 Pandemic: A Single Center's Experience with Lung Transplant Recipients | Moritz Z Kayser, Christina Valtin, Mark Greer, Bernd Karow, Jan Fuge, Jens Gottlieb [60] | Telemedicine journal and e-health | 2020 |
| 61 | Cystic fibrosis telemedicine in the era of COVID-19 | Elika J Rad, Alicia A Mirza, Laveena Chhatwani, Natasha Purington, Paul K Mohabir [61] | JAMIA Open | 2022 |
| 62 | Telehealth application of an ultrasonic home spirometer | Michael Doumit, Rianna Ledwos, Leanne Plush, Sandra Chuang, Melinda Gray, Adam Jaffe, Jamie Mcbride [62] | Archives of Disease in Childhood | 2022 |
| 63 | "You're on mute!" Does pediatric CF home spirometry require physiologist supervision? | Emma Fettes, Mollie Riley, Stephanie Brotherston, Claire Doughty, Benjamin Griffiths, Aidan Laverty, Paul Aurora [63] | Pediatric Pulmonology | 2022 |
| 64 | Telephone surveillance during the COVID-19 pandemic: Is it a helpful diagnostic tool for detecting acute pulmonary exacerbations in children with chronic lung disease? | Beste Ozsezen, Nagehan Emiralioglu, Dilber A Tural, Birce Sunman, Halime N Buyuksahin, Ebru Yalcin, Deniz Dogru, Ugur Ozcelik, Nural Kiper [64] | Journal of Telemedicine and Telecare | 2020 |
| 65 | Web-based physical activity promotion in young people with CF: a randomised controlled trial | Narelle S Cox, Beverley Eldridge, Sarah Rawlings, Julianna Dreger, Jennifer Corda, Jennifer Hauser, Brenda M Button, Jennifer R Bishop, Amanda Nichols, Anna Middleton, Nathan Ward, Tiffany Dwyer, Ruth Dentice, Raynuka Lazarus, Paul O'halloran, Joanna Y T Lee, Christie Mellerick, Kelly Mackintosh, Melitta Mcnarry, Craig Anthony Williams, Anne E Holland [65] | Thorax | 2022 |
| 66 | Home-spirometry exacerbation profiles in children with cystic fibrosis | Benoit Bouteleux, Fabien Beaufils, Michael Fayon, Stéphanie Bui [66] | Pediatric Pulmonology | 2024 |
| 67 | Patient factors associated with telehealth quality and experience among adults with chronic conditions | Esther Yoon, Scott Hur, Laura M Curtis, Julia Yoshino Benavente, Michael S Wolf, Marina Serper [67] | JAMIA Open | 2024 |
| 68 | Online activity -A beaming good initiative! Delivering alternative exercise opportunities for people with cystic fibrosis | L Morrison, G Mccrea, S Palmer [68] | Physiotherapy Theory and Practice | 2024 |
| 69 | Telemonitoring for Cystic fibrosis patients of Bambino Gesù Children's Hospital during COVID-19 | A Federici, M De Marchis, F Alghisi, A G Fiocchi, S Bella [69] | Clinica Terapeutica | 2022 |
| 70 | Telehealth Use in Pediatric Care during the COVID-19 Pandemic: A Qualitative Study on the Perspectives of Caregivers | Gergana Damianova Kodjebacheva, Charlotte Tang, Faith Groesbeck, Loretta Walker, Jillian Woodworth, Jennifer Schindler-Ruwisch [70] | Children | 2023 |
| 71 | ACT with CF: A telehealth and in-person feasibility study to address anxiety and depressive symptoms among people with cystic fibrosis | C Virginia O'hayer, Caitlin M O'loughlin, Chelsi N Nurse, Patrick J Smith, Michael J Stephen [71] | Journal of Cystic Fibrosis | 2020 |
| 72 | Mobile health platform for self-management of pediatric cystic fibrosis: Impact on patient-centered care outcomes | Gabriela R Oates, Cathy Mims, Robin Geurs, Rikard Bergquist, Andreas Hager, Jennifer S Guimbellot, Anastasia M Hartzes, Hector H Gutierrez [72] | Journal of Cystic Fibrosis | 2023 |
| 73 | Preserving Multidisciplinary Care Model and Patient Safety During Reopening of Ambulatory Cystic Fibrosis Clinic for Nonurgent Care: A Hybrid Telehealth Model | Rhonda List, Martina Compton, Morgan Soper, Heather Bruschwein, Lucy Gettle, Molly Bailey, Elissa Starheim, John Kalmanek, Lindsay Somerville, Dana Albon [73] | Telemedicine journal and e-health | 2020 |
| 74 | Family perception of a telehealth program for people with cystic fibrosis during the COVID-19 pandemic in northeastern Brazil | Adriana V B Faiçal, Edna L Souza, Regina Terse-Ramos [74] | Jornal Brasileiro de Pneumologia | 2023 |
| 75 | Development of an Interdisciplinary Telehealth Care Model in a Pediatric Cystic Fibrosis Center | Catherine Enochs, Amy G Filbrun, Courtney Iwanicki, Haley Moraniec, Julie Lehrmann, Jourdan Stiffler, Sharyn Dagher, Chris Tapley, Hanna Phan, Rebekah Raines, Samya Z Nasr [75] | Telemedicine Reports | 2021 |
| 76 | Patient and family perspectives regarding the use of telehealth for cystic fibrosis care | Shivanthan Shanthikumar, Emily Moore Mpt, Jen Corda Bpt, Nicola Reardon Bnurs, \| Stef, Louey, Katherine Frayman, Jo Harrison Mclined, \| Sarath Ranganathan [76] | Pediatric Pulmonology | 2021 |
| 77 | Home spirometry utilisation in telemedicine clinic for cystic fibrosis care during COVID-19 pandemic: a quality improvement process | Martina Compton, Rhonda List, Elissa Starheim, Lindsay Somerville, Lauren Williamson, Rachel Murray, Deirdre Jennings, Heather Bruschwein, Dana Albon [77] | BMJ Open Quality | 2021 |
| 78 | Telemedicine Use and Health-Related Concerns of Patients With Chronic Conditions During COVID-19: Survey of Members of Online Health Communities | Lindsey Nicole Horrell, Sara Hayes, ; Leslie, Beth Herbert, Katie Macturk, Lauren Lawhon, Carmina G Valle, ; Amrita Bhowmick, Nicole Horrell, Beth Leslie, Katie Herbert, Lauren Macturk, Carmina G Lawhon, Amrita Bhowmick Valle [78] | Journal of Medical Internet Research | 2020 |
| 79 | A multi-centre, randomized, controlled trial on coaching and telemonitoring in patients with cystic fibrosis: conneCT CF | Stephanie Thee, Mirjam Stahl, Rainald Fischer, Sivagurunathan Sutharsan, Manfred Ballmann, Axel Müller, Daniel Lorenz, Dominika Urbanski-Rini, Franziska Püschner, Volker Eric Amelung, Carola Fuchs, Marcus Alexander Mall [79] | BMC Pulmonary Medicine | 2021 |
| 80 | Feasibility and implementation of a personalized, web-based exercise intervention for people with cystic fibrosis for 1 year | Barlo Hillen, Perikles Simon, Sebastian Schlotter, Oliver Nitsche, Viola Bähner, Krystyna Poplawska, Daniel Pfirrmann [80] | BMC Sports Science, Medicine and Rehabilitation | 2021 |
| 81 | Implementation and evaluation of a fertility preservation telehealth counseling intervention for males with cystic fibrosis | Brittany M Woods, Leigh A Bray, Sukhkamal B Campbell, Peng Li, Traci M Kazmerski, Cade Hovater, Leslie N Pitts, Sigrid Ladores [81] | Journal of Cystic Fibrosis | 2024 |
| 82 | Exercise intolerance, oxidative stress, and irisin in pediatric cystic fibrosis: Can telehealth-based exercise training make a difference? | Kubra Kilic, Naciye Vardar-Yagli, Halime Nayir-Buyuksahin, Ismail Guzelkas, Deniz Dogru, Melda Saglam, Ebru Calik-Kutukcu, Deniz Inal-Ince, Nagehan Emiralioglu, Ebru Yalcin, Ugur Ozcelik, Nural Kiper [82] | Heart & Lung | 2024 |
| 83 | Coping and learning to Manage Stress with cystic fibrosis (CALM): A multisite telehealth randomized controlled trial to reduce depression and anxiety symptoms in adults with cystic fibrosis | Christina J Bathgate, Elizabeth D Smith, Nora H Murphy, Alexandra L Quittner, Kristin A Riekert, Jennifer L Goralski, Kristen E Holm [83] | Journal of Cystic Fibrosis | 2024 |
| 84 | Increasing Access to Genetic Counselors With Disease-Specific Expertise: Development of a Centralized Cystic Fibrosis Genetic Counseling Telehealth Model | Elinor Langfelder-Schwind, Melissa Basile, Rachel Moyal-Smith, Jennifer Polo, Molly A Mcginniss, Jenna Petersen, Josua Talavera, Haylee Schwind, Richard B Parad, Karen S Raraigh [84] | Pediatric pulmonology | 2025 |
| 85 | The effect of a 12-week tele-exercise using immersive virtual reality on functional capacity in adolescents with cystic fibrosis: A randomized controlled, single (assessor) -blind study | Ozyemisci Ozden, Taskiran, Havvanur Albayrak, Kog, Ecenur Atli, Erdem Gonullu, Asim Evren Yantac, Zeynep Seda Uyan [85] | Respiratory Medicine | 2025 |
| 86 | Perceptions of Telehealth in Pediatric Pulmonary Clinic Post-Pandemic | Jaclyn Davis, Sidney L Gibson \|, Ryan C Perkins \|, Jonathan Greenberg \|, Tregony Simoneau \|, Gregory S Sawicki [86] | Pediatric pulmonology | 2025 |
| 87 | Telemedicine and home spirometry in cystic fibrosis: A prospective multicenter study | Jakob Medbo, Henrik Imberg, Christine Hansen, Christina Krantz, Isabelle De Monestrol, Marcus Svedberg, Petter Silverskiolds [87] | Pediatric pulmonology | 2024 |
| 88 | Acceptability of Telehealth Post-Pandemic Among Clinicians Across the United States Caring for People With Cystic Fibrosis | Jaclyn Davis, \| Ryan Perkins, Julianna Bailey, \| Nauman Chaudary, \| Bryan Garcia, Deborah Froh, Mike Powers, Christopher Siracusa, Gregory S Sawicki [88] | Pediatric pulmonology | 2025 |
| 89 | Impact of UPLIFT, a group telehealth intervention, on symptoms of depression and anxiety in adults with CF | Michael S Schechter, Andrea Molzhon, Robin S Everhart, Le Kang, Rachel Weiskittle, Brittany Castleberry, Nancy J Thompson [89] | Journal of Cystic Fibrosis | 2024 |
| 90 | Remote assessment of exercise capacity in adults with chronic respiratory disease: Safety, reliability and acceptability | Narelle S Cox, Simone Dal Corso, Angela T Burge, Janet Bondarenko, Jaycie Perryman, Anne E Holland [91] | Chronic Respiratory Disease | 2025 |
| 91 | Feasibility of performing the 3-minute step test with remote supervision in children and adolescents with cystic fibrosis: A comparative study | Fernanda Maria Vendrusculo, Gisele Apolinário Da Costa, Maria Amélia Bagatini, Brenda Maria Henrique, Maia Lemes, Carolina Aguiar Faria, Larissa Carvalhaes De Oliveira, Evanirso Da, Silva Aquino, Márcio Vinícius, Fagundes Donadio [92] | Pediatric Investigation | 2025 |
| 92 | ACT with CF: A randomized trial of acceptance and commitment therapy vs supportive psychotherapy for adults with cystic fibrosis | C Virginia O'hayer, Patrick J Smith, Christopher F Drescher, Heather Bruschwein, Chelsi N Nurse, Hallie M Kushner, Krista Ingle, Michael J Stephen, Jeffrey B Hoag, Thomas Jefferson [93] | General Hospital Psychiatry | 2024 |
| 93 | Understanding the acceptability of the changing model of care in cystic fibrosis | Michael Doumit, Verity Pacey, Adam Jaffe, Kelly Gray [90] | Respiratory Medicine | 2024 |
| 94 | Cystic Fibrosis Learning Network Telehealth Innovation Lab During the COVID-19 Pandemic: Impact on Access to Care, Outcomes, and a New CF Care Model | Dana Albon, \| Thida Ong, Bethany Horton, \| David Brighton, Shiyi Shen, Rhonda List, Nicholas Antos, Fadi Asfour, Ella Balasa, Danielle Beachler, \| Cori Daines, Deborah Froh, Catherine Kier, Samya Nasr, Meghana Sathe, Gregory Sawicki, Michael Schechter, George Solomon, Michael Powers, Kathy Sabadosa, Bruce Marshal, Lacrecia Thomas, Michael Seid, Maria Britto, Raouf Amin, Elizabeth Cromwell, T H Ilab, Working Group, Glenda Drake, Amy Lucero, Lilly Mees, Amanda Sharpe, Rachel Gordon, Lucinda Murphy, Ahmet Uluer, Lindsey Mcmahon, Melanie Abdelnour, Meghan Murray, Preeti Sharma, Traci Liberto, Rachel Linnemann, Sydney Schiff, Alexia Hernandez Cargal, Heather Tucker, Pornchai Tirakitsoontorn, Maivy Sou, Alicia Maciel, Andrea Molzhon, Nicole Brueck, Karen Wunschel, Christopher M Siracusa, Lisa A Mullen, Kyle Traver, Travis Burgett, Alex Gifford, Nicola Felicetti, Heidi Dolan, Tracey Gendreau, Shine-Ann Pai, Stephanie Robbins, Zoe Orcutt, Ben Mccullar, Lindsaey Deveaux, Christian Merlo, Lauren Mitchell, Andrew Scaljon, Rebekah Brown, Stefanie Rushing, Virginia Birnbaum, Hossein Sadeghi, Golnar Raissi, Bean Corcoran, Michelle Prickett, Rachel Nelson, Joanne Cullina, Anne Snuggerud, Stacy Allen, Sabrina Gorry, Lisa Greene, Peter Michelson, Sara Renschen, Mike Price, Betsy Price, Teresa Carney, Sandy Corr, Barbara Leyva, Jillian Salvatore, Julianna Bailey, James Lawlor, Rebekah Raines, Catherine Enochs, Jordan Dunitz, Kristen Jesse, Kayla Warner, Jonathan Flath, Mackenzie Wharram, Holly Carroll-Owens, Lauren Williamson, Lindsay Sommerville, James Tolle, Susan Eastman, Marrisa Benchea, Nauman Chaudary, Mahsa Farsad, Kimberly Wingo, Kathryn Moffett, Erin Brozik, Jillian Huffman [94] | Pediatric Pulmonology | 2025 |
| 95 | Impact of telehealth during the COVID-19 pandemic on clinical and nutritional conditions of adolescents with cystic fibrosis | Lavínia Mayara, Silva Reis, Aline Antunes De, Cerqueira Pinheiro, Maurício Antônio, Silva Júnior, Christine Pereira Gonçalves, Nelbe Nesi Santana [95] | Jornal Brasileiro de Pneumologia | 2024 |
| 96 | WEB-RE HAB Program for people with cystic fibrosis during COVID-19 pandemic: a feasibility study | Matteo De Marchis, Marco Rivolta, Sara De Dominicis, Mario Ciarnella, Paola Leone, Francesco Milo, Daniele Di Giovanni, Carmen D 'amore, Alessandra Federici, Alessandra Boni, Federico Alghisi, Sergio Bella, Mario Cannataro, Renato Cutrera [96] | Minerva Respiratory Medicine | 2025 |
| 97 | The impact of telehealth on clinical outcomes in adults and children with cystic fibrosis in Australia | Tonia A Douglas, Ahmad Reza Pourghaderi, Susannah Ahern, Jen Corda, Arul Earnest, Siobhain Mulrennan, Sarath Ranganathan, Rasa Ruseckaite, Shivanthan Shanthikumar [97] | Journal of Cystic Fibrosis | 2025 |
| 98 | Remote exercise services for people with cystic fibrosis: experiences and perceptions from people with cystic fibrosis and members of cystic fibrosis multidisciplinary teams | Yue Qin, Katy Hamana, Nichola Gale [98] | Disability and Rehabilitation | 2025 |

**Table S3.** Summary of the final 98 studies [1-98], including study design, population, research objective, country of origin, telehealth format, and key findings.

| **Study ID** | **Study Design** | **Population** | **Research Objective** | **Country of origin** | **Telehealth format** | **Key findings** |
| --- | --- | --- | --- | --- | --- | --- |
| **1** | Qualitative evaluation | Dutch adults with CF (N = 16) and CF healthcare providers (N = 16) | Evaluate usability and acceptability of eHealth CF-CBT | United States and the Netherlands. | Video call (virtual sessions) | - Primary findings: High overall impression and usability ratings by patients and healthcare providers; positive eHIQ and SUS scores.  - Effect sizes/statistical significance: Mean scores for overall impression (8.3 for patients, 8.2 for providers), usability (77.0 for patients, 73.4 for providers), eHIQ domains (Motivation and Confidence to Act: 71.3, Information and Presentation: 78.9, Identification: 62.0).  - Secondary findings: The program is the first therapist-guided internet-delivered intervention for adults with CF; it has potential for increasing access to mental health care.  - Authors' main conclusions: The program is highly acceptable and usable; it addresses an urgent need for new mental health care approaches in CF patients.  - Clinical/practical significance: The program can be integrated into existing care models; it has potential for increasing access to mental health care.  - Unexpected/contradictory findings: Limited generalizability due to small sample size and predominantly female participants. |
| **2** | Pre-post design, cohort design | 16 pwCF, 14 parents; demographics: pwCF (9 female, 7 male, ages 12-36), parents (13 mothers, 1 father, ages 26-49) | Evaluate effectiveness of telehealth psychological support intervention in reducing stress, depression, and anxiety | Italy | Video call (using Zoom) | - Primary findings: Significant reductions in stress and depression for pwCF and parents; no significant reduction in anxiety.  - Effect sizes: Large effect for stress reduction (Cohen's d = 1.02), medium to large effect for depression reduction (Cohen's d = 0.69).  - Secondary findings: Positive feasibility and satisfaction ratings; intervention may have prevented worsening of symptoms.  - Authors' main conclusions: Telehealth intervention effective in reducing stress and depression; feasible and satisfactory.  - Clinical significance: Effective in reducing stress and depression during COVID-19 lockdown.  - Limitations: No control group; shorter than traditional CBT; small sample size.  - Unexpected findings: Anxiety did not significantly decrease. |
| **3** | Cross-sectional survey-based study | 261 participants (120 adult patients, 141 parents/guardians of pediatric patients) | To understand how people with CF and their families experienced the shift to telehealthcare delivery during the COVID-19 pandemic | United States | Video calls using cellphones, computers, and tablets. | - Primary findings: High satisfaction rates among adult (77%) and pediatric (72%) patients with telehealth visits; high convenience ratings (85%).  - Secondary findings: Concerns about lack of in-person assessments like pulmonary function testing and throat/sputum cultures; strong interest in future use of telehealth.  - Authors' conclusions: Telehealth is feasible and well-accepted for chronic CF care during the pandemic and potentially beyond; further research needed on patient outcomes and healthcare utilization.  - Clinical significance: Telehealth can continue to play a role in CF care post-pandemic, addressing concerns about in-person assessments with remote monitoring technologies. |
| **4** | Descriptive/Observational | Cystic fibrosis patients with related diabetes (CFRD) at the Cleveland Clinic Cystic Fibrosis center; sample size not explicitly mentioned | To report on changes implemented to optimize diabetes management in CF patients through telehealth during the COVID-19 crisis | United States | The telehealth format includes virtual visits (video calls), phone calls, and remote monitoring using CGM systems (Freestyle Libre, Dexcom G6). | - Primary findings: Low incidence of COVID-19 among CF patients at the Cleveland Clinic CF center; effective transition to telehealth model for managing CFRD.  - Secondary findings: Use of telehealth and CGM systems improves glycemic control and diabetes self-management; low incidence of COVID-19 attributed to social distancing and telehealth adoption.  - Authors' main conclusions: Telehealth is effective in managing CFRD during the pandemic; CF patients' familiarity with social distancing and protective equipment contributes to lower COVID-19 incidence.  - Clinical significance: Telehealth and CGM systems are crucial for maintaining CFRD management during the pandemic.  - Unexpected findings: Low incidence of COVID-19 in CF patients compared to the general population. |
| **5** | Observational benchmarking study | Young people aged 8-18 years with cystic fibrosis, 82 episodes of care from 51 patients | To determine if clinical outcomes with a hybrid telehealth model are equitable to standard care | Australia | Video conferencing (telehealth videocall with live feed camera and audio) using smartphones, tablets, or laptops. | - Primary findings: 49% of the hybrid group and 32% of the standard care group achieved at least 95% of baseline ppFEV1. Median ppFEV1 change was +6% for the hybrid group and +2% for the standard care group.  - Effect sizes: Median ppFEV1 change was significantly higher in the hybrid group.  - Secondary findings: No adverse events were reported. Estimated travel time and distance saved were 16,520 minutes and 12,301.2 kilometers.  - Authors' main conclusions: The hybrid telehealth model is a viable alternative to standard care with no added risk and improved efficiency.  - Clinical significance: The study supports the use of telehealth in pediatric respiratory exacerbations, improving efficiency and patient care.  - Unexpected findings: The gender disparity in the hybrid group, with more females than expected, was an unexpected finding. |
| **6** | Randomized controlled trial (RCT) | Individuals with cystic fibrosis (CF) at least 14 years old; sample size: 267 participants | Evaluate whether home monitoring of pulmonary exacerbations (PE) is a cost-minimizing intervention | United States | The telehealth format includes home spirometry for lung function measurement, regular symptom tracking using the CF Respiratory Symptom Diary (CFRSD), remote monitoring with automatic alerts for significant changes, and telephone calls from Respiratory Therapists for orientation and reminders. | - Primary findings: Outpatient visit costs were significantly higher in the EI arm compared to the UC arm, but no significant differences in other healthcare costs or total healthcare costs.  - Effect sizes: 13% increase in outpatient visit costs in EI arm; lower mean total costs in EI arm due to lower antibiotic costs.  - Secondary findings: Adherence to intervention associated with lower hospitalization costs but higher antibiotic costs; significant change in CRISS score indicating symptom management benefit.  - Authors' conclusions: Home spirometry does not improve lung function or reduce healthcare costs; adherence and cost variability are important considerations.  - Clinical significance: No cost justification or improvement in lung function from home spirometry; potential benefit in symptom management.  - Unexpected findings: Higher outpatient visit costs in EI arm; adherence associated with higher antibiotic costs. |
| **7** | Observational | 120 patients with cystic fibrosis (CF) or their caregivers | Assess the impact of remote monitoring on patients' health status and daily maintenance therapy | Greece (inferred from context and authors' names) | Phone call | - Primary findings: 120 CF patients were monitored via telephone calls; 28.33% experienced pulmonary exacerbations, with most treated with oral antibiotics; most patients maintained daily physiotherapy frequency, but 71% changed exercise type and frequency.  - Effect sizes/statistical significance: Mean FEV1 and body weight increased significantly after lockdown.  - Secondary findings: Early detection and management of pulmonary exacerbations and CF complications; fewer chest infections and dietary changes may have contributed to increased FEV1 and weight gain.  - Authors' main conclusions: Telephone contact processes are crucial for CF care during the pandemic; further studies are needed to evaluate outcomes when transitioning to telemedicine.  - Clinical significance: Remote monitoring can effectively manage CF complications; lockdown measures may have contributed to improved lung function and weight gain.  - Unexpected findings: Increase in FEV1 and body weight during lockdown. |
| **8** | Qualitative, semi-structured interviews | Partners of women with cystic fibrosis (n = 20) and cystic fibrosis healthcare providers (n = 20) | Explore perceptions of incorporating telehealth into routine cystic fibrosis healthcare | United States | Video conferencing using web-based platforms (e.g., Zoom) | - Primary findings: Increased connection between healthcare team and family, increased efficiency of healthcare appointments, and improved interdisciplinary collaboration.  - Secondary findings: Partners found telehealth allowed for more engagement in healthcare; providers reported enhanced efficiency and collaboration opportunities.  - Authors' main conclusions: Telehealth has potential benefits beyond the pandemic; it is a cost-effective, efficient, and comprehensive alternative to traditional care delivery.  - Clinical significance: Telehealth improves care delivery and coordination, especially for chronic conditions.  - No effect sizes or statistical significance reported due to qualitative nature of the study. |
| **9** | Qualitative analysis using focus groups and semi-structured interviews | CF program directors and coordinators from 12 programs for telehealth quality and 8 programs for reimbursement | Qualitative exploration of facilitators and barriers to implementing high-quality telehealth and navigating reimbursement | United States | The telehealth format included a combination of video calls (implied by the use of telehealth software and systems), remote monitoring (such as home spirometry), and possibly telephone-only telehealth. The exact formats are not explicitly detailed in the paper, but these are inferred from the discussion of telehealth infrastructure and reimbursement challenges. | - Primary findings: Factors differentiating programs with higher and lower perceived telehealth quality include telehealth characteristics, external influences, program characteristics, team member characteristics, and implementation processes. Reimbursement barriers include billing issues, multidisciplinary team reimbursement, and lower patient volumes.  - Secondary findings: Successful programs overcame barriers by embracing telehealth as a priority and recognizing its advantages.  - Authors' main conclusions: Future efforts should provide guidance and incentives to support telehealth delivery and infrastructure, share best practices, and remove barriers.  - Clinical significance: Findings have implications for clinical practice, research, and policy, suggesting a hybrid approach combining telehealth with in-person care.  - Unexpected or contradictory findings: Not mentioned. |
| **10** | Mixed methods (surveys and qualitative analysis) | People with cystic fibrosis (PwCF) and cystic fibrosis programs; PwCF sample size: 424; Program sample size: 286 (SoC1), 280 (SoC2) | Characterize experiences with remote monitoring and identify perspectives for future care | United States | Remote monitoring (including home spirometry, scales, and pulse oximeters) and remote sample collection (including arrangements for phlebotomy) | - Primary findings: Distribution of 13,345 spirometers by October 2020 and 19,271 by May 2021; increased access to home devices (spirometers: 30% to 70%, scales: 50% to 70%, oximeters: 5% to 10%); PwCF access to scales (89%), oximeters (48%), and spirometers (47%); frequent use of scales and oximeters weekly, spirometers monthly.  - Effect sizes/statistical significance: Significant increase in device distribution and access; p-values for differences in access to devices between program types and regions.  - Secondary findings: Lack of method for collecting respiratory specimens for cultures in some programs; most programs had a process for phlebotomy.  - Authors' main conclusions: Remote monitoring is crucial for future care; improvements needed in access, training, and data collection systems.  - Clinical/practical significance: Increased convenience, reduced infection risks, empowerment for self-monitoring; need for patient engagement and assessment of tradeoffs.  - Unexpected/contradictory findings: Not explicitly mentioned. |
| **11** | Prospective cross-sectional study | 144 pediatric CF patients (74 males, 70 females; mean age 8.9 years) and their caregivers; 49 healthy age-and gender-matched controls and their caregivers | To monitor the health and nutritional status of pediatric CF patients via telehealth services during the COVID-19 pandemic | Turkey | The telehealth format included phone calls and video calls. | - Primary findings: Low COVID-19 transmission rate among CF patients; lower anxiety levels in CF patients compared to controls; higher anxiety and depression in caregivers.  - Effect sizes/statistical significance: STAI scores significantly lower in CF patients (p < 0.001); HAD anxiety and depression scores significantly higher in caregivers (p = 0.005 and p < 0.001).  - Secondary findings: Significant improvement in nutritional status and weight gain during the pandemic.  - Authors' main conclusions: Telehealth services are effective in reducing anxiety and improving nutritional status; CF patients require psychiatric evaluation and psychosocial support.  - Clinical significance: Telehealth services are beneficial for managing CF-related complications and reducing anxiety. |
| **12** | Cross-sectional study | CF patients (ages 0-22 years) and their families, CF care providers (46 families, 24 providers) | To understand the telemedicine experience and interest in continuing its use | United States | The telehealth format used in this study was primarily video conferencing via Zoom, with the use of breakout rooms for multidisciplinary care. It also included telephone calls for cases where video was not feasible. There was potential for remote monitoring, such as home spirometry, but this was not a primary focus of the study. | - Primary findings: High satisfaction rates among families (80%) and providers (88%) with telemedicine; 72% of families want future telemedicine visits.  - Effect sizes: 80% family satisfaction, 88% provider satisfaction, 72% want future telemedicine visits.  - Secondary findings: Benefits include reduced travel time, cost savings, and avoiding COVID exposure; differences in provider preferences.  - Main conclusions: Telemedicine is well-received, but addressing disparities is crucial; improves clinic attendance and adherence to guidelines.  - Clinical significance: Telemedicine can reduce logistical burdens while maintaining care quality; need for future studies on long-term satisfaction and quality. |
| **13** | Cross-sectional survey | 424 participants (222 adults with CF, 201 parents of children with CF), distributed across the United States | To explore patient and family perceptions of telehealth in the CF care model | United States | The telehealth format primarily involved video connections (88%), with some phone-only visits (12%). | - Primary findings: Most participants had a telehealth visit with a multidisciplinary team, found telehealth easy to use, and perceived its quality as similar or higher than in-person care.  - Effect sizes/statistical significance: 91% found telehealth easy to use; 66% reported similar/higher quality than in-person care; adults more likely to desire future telehealth (64% vs. 36%); p = .021 for desire between adults and parents.  - Secondary findings: One-third of participants desired future telehealth; perception of quality predicts desire for future telehealth.  - Authors' main conclusions: Positive perception of telehealth in CF care; need for improvement in technical issues and at-home monitoring.  - Clinical/practical significance: Telehealth is seen as a viable option for CF care, with a need for continued improvement.  - Unexpected/contradictory findings: None explicitly mentioned. |
| **14** | Cross-sectional | CF care programs (278 at timepoint one, 274 at timepoint two), patients and families (424 respondents) | Understand the state of cystic fibrosis care during the COVID-19 pandemic | United States | The telehealth format included phone calls and video calls, with some visits involving a combination of phone and video components (phone/computer with video). | - Primary findings: Most CF programs provided telehealth during the pandemic; 81% of patients and families reported having a telehealth visit.  - Effect sizes/statistical significance: The percentage of telehealth visits decreased from 45% to 25% over time; 80% of visits were in-person by May 2021.  - Secondary findings: Telehealth is likely to become a mainstream option for chronic care; datasets are valuable for exploring barriers and facilitators.  - Authors' main conclusions: The pandemic accelerated telehealth adoption; datasets are valuable for further analysis.  - Clinical/practical significance: Telehealth may become a mainstream option for chronic care management.  - Unexpected/contradictory findings: Not explicitly mentioned. |
| **15** | Cross-sectional survey study | 18 patients with CF (9 with CFRD), 21 providers (adult and pediatric endocrinologists) | Assess patient and provider perspectives on telemedicine for CF and CFRD | United States (specifically, New York) | The telehealth format includes video calls, telephone calls, and remote monitoring. Patients primarily use phones for visits, while providers use computers/laptops. Multiple platforms are used, and telemedicine is integrated into electronic health systems. | - Primary findings: High satisfaction with telemedicine among patients (83.3%) and providers (71.4%), convenience (94.4% patients, 85.7% providers), and adequate time during visits (94.4% patients, 76.2% providers).  - Effect sizes/statistical significance: Providers were more concerned about lack of in-person examination components (height/weight, vitals, glycated hemoglobin) with p < 0.001.  - Secondary findings: Ease of attending telemedicine appointments for patients, decrease in "no shows" for providers.  - Authors' main conclusions: Telemedicine has high satisfaction rates but lacks physical examination components; it does not replace traditional visits.  - Clinical significance: Telemedicine improves appointment attendance rates and is convenient, but lacks physical examination components.  - Unexpected findings: No significant difference in provider perceptions between CFRD and type 1 diabetes treatment. |
| **16** | Single-blind randomized trial | 28 patient-caregiver dyads (children aged 6-13 with CF) | To examine the effect of telerehabilitation on quality of life, depression, and anxiety levels in children with CF and their caregivers | Turkey (inferred from context and authors' names) | Video call (via Zoom) | - Primary findings: Significant improvements in patients' anxiety and depression levels (RCADS-Major depressive disorder score decreased from 6.21 to 3.92, p < 0.02; RCADS-generalized anxiety disorder score decreased from 6.28 to 3.42, p < 0.01).  - Effect sizes/statistical significance: Significant improvements in body image (p = 0.04).  - Secondary findings: No significant changes in caregivers' anxiety and depression levels.  - Authors' main conclusions: Telerehabilitation improves anxiety, depression, and functional status in CF patients but not quality of life or pulmonary function.  - Clinical significance: Telerehabilitation is a safe and effective approach for improving psychological and functional outcomes in CF patients.  - Limitations: Small-scale study; larger studies needed for robust results. |
| **17** | Quality Improvement (QI) initiative | 29 CF care centre teams (10 adult, 19 paediatric) from the Cystic Fibrosis Learning Network | Increase virtual visits with interdisciplinary care and shared agenda setting | United States | The telehealth format included video calls (telemedicine), phone calls, and remote monitoring (such as home spirometry). | - Primary findings: The TH ILab achieved its specific aims ahead of schedule, with 85% of visits providing IDC and 92% of patients participating in AS by August 2020.  - Effect sizes: Exceeded goals for IDC and AS by December 31, 2020.  - Secondary findings: Factors supporting IDC and AS included robust communication processes and standardized pre-visit planning.  - Authors' main conclusions: TH is a valuable tool for CF care during the pandemic, but further work is needed to address equity and institutional barriers.  - Clinical significance: TH can meet quarterly visit guidelines and improve care access.  - Practical significance: Processes developed in the TH ILab can be spread to other teams with strong QI infrastructure.  - Theoretical significance: Highlights the potential of telehealth in CF care and the need for continued improvement. |
| **18** | Feasibility study | Adult CF patients; sample size = 80; demographics: mean age = 31 years, 49% female | Investigate feasibility of replacing in-person appointments with remote monitoring using telephone consultations and PRO data | Denmark | Telephone consultations combined with remote monitoring (home spirometry and sputum sample collection) | - Primary findings: Remote monitoring using telephone consultations combined with PRO data was found to be acceptable and useful by both patients and HCPs.  - Effect sizes/statistical significance: Not explicitly mentioned in the text.  - Secondary findings: Patients appreciated flexibility and relevance but suggested improvements; need for further development of the questionnaire and better communication about the solution's purpose and workflow.  - Authors' main conclusions: The PRO-based remote follow-up solution is feasible and safe; patient involvement is crucial for future development.  - Clinical/practical significance: The solution provides flexibility and relevance but needs further development to address mental health and workflow issues.  - Unexpected/contradictory findings: The solution has both advantages (e.g., increased control) and disadvantages (e.g., increased focus on disease). |
| **19** | Cross-sectional, descriptive study | Children and adolescents with cystic fibrosis; sample size = 184; mean age = 7.0±0.4 years; 47.3% male | Describe the experience of implementing routine teleconsultations in respiratory physiotherapy during the COVID-19 pandemic | Brazil | The telehealth format included videoconferences (using Skype) and telephone calls for teleconsultations, as well as telemonitoring for remote monitoring of health and disease parameters. | - Primary findings: 83.2% of patients participated in teleconsultations; no statistical difference between participants and non-participants except for bacterial colonization.  - Effect sizes/statistical significance: Statistically significant difference in bacterial colonization distribution (p<0.05).  - Secondary findings: High acceptance of telemedicine; potential for permanent use; benefits include increased accessibility and reduced hospital visits.  - Authors' main conclusions: Telemedicine is positive and may become permanent; it increases accessibility and reduces hospital visits.  - Clinical significance: Telemedicine can reduce cross-infection risk and social impact; challenges include technology access and remote exam limitations. |
| **20** | Randomized controlled mixed-methods pilot study | 88 adults with cystic fibrosis, aged 18 years or older, with a history of at least one hospital admission for intravenous antibiotics in the past 24 months | To assess the effectiveness of home monitoring compared to routine care in reducing hospital inpatient bed days and improving health-related quality of life | United Kingdom | Remote monitoring (home spirometry) and communication via phone/text message | - Primary findings: HM detected more PEx than RC, but no significant differences in hospital inpatient bed days or overall health-related quality of life.  - Effect sizes/statistical significance: Total mean NHS costs were approximately £1500 more per patient for RC than HM.  - Secondary findings: HM was generally well accepted, with positive impacts on patient self-awareness and self-care strategies.  - Authors' main conclusions: HM is effective in detecting PEx and may be more cost-effective than RC.  - Clinical/practical significance: HM can improve patient self-awareness and may reduce healthcare costs.  - Unexpected/contradictory findings: No significant differences in hospital inpatient bed days or overall health-related quality of life despite increased PEx detection. |
| **21** | Pilot Randomized Controlled Trial (RCT) | Adults with cystic fibrosis (awCF) with at least mild symptoms of depression or anxiety; sample size: 31 (CALM: 15, TAU: 16) | Reduce symptoms of depression and anxiety, improve coping and HrQOL in awCF | United States (specifically, Colorado) | Video call (using web camera and telehealth website) | - Primary findings: CALM group had lower mean scores for depression (medium ES) and anxiety (large ES) at post-intervention compared to TAU. Higher mean scores for coping self-efficacy (large ES) and HrQOL domains of Social Functioning (large ES) and Vitality (large ES).  - Effect sizes: Medium ES for depression, large ES for anxiety, coping, social functioning, and vitality.  - Secondary findings: Most treatment gains not sustained at 3-month follow-up; CALM was feasible, requiring < 12 min. for setup and scheduling; high acceptability and satisfaction.  - Authors' conclusions: CALM shows promise in reducing symptoms of depression and anxiety and improving coping and HrQOL; plans for further research via a multi-site RCT.  - Clinical significance: Potential low-cost and effective intervention for adults with cystic fibrosis; addresses gap in current mental health interventions.  - Practical significance: Feasible and acceptable telehealth delivery; increases access to psychological services while reducing travel burden and infection risk. |
| **22** | Observational study | 102 individuals with cystic fibrosis, median age 31, 60% male | Assess the impact of the shift to telehealth on respiratory microbiology monitoring during the COVID-19 pandemic | Canada | The telehealth format includes quarterly telehealth reviews with remote monitoring through sputum sample collection and analysis, where patients deposit samples at local laboratories for shipping to the center's laboratory. The exact method of telehealth review (phone call, video call, etc.) is not specified. | - Primary findings: Threefold reduction in respiratory samples collected during the pandemic; reduction in detection of new, clinically significant organisms.  - Effect sizes: Odds ratio for new significant organism identification during the pandemic was 0.21 (95% CI, 0.04-0.79; P = 0.016).  - Secondary findings: Reduced in-person contact challenges CF sputum microbiology monitoring; potential for missed or delayed pathogen detection.  - Authors' conclusions: Telehealth may lead to suboptimal monitoring; need for ongoing assessment and adjustments to remote care models.  - Clinical significance: Reduced monitoring may result in missed or delayed detection of pathogens like Pseudomonas. |
| **23** | One-group semi-experimental study with pre-and post-test methods | 45 cystic fibrosis patients under 7 years of age with parents willing to participate | Evaluation of mobile phone-based tele-monitoring for cystic fibrosis patients | Iran | Remote monitoring using a mobile phone-based customized Short Message Service (SMS) application | - Primary findings: Patient condition remained stable over three years with no significant changes in most parameters (P > 0.05); significant decrease in outpatient visits (P = 0.02).  - Effect sizes/statistical significance: Respiratory rate and arterial oxygen saturation levels stable (P = 0.544, P = 0.639).  - Secondary findings: Contradiction with Bella et al.'s findings on hospitalizations; 25% of subjects unable to continue remote evaluation.  - Authors' main conclusions: Telemedicine effective in maintaining patient condition and reducing outpatient visits; aligns with need for innovative care models.  - Clinical significance: Potential for telemedicine to improve quality of life and reduce care burden as CF population ages. |
| **24** | Prospective observational study | 110 adult CF patients (mean age 35 years, 54% female, 95% White) | Assess impact of interdisciplinary telemedicine on CF outcomes | United States | The telehealth format included video calls using WebEx, hybrid visits with in-person and video communication, and remote monitoring through home spirometry. Phone calls were used minimally. | - Primary findings: No difference in lung function or exacerbation rate; BMI increased from 25 to 26 kg/m² (P < .001); antibiotic use decreased from 316 to 124 episodes (P < .0001).  - Effect sizes/statistical significance: BMI increase (P < .001); antibiotic use decrease (P < .0001).  - Secondary findings: No patient deaths or lung transplantations; low COVID-19 incidence (2%); telemedicine accounted for 92% of clinical encounters.  - Authors' main conclusions: IDC-TM model is a feasible alternative to in-person care for maintaining lung function and BMI; social mitigation strategies reduced antibiotic use.  - Clinical significance: Effective monitoring and management of CF patients during the pandemic; protective role of social mitigation strategies.  - Unexpected findings: Low COVID-19 incidence among patients; significant decrease in antibiotic use. |
| **25** | Descriptive study/practical guide | Children with chronic lung conditions (CF, PCD, asthma); sample size: 391 spirometers sent out | Describe the setup and experience with a home spirometry service during the COVID-19 pandemic | United Kingdom | The telehealth format includes a combination of telephone consultations, video calls, and remote monitoring (home spirometry). | - Primary findings: Successful setup of a home spirometry service for children during the COVID-19 pandemic with 391 spirometers sent out over 9 months.  - Secondary findings: Challenges included incorrect patient data entry and the need for multiple video calls for setup and technique. Technical issues with initial tests required additional coaching.  - Authors' main conclusions: The rollout was successful and appreciated by most families. Home monitoring is expected to continue beyond the pandemic.  - Clinical significance: Home spirometry provided useful data to improve quality of care, as supported by a UKCFMA survey where 88% of centers found it useful.  - Practical significance: Ongoing data collection and patient feedback are crucial for service improvement. |
| **26** | Uncontrolled single-center pilot | 10 adults with cystic fibrosis (awCF) aged ≥18 years, with mild to moderate symptoms of depression and/or anxiety | To evaluate feasibility, usability, acceptability, and preliminary efficacy of eHealth CF-CBT | The Netherlands | The telehealth format used in the study is a combination of in-person sessions and virtual sessions (likely video calls) delivered through an internet-based platform (Minddistrict). | - Primary findings: eHealth CF-CBT is feasible, usable, and acceptable for awCF with mild to moderate symptoms of depression and anxiety.  - Effect sizes/statistical significance: Significant improvements in PHQ-9, GAD-7, PSS, and CFQ-R; ORS scores exceeded clinical reliable change cut-off.  - Secondary findings: Most participants did not require follow-up psychological treatment; potential as a first-line intervention.  - Authors' main conclusions: eHealth CF-CBT is promising for awCF; could act as a bridge to further treatment.  - Clinical significance: Reduces need for follow-up psychological treatment; useful for awCF with mild to moderate symptoms.  - Unexpected findings: No sex differences in primary outcomes despite predominantly female sample. |
| **27** | Prospective cohort study | Adults with cystic fibrosis (sample size: 31) | Understand patient satisfaction with Phlo app, its impact on clinical and economic outcomes, and its acceptability within the healthcare team | United States | Remote monitoring through a mobile application (Phlo) with features for tracking daily regimen activities, medication management, and direct communication with healthcare providers via a web-based dashboard. | - Primary findings: Most patients (67%) reported improved confidence and motivation in managing their regimen; the app provided a single location for managing their routine.  - Effect sizes/statistical significance: No significant change in QoL domains or patient activation measures; trend towards reduction in hospital admissions and outpatient visits (p = 0.17 and p = 0.55, respectively).  - Secondary findings: Increase in patients rating themselves as "Excellent" in managing their CF treatment routine; median medication adherence score of 57.  - Authors' main conclusions: The app is feasible for integration into healthcare systems; it may be beneficial for patient satisfaction and particularly for those struggling with adherence.  - Clinical/practical significance: Potential for improving patient care through better management and communication; need for further research on long-term benefits.  - Unexpected/contradictory findings: No significant changes in QoL or patient activation measures despite positive patient feedback. |
| **28** | Cross-sectional survey | 85 responses from CF multidisciplinary health professionals and center directors, representing 15 of 23 CF centers in Australia | Describe clinician experiences and attitudes towards telehealth in CF care | Australia | Video calls and remote monitoring (including home spirometry) | - Primary findings: Telehealth is widely used and accepted by clinicians, but concerns exist about its impact on health outcomes due to missed core assessments.  - Effect sizes: 91.8% of clinicians find telehealth acceptable; 81.2% are comfortable integrating it into future care; 64.1% consider telehealth clinics worse than face-to-face; 57.5% report worse quality of care.  - Secondary findings: Home spirometry availability is variable; technical issues are common; there is a need for guidelines to ensure standard care.  - Authors' conclusions: Telehealth is feasible and acceptable, but guidelines are needed to address variability and ensure core assessments are not missed.  - Clinical significance: The need for guidelines to ensure that telehealth does not compromise the standard of care.  - Unexpected findings: The contradiction between acceptability and concerns about health outcomes. |
| **29** | Qualitative | Multidisciplinary team members at a large paediatric CF centre | Assessing the effectiveness and challenges of telemedicine in cystic fibrosis care | United Kingdom | The telehealth format includes a combination of phone calls, video calls (using AttendAnywhere), and remote monitoring through devices such as hand-held spirometers, weighing scales, stadiometers, and blood testing systems. Additionally, it involves the collection and analysis of airway samples and the delivery of acute medicines to patients' homes. | - Primary findings: Telemedicine is beneficial but face-to-face clinics are still necessary for certain assessments and patient care.  - Secondary findings: Increased frequency of monitoring, reduced travel costs and stress, improved communication within the multidisciplinary team.  - Authors' main conclusions: Telemedicine has advantages but requires further optimization and evidence to support widespread adoption.  - Clinical significance: Improves patient convenience and reduces infection risks, but face-to-face interactions are necessary for certain assessments. |
| **30** | Real-life observational study | 174 patients with cystic fibrosis (74 children, 43 teenagers, 57 adults) | Assess and compare adherence, reliability, and variability of home spirometry between children, teenagers, and adults with CF | France | Remote monitoring using a Bluetooth-enabled home spirometry device (MIR-Spirobank ® Smart) with automatic data transmission to a dedicated platform. | - Primary findings: Home spirometry was used at least once a week by 64.1% of patients, with higher adherence in children and teenagers. Reliability to conventional spirometry was good (e.g., FEV1: r = 0.91, p < 0.01), with low variability (FEV1 coefficient of variation = 11.5%).  - Effect sizes/statistical significance: FEV1 correlation coefficient (r = 0.91), coefficient of variation (11.5%).  - Secondary findings: Better adherence in children, better reliability and lower variability in adults and teenagers.  - Authors' main conclusions: Home spirometry is a reliable tool for multi-weekly lung function follow-up, potentially useful for early exacerbation detection.  - Clinical significance: Could improve disease management by early detection of exacerbations.  - Limitations: Potential biases due to patient data transmission, short-term follow-up period. |
| **31** | Cohort study | 28,132 participants with cystic fibrosis (13,913 children, 14,219 adults) | Quantify the extent to which individuals with CF received recommended care components in 2019 versus 2020 and identify factors associated with telehealth use | United States | The telehealth format included remote monitoring (such as home spirometry) and likely video or phone calls for consultations and screenings, although the exact format is not specified. | - Primary findings: Fewer individuals with CF met care standards in 2020 compared to 2019 due to the pandemic. Telehealth was widely used but did not equate to improved adherence to all care aspects.  - Effect sizes and statistical significance: Demographic and socioeconomic factors were significantly associated with telehealth use and care adherence. For example, minority race/ethnicity and lower socioeconomic status were linked to lower telehealth use in children.  - Secondary findings: Prior adherence to care recommendations in 2019 was a strong predictor of telehealth use and meeting care standards in 2020.  - Authors' main conclusions: Telehealth maintained care levels for those already adherent to guidelines before the pandemic, but barriers to telehealth access may reflect broader challenges in accessing care.  - Clinical significance: The study highlights the need for future research on social risk factors and the integration of telehealth into chronic care models. |
| **32** | Randomized controlled trial | Consecutive adult CF patients (n=32), age ≥18 years, bilateral lung transplantation, affected by cystic fibrosis | Evaluate the use of telemonitoring in the surveillance program for CF patients after lung transplantation | Italy | Remote monitoring (home spirometry and pulse oximetry), online video consultations | - Primary findings: No statistically significant difference in allograft dysfunction incidence, diagnosis timing, or quality of life between telemonitoring and control groups.  - Effect sizes/statistical significance: p = 0.137 for incidence of allograft dysfunction.  - Secondary findings: Telemonitoring data consistent with hospital data; adherence decreased over time; high patient satisfaction.  - Authors' main conclusions: Telemonitoring can lead to earlier diagnosis of graft dysfunction; reduces financial and travel burdens.  - Clinical significance: Telemonitoring is a reliable tool for remote monitoring; can improve patient care by reducing hospital visits.  - Unexpected findings: Decrease in adherence over time. |
| **33** | Pragmatic feasibility study | Children with cystic fibrosis aged ≥ 6 years; sample size = 67 | Assess differences between home and clinic spirometry readings and variability of home spirometry | United Kingdom, Canada | Remote monitoring (home spirometry) | - Primary findings: Unsupervised home spirometry readings are significantly lower than supervised clinic readings, with a mean bias of 6.5% and wide limits of agreement (-9.6% to +22.7%).  - Effect sizes: 76.2% of participants recorded lower results at home; variability of home measurements during clinical stability was around 10%.  - Secondary findings: Difference in percent predicted values was greater in younger children; underread by the handheld device could lead to unnecessary treatment.  - Authors' main conclusions: Home spirometry cannot be used interchangeably with clinic spirometry; precautions such as supervision and direct comparison with clinic measurements are necessary.  - Clinical significance: Findings suggest home spirometry may not be reliable for monitoring lung function in children with cystic fibrosis, potentially leading to incorrect treatment decisions. |
| **34** | Mixed methods (quantitative and qualitative) | 250 families with infants undergoing cystic fibrosis newborn screening | To assess the feasibility and effectiveness of genetic counseling via telemedicine for cystic fibrosis newborn screening | United States | Video call (HIPAA compliant telemedicine setup) and phone call (for qualitative interviews) | - Primary findings: High satisfaction with telemedicine GC (4.77/5), improved understanding of CF genetics (100% recognized child as carrier, 87.7% understood both parents could be carriers).  - Effect sizes/statistical significance: p = .023 for child carrier status, p < .0001 for both parents being carriers, p < .0001 for increase in sweat tests.  - Secondary findings: Significant increase in sweat tests completed (49% to 80%), qualitative data showed GC was beneficial and reassuring.  - Authors' main conclusions: Telemedicine GC is feasible, improves knowledge, and can improve health equity.  - Clinical significance: Improves access to care, especially for underserved areas, and enhances understanding of CF genetics.  - Unexpected findings: No significant difference in understanding increased risk of having a child with CF. |
| **35** | Pilot study | Adult patients with cystic fibrosis (CF) who are lung transplant candidates; sample size = 11; demographics = 45% female, age 33 ± 7 years, FEV1 27 ± 5% predicted | To assess adherence to a home-based PR program using a fitness application and self-selected activity | United States | The telehealth format used in this study was a combination of remote monitoring (using a fitness application to track heart rate and exercise time) and asynchronous communication (via text messages and emails). | - Primary findings: Patients with severe CF showed better adherence to home-based PR compared to hospital-based PR (19 ± 12 sessions vs. 9 ± 4 sessions, p = :03). Fifty percent completed ≥24 sessions in 12 weeks, compared to 0% in the hospital-based group (p = :03).  - Effect sizes/statistical significance: p = :03 for both adherence and completion rates.  - Secondary findings: No adverse events during exercise. Completers showed a clinically meaningful lower decline in 6MWD (-7 ± 15 meters vs. -86 ± 108 meters).  - Authors' main conclusions: Home-based PR using a fitness application and self-selected activity is a viable solution for telerehabilitation in severe CF, particularly relevant during the COVID-19 pandemic.  - Clinical significance: The program is safe and effective, addressing limitations in traditional PR programs. |
| **36** | Qualitative | 20 patients completed the study; total of 36 patients (22 adults, 14 adolescents aged >12 years) | Identify contributions and conditions of home monitoring to detect PEx early and treat it | France | The telehealth format in this study included remote monitoring using connected devices (such as home spirometry), phone calls, video calls, and email communication for data sharing and consultations. | - Primary findings: Three main categories emerged - task technology fit, patient empowerment through technology, and use of technology.  - Effect sizes/statistical significance: Not mentioned.  - Secondary findings: Connected devices increase patient empowerment and enhance healthcare provider understanding.  - Authors' main conclusions: Connected devices can empower patients with CF by increasing their ability to manage their health.  - Clinical/practical significance: The study provides a framework for integrating connected devices into CF management.  - Unexpected/contradictory findings: Not mentioned. |
| **37** | Qualitative study using semistructured interviews and thematic analysis | 23 adults with cystic fibrosis, aged 21 to 60 years, 14 females | To understand the perspectives of adults with CF regarding telehealth exercise interventions | Australia | The telehealth format includes video conferencing for live group online exercise classes and prerecorded videos, as well as remote monitoring using fitness tracking apps and smartwatches. | - Primary findings: The study identifies three major themes - personalizing components of an exercise program, maintaining connections, and monitoring health and exercise.  - Secondary findings: Participants value tailoring programs to individual needs, maintaining connections with peers and healthcare teams, and monitoring progress.  - Authors' main conclusions: Telehealth exercise interventions should be tailored to individual needs, include opportunities for peer support and specialist input, and provide methods for monitoring progress.  - Clinical significance: The findings can inform the design of more effective and engaging telehealth exercise programs for adults with CF.  - No unexpected or contradictory findings are reported. |
| **38** | Retrospective chart review | 214 patients with cystic fibrosis; median age 11 years; 58% male | Assess impact of telehealth clinics on CF outcomes (lung function, microbiology, nutritional status, admissions) | Australia | The telehealth format included video calls for clinic sessions, remote monitoring through home spirometry, and remote microbiological sampling with equipment and instructional support. | - Primary findings: Median decline in FEV1 by 5.4% from pre-lockdown to post-lockdown; 31.9% of patients experienced a decline of more than 10%.  - Effect sizes: Median change in FEV1 was -5.4%; 31.9% of patients had a decline of >10%.  - Secondary findings: No significant changes in microbiology or anthropometry; reduction in microbiology samples; weight and BMI remained stable; reduction in hospital admissions.  - Authors' main conclusions: Telehealth should complement, not replace, in-person care; need for further evaluation to safely integrate telehealth into routine CF care.  - Clinical significance: Importance of improving telehealth and maintaining face-to-face reviews for pediatric CF patients.  - Unexpected findings: Home samples were sufficient for detecting P. aeruginosa despite reduced microbiology samples. |
| **39** | Observational prospective study | 74 adult patients with cystic fibrosis; mean age 37 years; 50% male; recruited from Royal Prince Alfred Hospital, Sydney, Australia | Evaluate patient experience of multidisciplinary outpatient care through telehealth compared with face-to-face care | Australia | The telehealth format used in the study included videoconferencing and remote monitoring through home spirometry. | - Primary findings: No differences in patient ratings of healthcare teams, general and mental health, or confidence in handling treatment plans between face-to-face and virtual care models. No between-group differences in CFQ-R scores.  - Effect sizes/statistical significance: Travel time and costs significantly reduced for regional participants (4 h, AU$108 per clinic; P < 0.05).  - Secondary findings: High preference for hybrid model (93%); virtual care is a feasible alternative to traditional face-to-face clinics.  - Authors' main conclusions: Virtual care does not compromise quality of life or patient experience; it offers cost savings but its impact on objective health outcomes is unclear.  - Clinical significance: Virtual care is a viable option for cystic fibrosis patients, particularly those in regional areas.  - Unexpected findings: No significant differences in quality of life measures between face-to-face and virtual care. |
| **40** | Case study | 21-month-old female with cystic fibrosis and multiple food allergies | Provide evidence-based behavioral and nutrition treatment via telehealth | United States | Phone calls, faxing, and emailing | - Primary findings: Significant increase in energy intake from 1,212 kcal/day to 1,715 kcal/day; exceeded recommended 120% RDA for children with CF.  - Effect size: 503 kcal/day increase in energy intake.  - Secondary findings: Increase in fat intake; durable impact with maintenance of gains 18 months post-treatment.  - Authors' main conclusions: Telehealth-delivered behavioral and nutrition intervention is effective and accessible for families with barriers to traditional care.  - Clinical significance: Exceeded clinical benchmarks for energy intake; durable impact.  - Practical significance: Makes interventions accessible to families who cannot travel to clinics. |
| **41** | Randomized controlled trial (RCT) | 16 patients with cystic fibrosis, terminally ill; 11 completed baseline assessment, 7 completed the study | Assess feasibility of home telemedicine for patients with cystic fibrosis awaiting transplantation | United Kingdom | Video call (videoconferencing) and remote monitoring (home spirometry, pulse-oximeter, clinical thermometers) | - Primary findings: No significant differences in quality of life, anxiety, depression, hospital admissions, or clinic attendances between telemedicine and control groups.  - Significant improvement in perception of body image for the telemedicine group (P = 0.02).  - High patient satisfaction with telemedicine.  - Telemedicine can enhance support and potentially reduce outpatient clinic attendances.  - Limitations: Small sample size, impact of patient deaths and transplants.  - Conclusion: Further studies with larger sample sizes are needed. |
| **42** | Case study | 19-year-old female with cystic fibrosis and severe respiratory failure | To demonstrate the effectiveness of telemedicine in the follow-up of lung transplantation in a patient with cystic fibrosis | Italy | Remote monitoring (home spirometry using Oxitel and Spirotel, and Intel Health Guide), phone calls | - Primary findings: Remote monitoring identified early episodes of pulmonary relapse and graft-versus-host reaction before symptoms appeared, allowing for effective intervention and recovery.  - Secondary findings: Telemedicine detected a significant drop in FEV1 (-13%) post-transplant, leading to timely hospitalization and treatment. Clinical stability was facilitated by telemedicine pre-transplant.  - Authors' main conclusions: Telemonitoring enabled early detection of complications, preventing permanent lung damage and facilitating recovery to near pre-transplant lung function levels.  - Clinical significance: Telemedicine is crucial for early detection and management of post-transplant complications, improving patient outcomes. |
| **43** | Descriptive study with a cross-sectional survey | 60 adult patients with cystic fibrosis | To describe the transition to telehealth and assess patient experience | United States (specifically, Virginia) | The telehealth format includes a combination of video calls (via Zoom), phone calls, and remote monitoring (using home spirometry). | - Primary findings: Positive reception towards telehealth with 100% of patients believing it improves access to care and 90% feeling it does not negatively affect provider relationships.  - Secondary findings: Challenges in obtaining PFTs, cultures, labs, and imaging data; issues with access to technology for virtual visits.  - Authors' conclusions: Telehealth is well-received but needs improvement in efficiency and accessibility.  - Clinical significance: Potential for improved access to care and patient satisfaction, with areas for improvement in implementation. |
| **44** | Mixed-methods randomized controlled feasibility study | 40 cystic fibrosis patients aged > 6 years | To assess the feasibility and acceptability of internet-delivered Tai Chi compared to in-person tuition | United Kingdom | video call (Skype) | - Primary findings: Both face-to-face and internet delivery of Tai Chi lessons were equally well received and perceived as beneficial by patients with cystic fibrosis.  - Effect sizes/statistical significance: Quantitative outcomes showed small, but not significant improvements for breathing, sleep, and mindfulness.  - Secondary findings: Internet delivery was convenient and engaged patients who were geographically isolated; technical difficulties were reported by some participants.  - Authors' main conclusions: Online taught Tai Chi is possible and comparable to in-person tuition, offering convenience and engagement.  - Clinical significance: Tai Chi can improve respiratory function and overall health, especially during the COVID-19 pandemic for immunocompromised individuals.  - Unexpected findings: None explicitly mentioned. |
| **45** | Pilot study | Ten children and adolescents with cystic fibrosis (CF), aged 8-20 years | Evaluate the feasibility of a live-streamed platform for delivering supervised and interactive group exercise sessions to CF children | United States | Video call (VSee telemedicine platform) and remote monitoring (heart rate readings) | - Primary findings: High attendance rate (85% of sessions attended), successful engagement in moderate-vigorous physical activity (21.1 ± 6.9 min), high user satisfaction (SUS score: 90.8 out of 100).  - Effect sizes/statistical significance: No significant changes in peak VO2 or pulmonary function tests.  - Secondary findings: Participants enjoyed the program and found it easy to use; tele-exercise mitigates cross-infection risk.  - Authors' main conclusions: Tele-exercise is a promising tool for promoting exercise in children with CF, offering flexibility and accessibility.  - Clinical/practical significance: Provides a safe and engaging way to promote physical activity in children with CF.  - Unexpected/contradictory findings: No significant improvements in peak VO2 or pulmonary function tests. |
| **46** | Single group study | Adults with cystic fibrosis (CF) living in rural and remote Western Australia; sample size: 23 initially recruited, 21 included in final analysis | Evaluate uptake and satisfaction with telehealth clinics and their impact on health outcomes | Australia (specifically, Western Australia) | Video call using Polycom HDX Õ series equipment, with in-person measurements at regional hospitals | - Primary findings: Increased clinic visits from 46 to 100, with 66 via telehealth; high satisfaction with telehealth; increased intravenous antibiotic days (IRR 2.3, p = 0.03) and hospital admission days (IRR 3.7, p = 0.01); improvement in vitality domain of CFQ-R (p < 0.05).  - Effect sizes/statistical significance: p < 0.001 for increased clinic visits; p = 0.03 for IVAB days; p = 0.01 for hospital admission days; p < 0.05 for vitality domain improvement.  - Secondary findings: Trend towards improvement in weight domain of CFQ-R; weight gain in 67% of participants.  - Authors' main conclusions: Telehealth increases access to care and may improve long-term outcomes; it is a feasible method for delivering CF care in rural and remote areas.  - Clinical significance: Increased surveillance and treatment of exacerbations may lead to improved health outcomes.  - Unexpected findings: Increased hospital admissions and antibiotic use, attributed to increased surveillance and detection of exacerbations. |
| **47** | Feasibility study | Nine patients with cystic fibrosis (three female, six male, 30.9 years old) | Assess the feasibility and acceptability of using video-calling technology for exercise training in patients with cystic fibrosis | United Kingdom | Video calls via Skype | - Primary findings: The use of Skype for exercise training in cystic fibrosis patients is technologically feasible and acceptable.  - Effect sizes/statistical significance: Mean compliance rate of 68%, mean session duration of 20 minutes, 25% of calls experienced technical issues.  - Secondary findings: No significant changes in anthropometric, pulmonary, physical activity, or quality of life variables.  - Authors' main conclusions: Skype is a feasible and acceptable platform for delivering exercise interventions to cystic fibrosis patients, with potential to overcome geographical barriers and reduce cross-infection risks.  - Clinical significance: Implications for clinical practice in engaging patients remotely and reducing healthcare delivery costs.  - Unexpected findings: No significant changes in health outcomes despite high participant satisfaction. |
| **48** | Prospective interventional study | Adolescents with cystic fibrosis (CF) aged 12-21 years; sample size = 39; mean age = 15.89 years; 54% female | Evaluate the impact of frequent home PFT monitoring on medication adherence | United States | The telehealth format used in this study included remote monitoring (home spirometry) and phone calls, with some participants also using text messages as an alternative to phone calls. | - Primary findings: Frequent home spirometry significantly improved medication adherence (MPR increased from 60% to 65%, P = 0.04).  - Effect sizes/statistical significance: MPR increase significant at P = 0.04; no significant change in treatment burden (P = 0.14).  - Secondary findings: No change in pulmonary exacerbations or FEV1% decline; significant decline in BMI percentile (P = 0.009).  - Authors' conclusions: Home spirometry is feasible and improves adherence without affecting treatment burden.  - Clinical significance: Can be implemented in clinical practice to improve adherence and health outcomes.  - Unexpected findings: No change in pulmonary exacerbations or FEV1% decline despite improved adherence. |
| **49** | Single-centre pilot study | 18 CF patients older than eight years of age; 9 in intervention group, 9 in control group | Assess feasibility and acceptability of telehealth using WhatsApp and Skype | Israel | Skype video chats and WhatsApp text messaging | - Primary findings: No significant differences in CFQ-R scores, knowledge, adherence, or patient satisfaction between intervention and control groups.  - Effect sizes/statistical significance: Significant increase in reported adherence to hypertonic saline in both groups (p = 0.015).  - Secondary findings: Telehealth approach using Skype and WhatsApp was feasible and acceptable.  - Authors' main conclusions: Telehealth approach is promising but requires further investigation in a larger study.  - Clinical significance: Potential for improving communication and adherence in CF patients.  - Limitations: Small sample size limited ability to detect significant differences. |
| **50** | Qualitative | 14 participants (7 young people aged 16-24 with long-term health conditions, 7 clinicians) | Assess face and content validity of PAM and PHBQ for digital consulting | United Kingdom (England and Wales) | The telehealth format includes email, text messaging, and Skype (video calls). | - Primary findings: PAM and PHBQ have reasonable face and content validity for assessing digital consulting outcomes.  - Secondary findings: Young people found most PAM items relevant but struggled to separate digital from face-to-face experiences; clinicians found PAM items relevant and provided examples with ease; PHBQ items were relevant to digital consulting.  - Authors' main conclusions: PAM 13 is the best-evidenced PROM for evaluating digital consulting's impact on patient activation behaviors; revisions are needed to differentiate between digital and face-to-face consultations.  - Clinical significance: These PROMs are useful for evaluating digital consulting outcomes, despite potential limitations. |
| **51** | Cohort/Observational study | 60 children with cystic fibrosis, mean age 13.25 years | Evaluate safety and effectiveness of home care program and regular telephone contact | Greece | Phone call | - Primary findings: Significant improvements in quality of life, treatment adherence, and lung function (FEV1) among children with cystic fibrosis.  - Effect sizes and statistical significance: QoL improved from 39.88 to 43.44 (p < 0.001), FEV1 improved by 8.00 (p = 0.001), hospitalization days decreased by 1.65 (p = 0.02), and costs decreased by €431.00 (p = 0.005).  - Secondary findings: Decrease in hospitalization days and costs.  - Authors' main conclusions: Home care and telephone communication are effective in improving outcomes for children with CF.  - Clinical significance: Regular telephone communication recommended for improving treatment adherence, especially for distant patients.  - Practical significance: Telephone calls enable comprehensive monitoring and treatment adjustment, potentially reducing emergency visits and hospital admissions. |
| **52** | Observational | 20 patients (13 participated in virtual medication tour), median age 29 years, mix of male and female | Describe the uptake and impact of pharmacist-led virtual medication tours during telehealth visits | United States | Video call using webcam, cellular phone camera, or other device for visual communication | - Primary findings: Virtual medication tours can be successfully incorporated into telehealth visits and are accepted by a majority of patients.  - Effect sizes: 13 out of 20 patients participated; 80% resolution of missing medication list information.  - Secondary findings: Three out of four patients with children under 12 had medications stored in accessible locations; most patients stored medications appropriately but needed education on poison prevention.  - Authors' main conclusions: Virtual medication tours are feasible and beneficial; they can identify opportunities for intervention and improve medication storage practices.  - Clinical significance: Easy integration into telehealth visits without additional time or resources; highlights need for education on poison prevention.  - Limitations: Small sample size; lack of comparator group. |
| **53** | Randomized, retrospective case-control study | Sample size - IN: 44, OUT: 110; Demographics: Patients with cystic fibrosis | To assess reduction in Pseudomonas Aeruginosa colonization and hospital admissions with telemonitoring | Italy | Remote monitoring (home spirometry using MIR Spirotel 2) | - Primary findings: Telemonitored patients had fewer new Pseudomonas Aeruginosa infections; control group had a significant decrease in respiratory function.  - Effect sizes/statistical significance: Significant increase in outpatient admissions (P = 0.0019 and P <0.001); significant decrease in day hospital admissions (P <0.001); significant increase in Pseudomonas infections in control group (P = 0.0212).  - Secondary findings: No significant changes in colonizations in the telemonitored group; significant increase in Pseudomonas infections in the control group.  - Authors' main conclusions: Home telemonitoring is useful for monitoring respiratory function, especially for distant patients; it allows for rapid diagnosis and treatment, reduces hospital visits, and improves quality of life.  - Clinical significance: Reduces risk of acquiring multi-resistant microbes; improves patient adherence.  - Unexpected findings: Significant decrease in respiratory function in the control group; significant increase in Pseudomonas infections in the control group. |
| **54** | Cross-sectional web-based survey | 80 clinicians from medium and large CF programs in the Northeast, Midwest, South, and Pacific-Northwest; follow-up survey completed by 63 clinicians | Characterize telehealth usage patterns, attitudes, and preferences amongst clinicians for future telehealth care | United States | The telehealth format primarily used audio plus video connectivity, with Zoom as the most commonly used platform. It included an interdisciplinary asynchronous format where multiple clinicians evaluated patients sequentially. There was also interest in remote monitoring technologies like home spirometry for lung function assessment. | - Primary findings: Clinicians found telehealth highly satisfying, efficient, and it improved the clinician-patient relationship.  - Effect sizes/statistical significance: Not explicitly mentioned.  - Secondary findings: Preference for interdisciplinary care model, particularly asynchronous; increase in perceived barriers over time; technological limitations a concern for pediatric clinicians.  - Authors' main conclusions: Telehealth is well accepted and useful for enhancing interdisciplinary CF healthcare delivery.  - Clinical/practical significance: Telehealth is a viable option for CF care, with potential for technology innovation.  - Unexpected/contradictory findings: Increase in perceived barriers over time, particularly technological limitations among pediatric clinicians. |
| **55** | Feasibility study | 63 adult cystic fibrosis patients | Develop a standardized and sustainable process for transitioning to telemedicine clinic | United States | Video conferencing via WebEx, telephone calls for those without technology access, and remote monitoring through home spirometry. | - Primary findings: Telemedicine implementation was feasible for clinically stable cystic fibrosis patients during the COVID-19 pandemic, with 60% of scheduled patients being seen through telemedicine.  - Secondary findings: FEV1 was stable in all patients for whom spirometry results were available; connectivity issues were a challenge; one patient was identified with acute needs and directed to the Emergency Department.  - Authors' main conclusions: Telemedicine is a feasible and sustainable solution for multidisciplinary care during crises like the COVID-19 pandemic.  - Clinical significance: Telemedicine can maintain healthcare functionality and offset financial losses during social distancing measures.  - Practical significance: Patient buy-in and technology access are limiting factors for telemedicine adoption. |
| **56** | Mixed-methods (qualitative research and pilot RCT) | Adults with cystic fibrosis, aged 16 or older, from the UK (sample size: 18 for qualitative interviews, 32 for pilot RCT) | Develop an intervention to increase adherence to nebuliser treatments in adults with CF, focusing on reducing effort and treatment burden | United Kingdom | Remote monitoring (digital platform collecting and displaying nebuliser adherence data), digital communication (personalized notifications and reminders via mobile app) | - Primary findings: The intervention includes components such as understanding treatment, monitoring adherence, setting treatment goals and feedback, developing confidence in adherence, creating treatment plans, and solving problems related to adherence.  - Secondary findings: The intervention is complex and tailored to meet individual patient needs; it is usable and acceptable to patients and clinicians.  - Authors' main conclusions: The intervention addresses multiple barriers to adherence and is designed to be equitable and effective for a range of patients.  - Clinical significance: The intervention is currently being tested in a randomized controlled trial, indicating its potential for improving adherence in CF patients.  - Practical significance: The intervention is designed to be personalized and tailored to individual patient needs, making it adaptable for different barriers to adherence. |
| **57** | Cross-sectional study | Patients with cystic fibrosis (CF) and inflammatory bowel disease (IBD), sample size of 654 patients from Bergamo and Milano | Evaluate psychological effects on patients with chronic respiratory conditions and IBD through analysis of spontaneous contacts | Italy (specifically in Lombardia, with locations in Milano and Bergamo) | Phone calls, video calls, emails | - Primary findings: 1,816 contacts collected; 88.7% of Milano patients contacted via email; Bergamo had more empathy and positive feedback.  - Effect sizes/statistical significance: P< .001 for email preference in Milano; P< .001 for empathy and P = .003 for positive feedback in Bergamo.  - Secondary findings: Context drove patient reactions; Milano focused on information requests, Bergamo on positive feedback.  - Authors' conclusions: Investing in health and cohesive teams supports patient resilience.  - Clinical significance: Remote support by multidisciplinary teams is crucial for coping with pandemic psychological impacts. |
| **58** | Cross-sectional analysis | 424 PwCF and 286 CF care programs; 90% White, 6% Hispanic/Latino, 2% Black | Understand access to and experiences with telehealth among diverse racial/ethnic and socioeconomic backgrounds | United States | Phone call and video call (with or without a video component) | - Primary findings: Racial/ethnic minorities were less likely to use telehealth (p = .015), with pronounced differences in the Hispanic/Latino population (p < .01). Financial difficulties were associated with perceived difficulties in using telehealth (p = .018) and lower satisfaction with care (p = .010, p = .020).  - Effect sizes/statistical significance: p-values indicate statistical significance for racial/ethnic disparities and financial difficulties.  - Secondary findings: No differences in telehealth use by health insurance type; programs identified technology, language barriers, and home conditions as barriers.  - Authors' main conclusions: Further studies are needed to ensure telehealth is part of an equitable care model for diverse backgrounds.  - Clinical significance: Highlights disparities in telehealth access and use, impacting health outcomes for cystic fibrosis patients from diverse backgrounds. |
| **59** | Program evaluation | 78 lung transplant candidates, 33 recipients; mean age 58 years; 51% males; 52% with interstitial lung disease; 31% with chronic obstructive pulmonary disease | Evaluate usage, satisfaction, and functional outcomes of telerehabilitation for lung transplant candidates and recipients during the COVID-19 pandemic | Canada | The telehealth format used in this study includes a combination of phone calls, video calls, asynchronous texting, and remote monitoring. The platform features include online patient education, biometric data monitoring, and secure videoconferencing. Physiotherapists used phone calls, video calls, and texting to provide support and monitor patients, with a preference for texting over traditional phone calls. | - Primary findings: Increased exercise session duration for candidates, increased treadmill speed for recipients, improved physical activity levels among candidates.  - Effect sizes/statistical significance: P=.002 for increased exercise duration, P=.003 for increased treadmill speed, P=.02 for improved physical activity.  - Secondary findings: Decrease in pretransplant 6-minute walk distance, no change in SPPB results, increased quadriceps weight posttransplant.  - Authors' main conclusions: Telerehabilitation is feasible despite challenges, functional outcomes were lower compared to traditional rehabilitation.  - Clinical significance: Telerehabilitation can maintain physical activity during the pandemic, but improvements were lower than traditional rehabilitation.  - Unexpected findings: Decrease in pretransplant 6-minute walk distance, underprescription of exercise intensity due to lack of on-site assessments. |
| **60** | Retrospective analysis | Lung transplant recipients; sample size: 53 for video consultations, 51 for on-site visits | Analyze clinical impact, technical feasibility, and patient satisfaction of video consultations | Germany | Video call using Sprechstunde.online, text chat platform, data file exchange tool, and remote monitoring using home spirometry and pulse oximetry devices. | - Primary findings: 75 VCs were performed for 53 patients, with 77% of physician-patient contacts occurring through VC by the end of the study period. Physician-patient consultations were reduced by 47% compared to 2019.  - Effect sizes: 62% of VCs resulted in concrete clinical decisions; 36% led to medication changes; 19% led to further diagnostic steps.  - Secondary findings: High patient satisfaction; technical issues in 15% of VCs; older patients faced barriers to telemedicine.  - Authors' main conclusions: VC is effective in reducing OSVs and SARS-CoV-2 exposure while maintaining access to specialist care for chronic illnesses.  - Clinical significance: VC can preserve access to specialist care while reducing SARS-CoV-2 exposure.  - Unexpected findings: Older patients faced barriers to telemedicine; technical issues did not significantly hinder the program's success. |
| **61** | Quality improvement project using PDSA strategy | Approximately 250 adult cystic fibrosis patients aged 18 years and older | Evaluate patient and staff perceptions of telemedicine clinic structure | United States (California) | Video calls, telephone calls, and remote monitoring (home spirometry and home sputum collection kits) | - Primary findings: Video visits were convenient, efficacious, and comparable to in-person visits.  - Effect sizes/statistical significance: 92% of patients reported ease of access, 94% had questions addressed; 52% of patients and most staff found video visits comparable to in-person visits.  - Secondary findings: Positive aspects include convenience, efficiency, and safety; negative aspects include communication issues, technological problems, and lack of diagnostic data.  - Authors' main conclusions: Telemedicine preserved CF care delivery during the pandemic; further research is needed to assess safety and effectiveness.  - Clinical/practical significance: Telemedicine is a viable alternative for CF care, especially during pandemics; efforts are needed to address limitations like diagnostic data collection. |
| **62** | Prospective cohort study | 59 children for device validation (26 male, mean age 12.3 years, SD 3.3); 80 children for home spirometry (41 male, mean age 12.3 years, SD 3.3) | Determine accuracy of personal ultrasonic spirometer and assess ability of children to perform spirometry at home during telehealth consultations | Australia | Remote monitoring (home spirometry) with supervision likely through video calls | - Primary findings: High ICC values for FEV1 and FVC (0.991 and 0.989, respectively) indicate excellent reliability between devices. Wide limits of agreement (-0.22 to 0.24 L for FEV1 and -0.30 to 0.33 L for FVC) suggest caution when using devices interchangeably.  - Effect sizes/statistical significance: High ICC values and wide limits of agreement.  - Secondary findings: 89% success rate for supervised telehealth spirometry sessions; significant differences in measurements for a subset of children.  - Authors' main conclusions: The personal ultrasonic spirometer is a valid alternative to laboratory equipment but should not be used interchangeably due to wide limits of agreement.  - Clinical significance: Suitable for home use during supervised telehealth consultations; caution needed when monitoring lung function over time.  - Unexpected/contradictory findings: Wide limits of agreement despite high ICC values. |
| **63** | Randomized controlled trial (RCT) | Children with cystic fibrosis aged 6-17 years; sample size = 61 | Determine whether remote supervision by a physiologist improves technical quality and failure rate of home spirometry | United Kingdom | The telehealth format used in this study includes video calls (via Zoom), remote monitoring (using the NuvoAir Home platform), and telephone calls for follow-up and intervention. | - Primary findings: Supervised home spirometry significantly improves technical quality compared to unsupervised spirometry (89% vs. 74% achieving QF A; p = <0.001).  - Secondary findings: No significant difference in achieving QF A-C between supervised and unsupervised groups (99% vs. 95%; p = 0.102).  - Authors' conclusions: Remote supervision by a physiologist is ideal for quality-assured remote testing, but acceptable results can be obtained without supervision if proper training and monitoring are provided.  - Clinical significance: Remote supervision improves technical quality, which is crucial for accurate lung function monitoring in children with cystic fibrosis.  - Practical significance: Positive feedback from families and patients indicates acceptability and usability of home spirometry.  - Unexpected findings: All significant declines in spirometry results were due to clinical rather than technical reasons. |
| **64** | Single-center, retrospective study | 119 children with cystic fibrosis or interstitial lung disease | Evaluate the efficiency of telephone visits in determining pulmonary exacerbations and hospitalization rates | Turkey | Phone call | The study found that telephone visits were effective in diagnosing acute pulmonary exacerbations in 10% of children with CF and ILD during the COVID-19 pandemic, with 5.8% requiring hospitalization. The median APEx frequency did not differ significantly from the previous year, suggesting no impact of the pandemic on exacerbation rates. Telemedicine reduced COVID-19 exposure risk but had limitations due to reliance on patient-reported symptoms. The authors concluded that telemedicine is beneficial during pandemics but should not replace traditional medical examinations. |
| **65** | Multicentre randomised controlled trial (RCT) with assessor blinding and qualitative evaluation | People with CF aged 12-35 years; sample size = 107 participants | Investigate the effect of ActivOnline on promoting physical activity in young people with CF | Australia | Remote monitoring via a web-based application (ActivOnline) accessible from any internet-enabled device, with email reminders for engagement. | - Primary findings: No statistically significant difference in MVPA between intervention and control groups; low uptake of the intervention.  - Effect sizes/statistical significance: Mean difference (95% CI) -14 mins (-45 to 16) for MVPA.  - Secondary findings: High baseline physical activity levels; low engagement with the intervention.  - Authors' main conclusions: The web-based application was no better than usual care in promoting physical activity; need to determine best strategies for increasing physical activity in CF patients.  - Clinical/practical significance: Highlights challenges in using digital interventions for physical activity promotion in CF patients; need for alternative strategies.  - Unexpected/contradictory findings: Low engagement with the intervention despite previous positive feedback on similar technology-based interventions. |
| **66** | Retrospective longitudinal cohort study | 65 children with cystic fibrosis (CwCF) at Bordeaux University Hospital | Assess telemonitoring device's ability to detect early FEV1 response following IV ATB for PEx | France | Remote monitoring through home spirometry using the Spirobank Smart connected spirometer and the Pneumotel Uploader mobile phone application. | - Primary findings: Significant drop in FEV1 8 days before IV ATB initiation; 41% of IV ATB courses failed to restore baseline FEV1.  - Effect sizes/statistical significance: FEV1 recovery less than 94% of baseline on Day 14 indicates nonresponders (sensitivity: 88%, specificity: 81%, AUC = 0.908; p < .001); greater than 110% on Day 9 indicates good responders (sensitivity: 75%, specificity: 87%, AUC = 0.863; p < .001).  - Secondary findings: Nocturnal cough increases 17 days before treatment; delay in treatment and magnitude of FEV1 drop are associated with poor response.  - Authors' main conclusions: Home spirometry facilitates early recognition of PEx; provides a threshold for identifying low responders.  - Clinical significance: Early intervention and monitoring can improve outcomes.  - Limitations: Retrospective design; influence of CFTR modulators on some patients. |
| **67** | Cross-sectional analysis of a longitudinal study | Middle-aged and older adults with underlying health conditions; sample size = 718; median age = 65.8 years; 35.0% male; 46.5% African American | Evaluate patient-reported experiences of telehealth and disparities in access, use, and satisfaction during the COVID-19 pandemic | United States (specifically, Chicago, IL, and New York, NY) | The primary telehealth format used was telephone calls, with 66.7% of participants reporting telephone visits. | - Primary findings: 47.6% of participants had a telehealth visit in the past 4 months; those with recent telehealth visits were younger, had worse health, and lived below poverty level.  - Effect sizes/statistical significance: Lower patient activation (AOR 0.19, 95% CI, 0.05-0.59) and limited English proficiency (AOR 0.12, 95% CI, 0.03-0.47) were less likely to report telehealth as better than in-person visits.  - Secondary findings: Most participants found telehealth easy to navigate and useful; disparities in access and quality persist.  - Authors' conclusions: Telehealth disparities persist; certain populations are more vulnerable to poor-quality telehealth.  - Clinical significance: Healthcare systems need to address disparities for equitable telehealth services.  - Unexpected/contradictory findings: Not mentioned. |
| **68** | Pilot study | 63 pwCF from the West of Scotland CF Unit; 40% female, 56% aged 30-49 years | To pilot an online exercise and education program for feasibility and time efficiency | Scotland, UK | The telehealth format used was primarily video calls via Zoom for live and on-demand exercise and educational sessions. Remote monitoring (like home spirometry) was mentioned in the context of education but not explicitly as part of the telehealth format used. | - Primary findings: Increases in motivation and perceived fitness; 91% of participants achieved personal fitness goals.  - Effect sizes: Trending increases in motivation (5.7 to 7.2), exertion (5.6 to 7), and fitness (4.1 to 5.9).  - Secondary findings: Positive impact on socialization and mental health; increased time efficiency for participants and clinicians.  - Authors' conclusions: Online exercise and education program is worthwhile and supports physical activity goals.  - Clinical significance: Potential for reduced hospitalization and improved quality of life; improved time efficiency.  - Unexpected findings: Significant time efficiency gained from online sessions. |
| **69** | Retrospective study | 34 patients with cystic fibrosis; Median age ± SD: 30.97±10.59; Gender distribution: 15 males, 19 females | To evaluate the role of telemonitoring during the pandemic phase of COVID-19 | Italy | Remote monitoring (home spirometry using MIR Spirotel 2) and phone calls | - Primary findings: Increase in telemedicine visits by 10% during the pandemic year; decrease in hospitalizations for pulmonary infections by 23%; increase in day-hospital visits by 22%.  - Effect sizes: 10% increase in telemedicine visits; 23% decrease in hospitalizations for pulmonary infections; 22% increase in day-hospital visits.  - Secondary findings: Median transmission rate per week was 0.80; patients categorized into groups based on transmission rates.  - Authors' main conclusions: Telemonitoring was effective in providing timely care and reducing patient exposure; telemedicine is important for managing chronic diseases during health emergencies.  - Clinical significance: Telemonitoring helped manage cystic fibrosis patients during the pandemic, reducing hospitalizations and improving remote care. |
| **70** | Qualitative | 105 caregivers responsible for children under 18 in Genesee County, MI; primarily biological parents and non-Hispanic White and African Americans | Gather caregivers' perspectives on telehealth benefits, challenges, and suggestions for improvement | United States (specifically, Genesee County, Michigan) | The telehealth format includes synchronous methods such as video and telephone visits, and asynchronous methods such as patient portal messages and remote patient monitoring (e.g., smartphone otoscopy, blood glucose monitoring). | - Primary findings: Benefits of telehealth include prevention of COVID-19 exposure, quality communication, savings in travel time, and cost-effectiveness. Challenges include lack of in-person interaction, fear of compromised confidentiality, and potential for misdiagnosis.  - Secondary findings: Caregivers prefer in-person visits for better rapport, express concern about misdiagnosis, and suggest improvements such as media campaigns and universal platforms.  - Authors' conclusions: Telehealth has benefits but faces challenges that need addressing through education and technological improvements. There is a need for future research on adolescents' opinions and socio-economic disparities.  - Clinical significance: Telehealth can reduce exposure to COVID-19 and provide cost-effective care, but improvements are needed to address challenges.  - Practical significance: Media campaigns and education can increase awareness and use of telehealth, especially among poorer families.  - Theoretical significance: The study highlights the importance of involving young patients and addressing socio-economic disparities in telehealth. |
| **71** | Feasibility study | 28 adults with cystic fibrosis and elevated clinical symptoms of anxiety and/or depression | To assess the feasibility and potential effectiveness of ACT with CF in reducing symptoms of anxiety and depression among individuals with cystic fibrosis | United States | Video call using HIPAA-compliant WebEx webcam platform | - Primary findings: Statistically significant reduction in psychological distress from pre to post treatment with a large standardized effect size; telehealth-delivered ACT with CF was as effective as in-person.  - Effect sizes/statistical significance: Large standardized effect size for reduction in psychological distress; significant reductions in anxiety and depression symptoms and cognitive fusion.  - Secondary findings: Reductions in cognitive fusion strongly related to improvements in psychosocial functioning; trend towards improved lung function (increased FEV 1 /FVC ratio).  - Authors' main conclusions: ACT with CF is feasible and potentially effective for improving anxiety and depressive symptoms and increasing psychological flexibility.  - Clinical/practical significance: ACT with CF is accessible via telehealth and may improve lung function; significant reductions in anxiety and depression symptoms.  - Unexpected findings: Trend towards improved lung function. |
| **72** | One-group pretest-posttest study | 40 participants (30 caregivers of children with CF age ≤14 years, 10 patients with CF age ≥15 years); English-speaking; treated at UAB/COA pediatric CF clinic | To test the impact of Genia on measures of patient-centered care | United States (specifically, Alabama) | Remote monitoring through online surveys and data collection via email links | - Primary findings: Increased satisfaction with care (p = 0.024), shared decision-making (p < 0.001), and nominal improvements in QOL domains and symptom scales.  - Effect sizes: CollaboRATE top score improved by 13.2 percentage points; PACIC overall score increased from 3.90 to 4.1 (p = 0.0245).  - Secondary findings: Nominal improvements in QOL domains and symptom scales; feasibility and acceptability with 95% retention rate.  - Authors' conclusions: Genia is feasible, acceptable, and improves patient-centered care outcomes; supports wider use in clinical settings.  - Clinical significance: Leverages CF care model's suitability for mHealth solutions; potential for improved patient-centered care.  - Limitations: Non-randomized design; small sample size limits generalizability. |
| **73** | Quality improvement project/descriptive study | Adult cystic fibrosis patients; sample size: 105 independent patients | Implement a hybrid telehealth model to minimize person-to-person contacts while maintaining multidisciplinary care | United States | Video call using Cisco WebEx telehealth platform | - Primary findings: Successful implementation of a hybrid telehealth model for cystic fibrosis care, reducing in-person contacts and PPE utilization by 44%.  - Effect sizes: 44% reduction in in-person contacts and PPE utilization.  - Secondary findings: High patient satisfaction with telehealth (81% felt same quality as in-person), comprehensive care with spirometry, blood work, and sputum cultures.  - Authors' main conclusions: Hybrid model is successful in maintaining patient care during the pandemic, with 88% of patients seen in quarterly follow-up.  - Clinical significance: Enables reliable access to healthcare while minimizing person-to-person contacts and PPE utilization, provides revenue for healthcare systems, and reduces economic hardship for staff. |
| **74** | Qualitative/Observational | 51 participants with cystic fibrosis, aged 2 months to 21 years, predominantly male (53%), mostly residing outside Salvador | Evaluate families' perceptions of a telehealth program during the COVID-19 pandemic | Brazil | The telehealth format included telemonitoring, individual and group telerehabilitation (likely through video calls), behavioral/emotional support, health education (through videos, webinars, and booklets), and remote appointments. | - Primary findings: High level of satisfaction with telehealth; 51% rated visual quality, personal comfort, and ease of use as excellent.  - Secondary findings: Moderate concern about lack of in-person visits; majority welcomed telehealth inclusion.  - Authors' conclusions: Telehealth addressed pandemic challenges; holds promise for improving quality of life and reducing costs.  - Clinical significance: Telehealth is effective and welcomed by patients; potential for improving outcomes in low-income countries.  - Practical significance: Telehealth can reduce costs and expand support; need for further research to standardize technologies. |
| **75** | Quality improvement initiative | Approximately 275 pediatric patients with cystic fibrosis, aged 0-21 years, from Michigan and surrounding areas | Increase successful care visits through telehealth to 95% by June 26, 2020 | United States (specifically, Michigan) | The telehealth format used was a combination of phone calls and video calls, with asynchronous virtual visits conducted on different days than physician visits. Additionally, remote monitoring was facilitated through portable home spirometry devices. | - Primary findings: Successful implementation of an interdisciplinary telehealth care model with 100% IDC visits by mid-May and maintained sustainability; high patient satisfaction with 87% extremely satisfied and 13% somewhat satisfied.  - Effect sizes: 95% success rate for IDC visits during the project; 96% success rate in the 51 weeks following the project.  - Secondary findings: Significant increase in patient portal registration from 43.9% to 90.6%; benefits of telehealth include reduced travel burden and increased patient comfort.  - Authors' main conclusions: Continuous communication and optimal technology use are crucial for successful telehealth implementation; a hybrid model combining telehealth and in-person visits is recommended.  - Clinical significance: Demonstrates the feasibility and effectiveness of telehealth in pediatric cystic fibrosis care; suggests a hybrid model for comprehensive care.  - Unexpected findings: Limitations in assessment capabilities with telehealth; need for a hybrid model to address these limitations. |
| **76** | Survey-based study | Families of patients with cystic fibrosis; sample size: 79 respondents; median age: 10 years (range 1-19 years) | Assess family satisfaction with telehealth clinics and willingness to continue beyond the pandemic | Australia, United States, United Kingdom | The telehealth format used in this study included video calls via a web-based platform called 'HealthDirect', remote monitoring through home spirometry, and self-collection of airway samples and anthropometric measurements. | - Primary findings: High satisfaction rates with telehealth clinics (46.8% satisfied, 44.3% very satisfied); 87.3% support continuation beyond COVID-19 pandemic.  - Effect sizes/statistical significance: Not explicitly mentioned.  - Secondary findings: Reduced travel time, reduced time away from work, reduced cross-infection risk; consistent support across adult and pediatric patients.  - Authors' main conclusions: Overwhelming support for telehealth; mandate for incorporation into CF care beyond pandemic.  - Clinical significance: Addresses barriers to care; improves patient satisfaction; potential for better health outcomes.  - Unexpected/contradictory findings: Not mentioned. |
| **77** | Quality improvement (QI) project | Adult cystic fibrosis patients at UVA clinic; sample size: 131 | Increase home spirometry utilization and availability in telemedicine visits | United States | Video call with remote monitoring (home spirometry) | - Primary findings: Increased percentage of adult CF patients owning home spirometers from 37% to 97% and increased spirometry results provided during telemedicine visits from 50% to 96%.  - Effect size: Mean difference in FEV1 measurements between home and in-office spirometers was 0.141 L.  - Secondary findings: Slight decline in HS utilization during December and January due to patient travel and device issues.  - Authors' main conclusions: Using QI tools to standardize home spirometry monitoring via telemedicine is reliable and sustainable.  - Clinical significance: Telemedicine can be a reliable form of CF care delivery, reducing travel and work absenteeism.  - Practical significance: Patient education and coaching are crucial for reliable spirometry results. |
| **78** | Cross-sectional survey | 2210 participants, majority female (81%), 68% older than 55 years, members of online health communities for chronic diseases | Describe telehealth use, resource needs, and information sources among individuals with chronic conditions during COVID-19 | United States | Video call (virtual appointments with doctors over video chat), phone call, online portal | - Primary findings: Nearly half (49%) of respondents engaged in telehealth services in the past four months.  - Effect sizes/statistical significance: Higher proportions of women and higher-income individuals used telehealth (P=.007 and P=.003, respectively). Aging populations were less likely to use telehealth (P<.001 and P=.001).  - Secondary findings: Patients with cystic fibrosis, lupus, and ankylosing spondylitis had higher telehealth use rates. Most participants sought information about COVID-19 and its impact on their health conditions.  - Authors' main conclusions: Telehealth is crucial for maintaining care continuity during the pandemic. Healthcare providers should engage patients, especially those from lower socioeconomic backgrounds and older populations.  - Clinical/practical significance: Telehealth supports patients with chronic conditions during the pandemic. Providers should ensure adequate information distribution about telehealth procedures.  - Unexpected/contradictory findings: Despite telehealth uptake, there is a continued need for information about COVID-19 and its effects on chronic conditions. |
| **79** | Qualitative study using semistructured interviews | 15 participants from three stakeholder groups (patients, researchers, training institutions) | Identify important attributes, facilitators, and barriers to selecting web-based platforms for PCOR teams | United States | Video call (videoconferencing), Instant messaging | - Primary findings: Key attributes for successful web-based engagement in PCOR include accessibility, ease of use, and integration with other platforms. Platforms that emulate in-person interactions, like videoconferencing and instant messaging, are crucial for regaining lost nuances and social connections.  - Secondary findings: Challenges include ensuring proper technology, handling low-bandwidth connections, and addressing digital literacy issues. Solutions include designating a technology champion and selecting integrating platforms.  - Authors' main conclusions: Successful web-based engagement requires multiple platforms to meet both asynchronous and synchronous goals. The study provides a roadmap for PCOR collaborations based on team requirements and common challenges.  - Clinical, practical, or theoretical significance: The study provides best practices for platform selection and collaboration, emphasizing the importance of accessibility, ease of use, and integration.  - Unexpected or contradictory findings: Not mentioned. |
| **80** | Observational, web-based exercise intervention | 11 participants (initially 17), aged 12-52 years, FEV1%pred. 72.3 (SD: 17.3) | Investigate feasibility and evaluate changes in exercise participation, lung function, and exercise capacity | Germany | Web-based platform with online interaction and data exchange through a website. No phone calls or video calls were mentioned. No remote monitoring tools like home spirometry were used. | - Primary findings: The web-based exercise intervention was feasible, with participants achieving a mean training duration of 178 minutes and 3.3 sessions per week. Training minutes increased by 42% from the first to the third training section.  - Effect sizes: Self-reported physical activity increased by 39.7% from baseline to 12 weeks but decreased by the end of the year. Lung function decreased slightly (FEV1 -3.9%pred.; FVC -1.9%pred.), while exercise capacity increased slightly (VO2peak + 1.5 ml/min/kg; six-minute-walk-test-distance + 26 m).  - Secondary findings: Five participants experienced significant improvements in exercise capacity despite decreases in lung function.  - Authors' main conclusions: The web-based concept is feasible and supports exercise participation. Further investigation is needed to understand the effects on lung function and exercise capacity.  - Clinical significance: Integrating this approach into usual care could promote regular personalized exercise for people with CF.  - Unexpected findings: Significant improvements in exercise capacity despite decreases in lung function for some participants. |
| **81** | 2-phase study, with the second phase evaluating feasibility, acceptability, and potential effectiveness of telehealth fertility preservation counseling. | 30 males with cystic fibrosis (MwCF) aged 22-49 years; most in a relationship (70%) and White (86.7%). | Evaluate feasibility, acceptability, and effectiveness of telehealth fertility preservation counseling for MwCF. | United States | Video call via Zoom | - Primary findings: Telehealth fertility preservation counseling is acceptable, appropriate, and feasible for males with cystic fibrosis (MwCF). - Effect sizes/statistical significance: Significant improvements in fertility preservation knowledge (p = .010), care satisfaction (p < .001), and self-efficacy (p = .016). - Secondary findings: 44% of participants did not receive information about infertility from their CF team despite wanting provider-initiated SRH education. - Authors' main conclusions: Integrating fertility preservation counseling into CF care is feasible and acceptable and improves knowledge and satisfaction. - Clinical/practical significance: Improves reproductive health outcomes and informed decision-making; telehealth is efficient and convenient, improving access. - Theoretical significance: Standardization of SRH and fertility preservation education is critical for comprehensive CF care. |
| **82** | Prospective, single-center, assessor-blinded, parallel-group randomized controlled study | 39 children with cystic fibrosis, mean age=11.41±2.18 years, mean FEV1 (z-score)=-0.66±1.96 | Evaluate the effect of telehealth-based exercise training on exercise capacity, oxidative stress, and irisin levels in children with CF | Turkey | Video conferencing | - Primary findings: The combined exercise training group showed significant improvement in exercise capacity (MST distance and percentage) compared to the core stabilization group and control group. - Effect sizes: np 2 =0.157 for MST distance improvement. - Secondary findings: No significant changes in oxidative stress parameters or irisin levels between groups. - Authors' main conclusions: Telehealth-based combined exercise training improves exercise capacity in children with CF with mild-to-moderate lung disease. - Clinical significance: Regular exercise is recommended in CF management; telehealth-based exercise training is beneficial. - Limitations: Excluded children with advanced lung disease; no group focused solely on aerobic exercise training. |
| **83** | Multisite randomized waitlist-controlled trial | Adults with cystic fibrosis (AWCF) reporting mild to severe symptoms of depression and/or anxiety; sample size = 132 (66 in each group) | Test the efficacy of CALM in reducing depression and anxiety symptoms in AWCF | United States | Video call (using Zoom) | - Primary findings: CALM significantly reduced depression and anxiety symptoms in adults with cystic fibrosis compared to a waitlist control group. - Effect sizes: Large effect size for depression post-intervention (d = 0.85), medium effect size at 1-month follow-up (d = 0.70); medium effect sizes for anxiety post-intervention (d = 0.65) and at 1-month follow-up (d = 0.66). - Secondary findings: Significant improvements in coping self-efficacy, stress, and vitality. - Authors' conclusions: CALM is an effective intervention with sustained treatment gains over a 3-month follow-up period. - Clinical significance: First psychosocial intervention to show efficacy in reducing depression and anxiety in adults with cystic fibrosis. - Practical significance: Recommended for dissemination and implementation in CF centers to provide high-quality mental health services. |
| **84** | Qualitative | 67 participants from various roles (genetic counselors, CF center staff, people with CF, partners, payors) | Assess acceptability of centralized CF genetic counseling telehealth service | United States and Puerto Rico | Video call | - Primary findings: A centralized CFgc-T service can improve equitable access to CF-specific genetic counseling; 58% of clinicians and 96% of consumers are interested in the service. - Effect sizes/statistical significance: Not explicitly mentioned. - Secondary findings: Telehealth delivery provides equivalent outcomes to in-person counseling at a lower cost; CFgc-T addresses licensure, language, and testing access issues. - Authors' main conclusions: CFgc-T can improve access to high-quality genetic counseling, reduce disparities, and serve as a paradigm for disease-specific counseling. - Clinical/practical significance: Addresses workforce shortages and geographic barriers; provides equivalent outcomes at lower cost. - Unexpected/contradictory findings: Not mentioned. |
| **85** | Randomized controlled trial (RCT) | Adolescents with cystic fibrosis, aged 12-18 years; sample size: 21 | Evaluate the effect of a 12-week VR-based tele-exercise on 6-min walk distance (6MWD) | Turkey | The telehealth format used in the study was a combination of video calls (using Microsoft Teams) and remote monitoring (using VR headsets). | - Primary finding: 12-week VR-based tele-exercise significantly improved 6-minute walk distance (6MWD) in adolescents with cystic fibrosis (z = -2.93, p = 0.003). - Secondary findings: No significant improvements in pulmonary function, muscle strength, or quality of life. - Effect sizes/statistical significance: Improvement in 6MWD was statistically significant and clinically meaningful. - Authors' conclusions: VR and tele-exercise are feasible and motivating methods to improve functional capacity; longer durations may be needed for other benefits. - Clinical significance: Overcomes barriers to physical activity; improves functional capacity. - Unexpected findings: No superiority of VR over traditional tele-exercise in improving 6MWD. |
| **86** | Cross-sectional survey study | 185 individuals (90% caregivers, 10% adults 18-25 years); demographics include age and self-reported race/ethnicity | Understand patient and caregiver experience with telehealth in the pediatric pulmonary clinic post-pandemic | United States | Not mentioned (the paper does not specify the type of telehealth format used) | - Primary findings: 71% found telehealth more convenient, 95% considered it an acceptable alternative. - Effect sizes: 75% not concerned about lack of in-person assessments, 21% somewhat concerned, 4% very concerned. - Secondary findings: 94% would use telehealth again, reduced travel time and less missed work and school. - Authors' conclusions: Telehealth is convenient and acceptable, desired by patients and families. - Clinical significance: Telehealth is a valuable option for continued access, but further research is needed on its impact on clinical outcomes. |
| **87** | Prospective observational study | 59 children aged 5-17 years from all four pediatric CF centers in Sweden |  | Sweden | Video calls for telemedicine visits and remote monitoring through home spirometry. | - Primary findings: No significant differences in lung function progression (FEV1%, lung clearance index, BMI) between intervention and prepandemic periods. - Effect sizes/statistical significance: Mean differences with confidence intervals and p-values for FEV1%, lung clearance index, and BMI. - Secondary findings: No major shifts in airway pathogens, sputum cultures, or antibiotics use; no increase in stress; high satisfaction with home spirometry and telemedicine. - Authors' main conclusions: Telehealth and home spirometry are as effective as in-person care with enhanced flexibility and personalization. - Clinical significance: Safe introduction of telehealth and home spirometry in pediatric CF care. - Unexpected findings: Significant reduction in Haemophilus influenzae incidence; high correlation between hospital and home spirometry results despite higher variability in home spirometry. |
| **88** | Cross-sectional, survey study | 56 clinicians from seven US CF centers (48% pediatric, 38% adult, 14% med/peds) |  | United States | The telehealth format includes video calls (single discipline and interdisciplinary with asynchronous care), screenshare functions for education and imaging review, and remote monitoring tools such as home spirometry, home scale weights, and home oximetry, with integration of lab and sputum culture data. | - Primary findings: Clinician perceptions of telehealth remain high but have changed over time, with a decrease in satisfaction from 90% in 2020 to 75% in the current study (p = 0.02). - Effect sizes/statistical significance: Decrease in satisfaction (p = 0.02), significant decrease in concern over missing in-person assessments. - Secondary findings: Technology limitations and limited in-person assessments are major barriers; benefits include convenience and reduced missed days of school or work. - Authors' main conclusions: TH is an acceptable and desirable method for delivering care to pwCF, with numerous benefits; it should remain part of routine CF care. - Clinical significance: TH is integrated into routine care despite restored access to in-person care; it offers convenience and reduces missed days of school or work. - Unexpected findings: Decrease in concerns over missing objective assessments despite persistent concerns. |
| **89** | Multicenter randomized trial | 66 adult patients with cystic fibrosis (pwCF), with 43 having minimal symptoms of depression and 44 having minimal symptoms of anxiety at baseline | To investigate the impact of the UPLIFT intervention on symptoms of anxiety and depression in pwCF | United States | Video call (using Zoom) | - Primary findings: UPLIFT significantly reduced depressive symptoms (PHQ-9 scores) compared to TAU (p = .049), with significant difference at treatment end (p = .005). - Effect sizes/statistical significance: PHQ-9 scores decreased by -2.321 (SD 0.684) in UPLIFT vs. 0.362 (SD 0.656) in TAU. - Secondary findings: Trends suggested improvement in anxiety symptoms, but not statistically significant. - Authors' main conclusions: UPLIFT is a feasible, convenient, and cost-effective approach for reducing depressive symptoms in adults with CF. - Clinical significance: Improves mindfulness skills and peer connections, reducing social isolation. - Unexpected findings: Immediate effects were not maintained; sustainability may require additional sessions or booster sessions. |
| **90** | Single-site, prospective cohort study | 38 adults with chronic respiratory disease; mean age 68 years; 47% female | Assess safety, reliability, and acceptability of MIST supervised remotely via videoconferencing | Australia | Videoconferencing (using Zoom), remote monitoring (using pulse oximeter and other equipment), and phone calls for support/troubleshooting. | - Primary findings: Excellent agreement between in-person and remote testing for total step count (ICC 2,1 0.93, 95%CI 0.86 to 0.96), with higher counts in in-person testing (MD 12 steps, 95%CI 1 to 24). Very good agreement for nadir oxygen saturation (ICC 2,1 0.797, 95%CI 0.643 to 0.889) and peak heart rate (ICC 2,1 0.782, 95%CI 0.620 to 0.880). - Secondary findings: High participant satisfaction with telehealth, no difference in confidence between testing conditions, no adverse events. - Authors' main conclusions: MIST can be safely and reliably administered with remote supervision, which is acceptable to patients. Remote assessment may broaden rehabilitation options and support equitable service access. - Clinical significance: Potential for remote assessment to improve access to pulmonary rehabilitation, with implications for patient outcomes and healthcare utilization. - Unexpected findings: Difference in performance favoring in-person supervision, but within minimal important difference. |
| **91** | Cross-sectional study | 23 patients with cystic fibrosis (CF), aged 6-18 years, mean age 10.7 years, mean FEV1 89.5% | Assess feasibility and reproducibility of 3-minute step test with remote supervision in children and adolescents with CF | Brazil | Video call combined with remote monitoring (using an oximeter) | - Primary findings: No significant differences in physiological responses between in-person and remote supervision of the 3-minute step test. - Effect sizes: Mean differences and confidence intervals for heart rate and oxygen saturation showed no significant differences. - Secondary findings: High ICC values for final heart rate, oxygen saturation, and lower limb fatigue; small mean difference in final SpO2 in Bland-Altman analysis. - Authors' main conclusions: Remote supervision is feasible and can help monitor CF patients more effectively. - Clinical significance: Remote supervision can overcome barriers to care, especially during the COVID-19 pandemic. - Limitations: Small sample size, mild lung function impairment in the cohort. |
| **92** | Randomized controlled trial (RCT) | 124 adults with cystic fibrosis (CF) and elevated anxiety and/or depressive symptoms; mean age 25 years, 75% female, 67% on CFTR modulators | To determine if ACT with CF is superior to SP in improving psychological functioning among adults with CF | United States | Video call via HIPAA-compliant Zoom | - Primary findings: ACT with CF was superior to SP in improving psychological functioning among adults with CF and elevated psychological distress. - Effect sizes: Cohen's d = 0.59 for improvements in psychological functioning at 6 weeks; Cohen's d = 0.40 for sustained improvements at 3 months. - Secondary findings: Improvements in psychological flexibility associated with reductions in negative affect; ACT reduced self-reported barriers to treatment adherence. - Authors' conclusions: ACT with CF is effective for improving psychological flexibility and reducing barriers to treatment adherence. - Clinical significance: Sustained improvements in psychological functioning; potential for improving treatment adherence. - Theoretical significance: Psychological flexibility as a transdiagnostic target for improving psychological functioning in individuals with CF. |
| **93** | Qualitative | 10 adults with CF, 16 parents of children with CF; varied socioeconomic levels and geographical locations | Explore acceptability of hybrid model of care | Australia | The telehealth format used was primarily audiovisual appointments, with some participants also using telephone-only appointments occasionally. | - Primary findings: The hybrid model of care is acceptable to PWCF and parents, with benefits including reduced treatment burden and cross-infection risk. - Secondary findings: Concerns about thoroughness of assessment and need for FTF visits; variability in acceptability based on individual willingness to compromise. - Authors' conclusions: The hybrid model is acceptable but should be personalized to individual needs; reduced treatment burden and cross-infection risk are key benefits. - Clinical significance: The study supports the use of a hybrid model in CF care, emphasizing flexibility and personalization. - Limitations: Focus on PWCF and parents' perspectives; potential lack of generalizability to other populations or healthcare systems. |
| **94** | Retrospective, multicenter, cross-sectional study | All persons with CF (PwCF) in the CF Foundation Registry from 1/2020-12/2021; sample size: 4762 PwCF in 2020, 4806 PwCF in 2021 | Evaluate the impact of standardized telehealth implementation on access to care and clinical outcomes | United States | The telehealth format included remote monitoring, such as home spirometry, but the paper does not specify whether phone calls or video calls were used. | - Primary findings: Higher average number of telehealth visits and percentages of patients with at least one telehealth visit in the CFLN TH-iLab compared to other groups. - Effect sizes/statistical significance: Not explicitly mentioned in the quotes. - Secondary findings: Lung function was highest in the CFLN TH-iLab; anthropometric measurements and microbiology cultures were similar across groups; access to interdisciplinary care was highest in the CFLN non-TH-iLab. - Authors' main conclusions: Telehealth provided access to care without compromising clinical outcomes; further research is needed to optimize the telehealth experience. - Clinical significance: Telehealth can be effectively integrated into cystic fibrosis care without compromising outcomes. - Unexpected findings: Higher access to interdisciplinary care in the CFLN non-TH-iLab group. |
| **95** | Retrospective longitudinal study | 35 adolescents with CF (≥ 12 years of age), mean age 11.0 ± 2.7 years, 54.3% female | To verify the impact of telehealth during the COVID-19 pandemic on clinical and nutritional characteristics of adolescents with CF | Brazil | The telehealth format included teleconsultations conducted via video calls or phone calls, depending on patient availability, and telemonitoring for patients with more severe disease. Emergency consultations were also available. | - Primary findings: Reduction in H/A and BMI/A, increase in malnutrition according to pAMC, decline in FEV1% and FVC%, stable FEV1/FVC ratio. - Statistical significance: Observed between 2018-2019 and 2021-2022 for lung function parameters. - Secondary findings: Telehealth maintained clinical and nutritional parameters. - Authors' conclusions: Telehealth is an important tool in managing CF, maintaining parameters during the pandemic. - Clinical significance: Telehealth is effective in maintaining patient care during social isolation. |
| **96** | Feasibility study | 32 patients with cystic fibrosis (12 males, 20 females), average age 20.52 years | Evaluate the feasibility of a home web-based exercise training program for patients with cystic fibrosis during the COVID-19 pandemic | Italy | Video calls through Zoom platform for supervised group training sessions; remote monitoring of physical activity through home exercises; remote spirometry, bioimpedance analysis, and handgrip tests. | The study found that a home web-based exercise program for cystic fibrosis patients during COVID-19 was feasible, with moderate adherence (61.5%) and high satisfaction (Likert scores: 4.5 for satisfaction, 4.3 for utility). A significant improvement was noted in the "Body" domain of CFQ-R (P<0.05). The authors concluded that web-based rehabilitation could effectively increase physical activity in CF patients, though further studies are needed to confirm adherence levels and long-term physiological effects. No significant correlation was found between adherence and broader quality of life improvements. The study underscores the potential of tele-rehabilitation during pandemics. |
| **97** | Retrospective cohort study | 1250 patients (646 < 18 yrs, 594 ≥ 18 yrs) | Examine relationship between telehealth usage and health outcomes in CF | Australia | The telehealth format included a combination of video calls (audiovisual) and phone calls (audio-only), with remote monitoring through home spirometry. | - Primary findings: Reduced decline in ppFEV1 in adults (β = 6.06, p < 0.001) and lower detection of Pseudomonas aeruginosa in adults (OR = 0.419, p = 0.035) and children (OR = 0.182, p = 0.011) with >75% telehealth visits. - No association with BMI or hospitalizations. - Higher telehealth usage not associated with adverse outcomes; beneficial for lung function in adults. - Reduced microbiological sampling with increased telehealth use. - Telehealth is safe and useful as an adjunct to in-person care, pending resolution of sampling challenges. |
| **98** | Qualitative | 12 MDT members, 10 PwCF; MDT members: physiotherapists, occupational therapists, psychologists, dieticians, social workers, nurses, physiotherapy technicians, exercise therapists; PwCF: adults diagnosed with CF with experience using RES | Explore experiences and perceptions of RES and inform future recommendations | UK, Australia, US, and Belgium. | Videoconferencing | - Primary findings: Positive perceptions of RES by MDT members and PwCF, with benefits including time and cost savings, improved work efficiency, and peer support. - Barriers: Technological issues and lack of visual cues. - Recommendations: Personalization of exercise options, user-friendly platforms, funding for equipment. - Clinical significance: Integration of RES into routine cystic fibrosis care for those with geographical or social constraints. - Practical significance: Improved work efficiency and reduced costs during COVID-19. - Theoretical significance: Application of BCW to understand and improve RES. |

**Table S4.** Scores for all 98 papers [1-98] for the iCHECK checklist.

| Study ID | Title Score | Abstract Score | Context Score | Problem Statement Score | similar interventions Score | Aim and Objectives Score | Blueprint Summary Score | Technical Design Score | Target Score | Data Score | Interoperability Score | Participating entities Score | Budget Planning Score | Sustainability Score | Coverage Score | Outcomes Score | Lessons Learned Score | Unintended Consequences Score | Conclusion Score | General Score |
| --- | --- | --- | --- | --- | --- | --- | --- | --- | --- | --- | --- | --- | --- | --- | --- | --- | --- | --- | --- | --- |
| 1 | 2 | 2 | 1 | 2 | 2 | 1 | 2 | 1 | 2 | 1 | 0 | 1 | 0 | 1 | 1 | 2 | 1 | 1 | 2 | 1 |
| 2 | 1 | 2 | 1 | 2 | 1 | 2 | 2 | 0 | 2 | 1 | 0 | 1 | 0 | 0 | 1 | 2 | 1 | 0 | 2 | 1 |
| 3 | 1 | 2 | 1 | 2 | 1 | 1 | 1 | 1 | 2 | 1 | 0 | 1 | 0 | 1 | 1 | 2 | 1 | 1 | 2 | 1 |
| 4 | 2 | 2 | 1 | 1 | 1 | 1 | 1 | 1 | 2 | 0 | 1 | 1 | 0 | 1 | 1 | 0 | 1 | 0 | 1 | 0 |
| 5 | 2 | 2 | 1 | 2 | 1 | 2 | 1 | 1 | 2 | 1 | 0 | 0 | 0 | 1 | 1 | 2 | 1 | 0 | 2 | 1 |
| 6 | 1 | 2 | 1 | 1 | 1 | 2 | 1 | 1 | 2 | 0 | 1 | 1 | 1 | 0 | 1 | 2 | 1 | 1 | 2 | 1 |
| 7 | 1 | 2 | 1 | 2 | 1 | 1 | 1 | 0 | 2 | 0 | 0 | 0 | 0 | 0 | 0 | 2 | 1 | 2 | 2 | 1 |
| 8 | 1 | 2 | 1 | 1 | 1 | 0 | 0 | 0 | 1 | 1 | 0 | 1 | 0 | 1 | 1 | 2 | 1 | 1 | 2 | 2 |
| 9 | 2 | 2 | 1 | 2 | 1 | 0 | 0 | 1 | 2 | 0 | 0 | 1 | 0 | 1 | 1 | 1 | 1 | 1 | 2 | 0 |
| 10 | 1 | 2 | 1 | 1 | 1 | 1 | 1 | 1 | 2 | 0 | 0 | 1 | 0 | 1 | 2 | 1 | 1 | 1 | 2 | 1 |
| 11 | 2 | 2 | 1 | 1 | 1 | 1 | 1 | 0 | 2 | 1 | 0 | 1 | 0 | 0 | 1 | 2 | 1 | 0 | 2 | 1 |
| 12 | 0 | 2 | 1 | 2 | 1 | 1 | 1 | 1 | 2 | 1 | 0 | 1 | 0 | 1 | 1 | 2 | 1 | 1 | 2 | 2 |
| 13 | 0 | 2 | 1 | 2 | 1 | 1 | 0 | 0 | 2 | 0 | 0 | 0 | 0 | 0 | 2 | 2 | 1 | 1 | 2 | 1 |
| 14 | 1 | 2 | 1 | 2 | 1 | 1 | 2 | 1 | 2 | 1 | 0 | 1 | 0 | 0 | 2 | 0 | 1 | 1 | 2 | 1 |
| 15 | 0 | 2 | 1 | 2 | 1 | 1 | 1 | 1 | 2 | 1 | 1 | 1 | 0 | 1 | 1 | 2 | 1 | 2 | 2 | 1 |
| 16 | 2 | 2 | 1 | 2 | 1 | 2 | 2 | 1 | 2 | 1 | 0 | 0 | 0 | 0 | 0 | 2 | 1 | 1 | 2 | 2 |
| 17 | 2 | 2 | 1 | 2 | 1 | 2 | 2 | 0 | 2 | 0 | 0 | 1 | 0 | 1 | 1 | 2 | 1 | 1 | 1 | 2 |
| 18 | 1 | 2 | 1 | 2 | 1 | 1 | 1 | 1 | 2 | 1 | 0 | 1 | 0 | 1 | 1 | 2 | 1 | 1 | 2 | 1 |
| 19 | 2 | 2 | 1 | 1 | 1 | 1 | 2 | 1 | 2 | 1 | 0 | 1 | 0 | 1 | 1 | 1 | 1 | 1 | 2 | 1 |
| 20 | 2 | 2 | 1 | 2 | 2 | 2 | 2 | 1 | 2 | 1 | 0 | 1 | 1 | 1 | 1 | 2 | 1 | 1 | 2 | 1 |
| 21 | 2 | 2 | 1 | 2 | 1 | 2 | 2 | 0 | 2 | 1 | 0 | 0 | 0 | 0 | 1 | 2 | 1 | 1 | 2 | 1 |
| 22 | 2 | 2 | 1 | 2 | 1 | 1 | 1 | 0 | 2 | 0 | 0 | 1 | 0 | 0 | 1 | 2 | 1 | 2 | 2 | 1 |
| 23 | 2 | 1 | 1 | 2 | 1 | 1 | 1 | 1 | 2 | 1 | 0 | 1 | 0 | 1 | 1 | 2 | 1 | 1 | 2 | 1 |
| 24 | 2 | 2 | 1 | 2 | 1 | 2 | 1 | 1 | 2 | 1 | 0 | 1 | 0 | 1 | 1 | 2 | 1 | 2 | 2 | 2 |
| 25 | 2 | 1 | 1 | 2 | 1 | 1 | 2 | 1 | 2 | 1 | 1 | 1 | 0 | 1 | 1 | 1 | 1 | 2 | 2 | 0 |
| 26 | 1 | 2 | 1 | 2 | 2 | 2 | 2 | 1 | 2 | 1 | 0 | 1 | 0 | 1 | 1 | 2 | 1 | 0 | 2 | 1 |
| 27 | 1 | 2 | 1 | 2 | 1 | 2 | 1 | 1 | 2 | 0 | 1 | 1 | 0 | 1 | 1 | 2 | 1 | 1 | 2 | 2 |
| 28 | 0 | 2 | 1 | 1 | 0 | 0 | 0 | 0 | 2 | 1 | 0 | 0 | 0 | 1 | 1 | 1 | 1 | 2 | 2 | 1 |
| 29 | 0 | 1 | 1 | 2 | 1 | 1 | 1 | 1 | 2 | 0 | 1 | 0 | 0 | 1 | 1 | 1 | 1 | 1 | 2 | 1 |
| 30 | 2 | 2 | 1 | 2 | 1 | 2 | 1 | 1 | 2 | 1 | 0 | 1 | 0 | 0 | 1 | 2 | 1 | 1 | 2 | 2 |
| 31 | 1 | 2 | 1 | 2 | 1 | 2 | 0 | 0 | 2 | 1 | 0 | 0 | 0 | 0 | 2 | 2 | 1 | 1 | 2 | 1 |
| 32 | 1 | 2 | 1 | 2 | 1 | 2 | 1 | 1 | 2 | 1 | 0 | 0 | 0 | 1 | 1 | 2 | 1 | 1 | 2 | 1 |
| 33 | 2 | 2 | 1 | 2 | 1 | 1 | 1 | 1 | 2 | 1 | 0 | 0 | 0 | 0 | 1 | 2 | 1 | 2 | 2 | 1 |
| 34 | 2 | 2 | 1 | 2 | 2 | 2 | 1 | 1 | 2 | 1 | 0 | 1 | 1 | 1 | 1 | 2 | 1 | 1 | 2 | 1 |
| 35 | 2 | 2 | 1 | 2 | 1 | 2 | 1 | 1 | 2 | 0 | 0 | 0 | 1 | 1 | 0 | 2 | 1 | 1 | 2 | 1 |
| 36 | 2 | 2 | 1 | 2 | 1 | 1 | 2 | 1 | 2 | 1 | 0 | 1 | 0 | 0 | 1 | 1 | 1 | 2 | 2 | 2 |
| 37 | 0 | 2 | 1 | 1 | 1 | 0 | 1 | 0 | 2 | 1 | 0 | 0 | 0 | 0 | 0 | 2 | 1 | 1 | 2 | 1 |
| 38 | 1 | 2 | 1 | 2 | 1 | 1 | 1 | 1 | 2 | 1 | 0 | 1 | 0 | 0 | 1 | 2 | 1 | 1 | 2 | 1 |
| 39 | 2 | 2 | 1 | 2 | 1 | 2 | 2 | 0 | 2 | 1 | 0 | 1 | 1 | 1 | 1 | 2 | 1 | 1 | 2 | 2 |
| 40 | 2 | 2 | 1 | 2 | 1 | 1 | 2 | 0 | 2 | 0 | 0 | 1 | 0 | 1 | 0 | 2 | 1 | 1 | 2 | 0 |
| 41 | 2 | 2 | 1 | 2 | 0 | 1 | 1 | 1 | 2 | 1 | 0 | 1 | 0 | 0 | 1 | 2 | 1 | 1 | 1 | 1 |
| 42 | 2 | 2 | 1 | 1 | 1 | 1 | 1 | 1 | 1 | 0 | 0 | 1 | 0 | 0 | 1 | 2 | 1 | 1 | 1 | 0 |
| 43 | 2 | 2 | 1 | 2 | 1 | 1 | 2 | 1 | 2 | 1 | 0 | 1 | 0 | 1 | 1 | 1 | 1 | 1 | 1 | 1 |
| 44 | 1 | 2 | 1 | 2 | 1 | 1 | 1 | 1 | 2 | 1 | 0 | 1 | 0 | 1 | 1 | 1 | 1 | 1 | 2 | 2 |
| 45 | 1 | 2 | 1 | 2 | 1 | 2 | 2 | 1 | 2 | 1 | 0 | 1 | 0 | 0 | 0 | 2 | 1 | 1 | 2 | 1 |
| 46 | 2 | 2 | 1 | 2 | 1 | 2 | 2 | 1 | 2 | 1 | 0 | 1 | 0 | 0 | 2 | 2 | 1 | 1 | 2 | 1 |
| 47 | 1 | 2 | 1 | 2 | 1 | 1 | 2 | 1 | 2 | 1 | 0 | 1 | 0 | 0 | 0 | 2 | 1 | 1 | 2 | 1 |
| 48 | 2 | 2 | 1 | 2 | 2 | 2 | 2 | 1 | 2 | 1 | 0 | 1 | 0 | 0 | 0 | 2 | 1 | 1 | 2 | 1 |
| 49 | 2 | 2 | 1 | 2 | 1 | 1 | 1 | 1 | 2 | 1 | 0 | 1 | 0 | 0 | 1 | 2 | 1 | 1 | 2 | 2 |
| 50 | 2 | 2 | 1 | 2 | 1 | 1 | 1 | 0 | 2 | 1 | 0 | 1 | 0 | 0 | 1 | 0 | 1 | 1 | 2 | 1 |
| 51 | 0 | 2 | 1 | 2 | 0 | 2 | 2 | 0 | 1 | 0 | 0 | 1 | 0 | 1 | 1 | 2 | 1 | 0 | 2 | 0 |
| 52 | 2 | 2 | 1 | 2 | 1 | 1 | 2 | 0 | 2 | 1 | 0 | 1 | 0 | 1 | 1 | 2 | 1 | 1 | 2 | 1 |
| 53 | 1 | 1 | 1 | 1 | 1 | 2 | 1 | 1 | 1 | 0 | 0 | 1 | 0 | 1 | 1 | 2 | 1 | 2 | 2 | 0 |
| 54 | 1 | 1 | 1 | 2 | 1 | 2 | 2 | 1 | 2 | 0 | 0 | 0 | 0 | 0 | 1 | 2 | 1 | 1 | 2 | 0 |
| 55 | 1 | 2 | 1 | 2 | 1 | 1 | 0 | 0 | 1 | 0 | 0 | 0 | 0 | 1 | 1 | 2 | 1 | 1 | 2 | 0 |
| 56 | 2 | 2 | 1 | 2 | 1 | 1 | 2 | 1 | 1 | 1 | 0 | 1 | 0 | 1 | 1 | 2 | 1 | 1 | 2 | 1 |
| 57 | 2 | 2 | 1 | 2 | 1 | 1 | 2 | 1 | 2 | 1 | 0 | 1 | 1 | 1 | 1 | 1 | 1 | 1 | 2 | 1 |
| 58 | 1 | 2 | 1 | 2 | 1 | 1 | 2 | 1 | 2 | 1 | 1 | 1 | 0 | 1 | 1 | 0 | 1 | 1 | 2 | 1 |
| 59 | 1 | 2 | 1 | 1 | 1 | 1 | 1 | 0 | 2 | 1 | 0 | 0 | 0 | 0 | 1 | 1 | 1 | 1 | 2 | 1 |
| 60 | 1 | 2 | 1 | 2 | 0 | 1 | 0 | 0 | 1 | 0 | 0 | 0 | 0 | 0 | 1 | 2 | 1 | 1 | 2 | 1 |
| 61 | 2 | 2 | 1 | 2 | 1 | 2 | 1 | 1 | 2 | 1 | 0 | 1 | 0 | 1 | 1 | 2 | 1 | 2 | 2 | 1 |
| 62 | 2 | 2 | 1 | 2 | 1 | 1 | 2 | 1 | 2 | 1 | 0 | 0 | 0 | 0 | 1 | 2 | 1 | 2 | 2 | 1 |
| 63 | 1 | 2 | 1 | 1 | 1 | 1 | 2 | 1 | 2 | 1 | 0 | 1 | 0 | 1 | 1 | 2 | 1 | 1 | 1 | 1 |
| 64 | 1 | 2 | 1 | 2 | 1 | 2 | 1 | 1 | 2 | 1 | 0 | 0 | 0 | 0 | 1 | 2 | 1 | 1 | 2 | 1 |
| 65 | 2 | 2 | 1 | 2 | 1 | 2 | 2 | 1 | 2 | 1 | 1 | 1 | 0 | 0 | 1 | 2 | 1 | 1 | 1 | 1 |
| 66 | 1 | 2 | 1 | 2 | 1 | 1 | 1 | 1 | 2 | 1 | 0 | 0 | 0 | 1 | 1 | 2 | 1 | 1 | 2 | 1 |
| 67 | 2 | 2 | 1 | 2 | 1 | 2 | 1 | 1 | 2 | 1 | 0 | 1 | 0 | 1 | 1 | 2 | 1 | 1 | 2 | 2 |
| 68 | 1 | 2 | 1 | 2 | 1 | 1 | 1 | 1 | 2 | 1 | 0 | 1 | 0 | 0 | 1 | 2 | 1 | 1 | 2 | 1 |
| 69 | 1 | 2 | 1 | 2 | 1 | 1 | 0 | 0 | 2 | 1 | 0 | 1 | 0 | 0 | 1 | 2 | 1 | 1 | 2 | 1 |
| 70 | 2 | 2 | 1 | 2 | 1 | 2 | 2 | 1 | 2 | 1 | 0 | 1 | 0 | 1 | 1 | 2 | 1 | 1 | 2 | 1 |
| 71 | 1 | 2 | 1 | 2 | 1 | 2 | 1 | 1 | 1 | 0 | 0 | 1 | 0 | 0 | 1 | 2 | 1 | 1 | 2 | 0 |
| 72 | 0 | 2 | 1 | 1 | 0 | 0 | 0 | 0 | 1 | 1 | 0 | 0 | 0 | 1 | 1 | 1 | 1 | 2 | 2 | 1 |
| 73 | 2 | 2 | 1 | 2 | 1 | 2 | 2 | 1 | 2 | 1 | 1 | 1 | 0 | 0 | 1 | 2 | 1 | 0 | 2 | 2 |
| 74 | 2 | 0 | 1 | 2 | 1 | 1 | 1 | 1 | 2 | 1 | 0 | 1 | 0 | 1 | 1 | 2 | 1 | 1 | 2 | 1 |
| 75 | 2 | 2 | 1 | 2 | 1 | 2 | 2 | 1 | 2 | 1 | 1 | 1 | 0 | 1 | 1 | 2 | 1 | 1 | 2 | 1 |
| 76 | 1 | 1 | 1 | 2 | 1 | 1 | 2 | 1 | 2 | 0 | 0 | 1 | 0 | 0 | 1 | 2 | 1 | 1 | 2 | 1 |
| 77 | 2 | 2 | 1 | 2 | 1 | 2 | 2 | 1 | 2 | 0 | 1 | 1 | 1 | 1 | 1 | 2 | 1 | 2 | 2 | 2 |
| 78 | 1 | 2 | 1 | 2 | 0 | 1 | 0 | 0 | 2 | 0 | 0 | 1 | 0 | 0 | 1 | 2 | 1 | 1 | 2 | 0 |
| 79 | 2 | 2 | 1 | 2 | 2 | 2 | 1 | 1 | 2 | 1 | 0 | 1 | 0 | 1 | 1 | 2 | 1 | 1 | 2 | 2 |
| 80 | 2 | 2 | 1 | 1 | 1 | 2 | 2 | 1 | 2 | 1 | 0 | 1 | 0 | 0 | 1 | 2 | 1 | 1 | 2 | 1 |
| 81 | 2 | 2 | 1 | 2 | 0 | 2 | 2 | 0 | 2 | 0 | 0 | 1 | 1 | 0 | 0 | 2 | 1 | 0 | 0 | 2 |
| 82 | 2 | 2 | 1 | 2 | 2 | 2 | 2 | 0 | 2 | 0 | 0 | 2 | 0 | 0 | 2 | 0 | 1 | 0 | 2 | 2 |
| 83 | 2 | 2 | 2 | 2 | 2 | 2 | 2 | 2 | 2 | 0 | 0 | 1 | 0 | 0 | 0 | 2 | 1 | 0 | 0 | 2 |
| 84 | 2 | 1 | 1 | 2 | 1 | 0 | 2 | 2 | 2 | 0 | 0 | 1 | 1 | 0 | 0 | 0 | 0 | 2 | 0 | 2 |
| 85 | 2 | 1 | 1 | 2 | 1 | 2 | 2 | 0 | 2 | 0 | 0 | 1 | 0 | 0 | 0 | 0 | 1 | 2 | 2 | 0 |
| 86 | 1 | 2 | 2 | 2 | 0 | 0 | 0 | 0 | 2 | 0 | 0 | 1 | 0 | 0 | 0 | 0 | 1 | 2 | 0 | 2 |
| 87 | 2 | 2 | 1 | 2 | 1 | 2 | 2 | 0 | 2 | 0 | 1 | 0 | 0 | 0 | 0 | 2 | 1 | 0 | 0 | 2 |
| 88 | 2 | 1 | 1 | 1 | 0 | 0 | 0 | 0 | 2 | 0 | 0 | 1 | 0 | 0 | 0 | 0 | 1 | 0 | 0 | 2 |
| 89 | 2 | 2 | 2 | 2 | 1 | 2 | 2 | 0 | 2 | 0 | 1 | 1 | 1 | 0 | 2 | 2 | 1 | 0 | 0 | 2 |
| 90 | 2 | 2 | 1 | 2 | 2 | 2 | 0 | 0 | 2 | 0 | 0 | 0 | 1 | 0 | 0 | 2 | 2 | 0 | 2 | 2 |
| 91 | 2 | 2 | 1 | 2 | 2 | 2 | 2 | 0 | 2 | 0 | 0 | 1 | 0 | 0 | 0 | 2 | 1 | 0 | 0 | 2 |
| 92 | 2 | 2 | 2 | 2 | 1 | 2 | 2 | 0 | 2 | 0 | 1 | 1 | 0 | 0 | 0 | 2 | 0 | 0 | 0 | 2 |
| 93 | 0 | 2 | 2 | 1 | 0 | 0 | 0 | 0 | 2 | 0 | 1 | 1 | 0 | 0 | 2 | 0 | 0 | 2 | 2 | 2 |
| 94 | 2 | 2 | 1 | 2 | 0 | 2 | 0 | 0 | 2 | 0 | 0 | 1 | 0 | 0 | 2 | 2 | 1 | 0 | 2 | 2 |
| 95 | 2 | 0 | 1 | 2 | 2 | 2 | 2 | 0 | 2 | 0 | 0 | 1 | 0 | 0 | 0 | 2 | 1 | 2 | 0 | 2 |
| 96 | 2 | 1 | 1 | 2 | 1 | 2 | 2 | 0 | 2 | 0 | 0 | 0 | 0 | 0 | 0 | 2 | 1 | 0 | 0 | 2 |
| 97 | 2 | 2 | 1 | 2 | 1 | 2 | 0 | 0 | 2 | 0 | 0 | 1 | 0 | 0 | 2 | 2 | 1 | 2 | 0 | 2 |
| 98 | 2 | 0 | 1 | 1 | 1 | 0 | 0 | 0 | 2 | 0 | 0 | 1 | 1 | 0 | 0 | 0 | 1 | 2 | 2 | 2 |

Legend:

0 = Did Not Report, 1 = Partially Reported, 2 = Completely Reported

**Table S5.** Scores for all 98 papers [1-98] for the TIDiER checklist.

| Study ID | BRIEF NAME Score | WHY Score | WHAT 2 Score | WHAT 1 Score | WHO PROVIDED Score | HOW Score | WHERE Score | WHEN and HOW MUCH Score | TAILORING Score | MODIFICATIONS Score | HOW WELL1 Score | HOW WELL2 Score |
| --- | --- | --- | --- | --- | --- | --- | --- | --- | --- | --- | --- | --- |
| 1 | 2 | 2 | 0 | 1 | 1 | 2 | 2 | 1 | 2 | 2 | 1 | 0 |
| 2 | 2 | 2 | 2 | 1 | 1 | 2 | 2 | 2 | 2 | 0 | 1 | 1 |
| 3 | 0 | 2 | 2 | 2 | 0 | 2 | 1 | 1 | 0 | 0 | 0 | 1 |
| 4 | 2 | 2 | 2 | 1 | 0 | 2 | 2 | 0 | 1 | 1 | 1 | 1 |
| 5 | 0 | 2 | 2 | 1 | 0 | 2 | 2 | 1 | 0 | 0 | 1 | 1 |
| 6 | 0 | 2 | 2 | 1 | 1 | 1 | 1 | 1 | 1 | 0 | 1 | 2 |
| 7 | 0 | 2 | 2 | 0 | 0 | 2 | 1 | 1 | 1 | 2 | 1 | 2 |
| 8 | 0 | 1 | 2 | 1 | 0 | 2 | 1 | 0 | 0 | 0 | 0 | 0 |
| 9 | 0 | 2 | 2 | 1 | 0 | 2 | 1 | 1 | 1 | 0 | 1 | 0 |
| 10 | 0 | 2 | 2 | 1 | 0 | 1 | 1 | 1 | 0 | 1 | 1 | 1 |
| 11 | 0 | 2 | 2 | 1 | 1 | 2 | 2 | 1 | 0 | 0 | 1 | 2 |
| 12 | 0 | 2 | 2 | 2 | 0 | 2 | 1 | 1 | 1 | 0 | 0 | 1 |
| 13 | 0 | 1 | 2 | 1 | 0 | 2 | 1 | 0 | 0 | 0 | 0 | 0 |
| 14 | 0 | 2 | 1 | 1 | 0 | 1 | 1 | 1 | 1 | 2 | 0 | 0 |
| 15 | 0 | 1 | 2 | 1 | 1 | 2 | 1 | 0 | 0 | 0 | 0 | 1 |
| 16 | 2 | 1 | 2 | 1 | 1 | 2 | 1 | 1 | 0 | 1 | 2 | 1 |
| 17 | 2 | 2 | 2 | 1 | 1 | 2 | 1 | 1 | 2 | 2 | 2 | 2 |
| 18 | 2 | 2 | 2 | 2 | 1 | 2 | 2 | 1 | 1 | 1 | 1 | 1 |
| 19 | 0 | 2 | 2 | 1 | 1 | 2 | 2 | 1 | 2 | 0 | 1 | 1 |
| 20 | 0 | 2 | 2 | 1 | 0 | 2 | 2 | 2 | 1 | 1 | 1 | 2 |
| 21 | 2 | 2 | 2 | 1 | 0 | 2 | 2 | 2 | 1 | 0 | 1 | 1 |
| 22 | 0 | 2 | 1 | 1 | 0 | 2 | 2 | 1 | 1 | 2 | 1 | 1 |
| 23 | 0 | 2 | 2 | 1 | 1 | 2 | 1 | 2 | 1 | 0 | 1 | 1 |
| 24 | 0 | 2 | 2 | 1 | 1 | 2 | 2 | 1 | 1 | 2 | 2 | 1 |
| 25 | 0 | 1 | 2 | 1 | 0 | 2 | 2 | 1 | 1 | 1 | 1 | 2 |
| 26 | 2 | 2 | 2 | 1 | 1 | 2 | 2 | 2 | 2 | 1 | 1 | 2 |
| 27 | 2 | 2 | 2 | 2 | 1 | 2 | 2 | 1 | 1 | 0 | 1 | 1 |
| 28 | 0 | 1 | 2 | 1 | 1 | 1 | 1 | 1 | 0 | 0 | 0 | 1 |
| 29 | 0 | 1 | 2 | 1 | 1 | 1 | 1 | 1 | 1 | 1 | 1 | 1 |
| 30 | 0 | 2 | 2 | 1 | 0 | 2 | 2 | 2 | 1 | 0 | 2 | 2 |
| 31 | 0 | 2 | 2 | 1 | 0 | 1 | 1 | 1 | 0 | 2 | 1 | 1 |
| 32 | 2 | 2 | 2 | 2 | 1 | 1 | 2 | 1 | 1 | 1 | 2 | 1 |
| 33 | 0 | 2 | 2 | 1 | 1 | 1 | 2 | 1 | 0 | 0 | 1 | 1 |
| 34 | 0 | 2 | 2 | 1 | 2 | 2 | 2 | 1 | 1 | 1 | 1 | 1 |
| 35 | 2 | 2 | 2 | 1 | 0 | 2 | 2 | 2 | 2 | 2 | 2 | 2 |
| 36 | 0 | 2 | 2 | 1 | 1 | 1 | 1 | 1 | 2 | 0 | 1 | 1 |
| 37 | 2 | 2 | 2 | 1 | 1 | 1 | 2 | 0 | 2 | 0 | 1 | 0 |
| 38 | 0 | 1 | 2 | 1 | 1 | 2 | 2 | 1 | 1 | 2 | 1 | 1 |
| 39 | 0 | 2 | 2 | 1 | 0 | 2 | 2 | 1 | 1 | 2 | 1 | 1 |
| 40 | 0 | 2 | 2 | 1 | 1 | 2 | 2 | 1 | 2 | 2 | 1 | 1 |
| 41 | 0 | 2 | 2 | 1 | 1 | 2 | 2 | 1 | 1 | 0 | 1 | 2 |
| 42 | 0 | 2 | 1 | 1 | 1 | 2 | 2 | 1 | 1 | 1 | 1 | 1 |
| 43 | 0 | 2 | 2 | 1 | 1 | 2 | 2 | 1 | 2 | 1 | 2 | 1 |
| 44 | 0 | 2 | 1 | 2 | 1 | 2 | 2 | 1 | 1 | 1 | 1 | 1 |
| 45 | 0 | 2 | 2 | 1 | 0 | 2 | 2 | 2 | 1 | 0 | 1 | 2 |
| 46 | 0 | 2 | 2 | 1 | 1 | 2 | 2 | 1 | 2 | 0 | 1 | 1 |
| 47 | 2 | 2 | 2 | 1 | 0 | 2 | 2 | 1 | 2 | 0 | 1 | 1 |
| 48 | 2 | 2 | 2 | 1 | 1 | 2 | 1 | 2 | 0 | 0 | 2 | 2 |
| 49 | 2 | 2 | 2 | 1 | 1 | 2 | 2 | 1 | 1 | 1 | 2 | 1 |
| 50 | 0 | 1 | 0 | 1 | 1 | 2 | 2 | 0 | 1 | 0 | 0 | 0 |
| 51 | 2 | 2 | 2 | 1 | 1 | 2 | 1 | 1 | 1 | 0 | 1 | 1 |
| 52 | 0 | 2 | 2 | 1 | 1 | 2 | 1 | 1 | 1 | 0 | 1 | 1 |
| 53 | 0 | 2 | 2 | 1 | 0 | 2 | 2 | 1 | 1 | 0 | 1 | 1 |
| 54 | 0 | 2 | 0 | 0 | 1 | 2 | 1 | 2 | 1 | 0 | 1 | 1 |
| 55 | 0 | 2 | 0 | 1 | 0 | 2 | 1 | 1 | 0 | 1 | 0 | 0 |
| 56 | 2 | 2 | 2 | 1 | 0 | 2 | 2 | 2 | 1 | 2 | 2 | 1 |
| 57 | 0 | 2 | 2 | 1 | 1 | 2 | 1 | 1 | 1 | 1 | 1 | 1 |
| 58 | 2 | 2 | 2 | 0 | 1 | 2 | 2 | 1 | 2 | 2 | 2 | 1 |
| 59 | 0 | 1 | 1 | 1 | 1 | 2 | 2 | 1 | 0 | 2 | 0 | 1 |
| 60 | 0 | 2 | 2 | 1 | 0 | 2 | 0 | 0 | 0 | 0 | 0 | 1 |
| 61 | 0 | 2 | 2 | 1 | 1 | 2 | 2 | 1 | 1 | 1 | 1 | 1 |
| 62 | 0 | 2 | 2 | 2 | 0 | 2 | 2 | 2 | 1 | 1 | 1 | 1 |
| 63 | 0 | 2 | 2 | 1 | 1 | 2 | 1 | 1 | 1 | 1 | 1 | 1 |
| 64 | 0 | 2 | 2 | 1 | 0 | 2 | 2 | 1 | 0 | 0 | 1 | 0 |
| 65 | 0 | 2 | 2 | 1 | 1 | 2 | 2 | 2 | 1 | 0 | 1 | 1 |
| 66 | 0 | 2 | 2 | 1 | 1 | 2 | 1 | 1 | 1 | 1 | 1 | 1 |
| 67 | 0 | 1 | 2 | 1 | 0 | 2 | 1 | 1 | 1 | 2 | 1 | 1 |
| 68 | 0 | 2 | 0 | 1 | 0 | 2 | 1 | 1 | 1 | 0 | 1 | 1 |
| 69 | 0 | 1 | 2 | 1 | 0 | 1 | 2 | 1 | 0 | 0 | 0 | 1 |
| 70 | 0 | 2 | 2 | 2 | 1 | 2 | 2 | 2 | 2 | 2 | 1 | 1 |
| 71 | 0 | 2 | 2 | 1 | 1 | 2 | 1 | 1 | 1 | 0 | 2 | 2 |
| 72 | 0 | 0 | 2 | 1 | 0 | 1 | 2 | 0 | 0 | 0 | 0 | 0 |
| 73 | 2 | 2 | 2 | 1 | 0 | 2 | 2 | 1 | 1 | 0 | 1 | 1 |
| 74 | 2 | 2 | 2 | 1 | 0 | 2 | 1 | 1 | 1 | 0 | 0 | 1 |
| 75 | 0 | 2 | 2 | 2 | 1 | 2 | 2 | 1 | 1 | 2 | 1 | 1 |
| 76 | 0 | 1 | 2 | 1 | 0 | 2 | 2 | 0 | 1 | 2 | 0 | 1 |
| 77 | 0 | 2 | 2 | 1 | 1 | 2 | 1 | 1 | 1 | 2 | 1 | 2 |
| 78 | 0 | 0 | 0 | 1 | 0 | 2 | 2 | 0 | 0 | 0 | 0 | 1 |
| 79 | 0 | 2 | 1 | 2 | 1 | 2 | 2 | 2 | 2 | 1 | 2 | 2 |
| 80 | 2 | 2 | 2 | 1 | 1 | 2 | 1 | 2 | 2 | 1 | 1 | 2 |
| 81 | 2 | 2 | 2 | 1 | 1 | 2 | 2 | 1 | 2 | 1 | 1 | 1 |
| 82 | 2 | 2 | 2 | 2 | 1 | 2 | 2 | 2 | 2 | 2 | 1 | 2 |
| 83 | 2 | 2 | 2 | 1 | 2 | 2 | 2 | 2 | 2 | 2 | 2 | 2 |
| 84 | 2 | 2 | 2 | 2 | 1 | 2 | 2 | 1 | 2 | 1 | 0 | 0 |
| 85 | 2 | 2 | 2 | 2 | 1 | 2 | 2 | 2 | 2 | 1 | 1 | 2 |
| 86 | 0 | 2 | 2 | 1 | 1 | 1 | 1 | 0 | 0 | 0 | 0 | 0 |
| 87 | 2 | 2 | 2 | 1 | 0 | 2 | 2 | 2 | 2 | 1 | 1 | 2 |
| 88 | 2 | 2 | 2 | 1 | 0 | 2 | 1 | 1 | 1 | 0 | 0 | 0 |
| 89 | 2 | 2 | 2 | 2 | 2 | 2 | 2 | 2 | 2 | 2 | 2 | 1 |
| 90 | 2 | 0 | 2 | 1 | 2 | 2 | 2 | 1 | 1 | 0 | 1 | 1 |
| 91 | 0 | 2 | 2 | 2 | 0 | 2 | 2 | 1 | 0 | 0 | 1 | 1 |
| 92 | 2 | 2 | 2 | 2 | 2 | 2 | 2 | 2 | 0 | 2 | 2 | 2 |
| 93 | 0 | 2 | 2 | 2 | 1 | 2 | 2 | 1 | 2 | 1 | 1 | 0 |
| 94 | 2 | 1 | 0 | 0 | 1 | 2 | 1 | 1 | 1 | 0 | 0 | 0 |
| 95 | 2 | 2 | 2 | 1 | 1 | 1 | 1 | 1 | 2 | 1 | 1 | 1 |
| 96 | 2 | 2 | 2 | 2 | 1 | 2 | 1 | 2 | 2 | 1 | 1 | 1 |
| 97 | 0 | 2 | 0 | 1 | 1 | 2 | 1 | 0 | 1 | 0 | 0 | 0 |
| 98 | 2 | 2 | 2 | 2 | 1 | 1 | 2 | 1 | 2 | 0 | 0 | 0 |

Legend:

0 = Did Not Report, 1 = Partially Reported, 2 = Completely Reported

**Table S6.** Summary of scores for the iCHECK and TIDiER checklists.

| **Checklist** | | | | | | **Percentage of papers (number) that:** | | | | |
| --- | --- | --- | --- | --- | --- | --- | --- | --- | --- | --- |
|  | | | | | |  | | | | |
|  | | | **Overall adherence to each category (total score), n (%)** | | | **Reported completely, n (%)** | | **Partially reported, n (%)** | | **Did not report, n (%)** |
| **iCHECK-DH** | | | | | | | | | | |
| **Title** | **Title** | | 147 (75.0) | | | 58 (59.1) | | 31 (31.6) | | 9 (9.2) |
| **Abstract** | **Abstract** | | 180 (91.8) | | | 85 (86.7) | | 10 (10.2) | | 3 (3.0) |
| **Introduction** | **Context** | | 103 (52.5) | | | 5 (5.1) | | 93 (94.9) | | 0 |
|  | **Problem statement** | | 179 (91.3) | | | 81 (82.6) | | 17 (17.3) | | 0 |
|  | **Similar interventions** | | 98 (50.0) | | | 11 (11.2) | | 76 (77.5) | | 11 (11.2) |
| **Methods** | **Aims and objectives** | | 134 (68.4) | | | 46 (46.9) | | 42 (42.8) | | 10 (10.2) |
|  | **Blueprint summary** | | 125 (63.7) | | | 46 (46.9) | | 37 (37.7) | | 17 (17.3) |
|  | **Technical design** | | 62 (31.6) | | | 2 (2.0) | | 58 (59.1) | | 38 (38.7 |
|  | **Target** | | 187 (95.4) | | | 89 (90.8) | | 9 (9.2) | | 0 |
|  | **Data** | | 58 (29.6) | | | 0 | | 58 (59.2) | | 40 (40.8) |
|  | **Interoperability** | | 15 (7.6) | | | 0 | | 15 (15.3) | | 83 (84.7) |
|  | **Participating entities** | | 76 (38.7) | | | 1 (1.0) | | 74 (75.5) | | 23 (23.4) |
|  | **Budget planning** | | 12 (6.1) | | | 0 | | 12 (12.2) | | 86 (87.8) |
|  | **Sustainability** | | 44 (22.4) | | | 0 | | 44 (44.9) | | 54 (55.1) |
| **Results** | **Coverage** | | 87 (44.3) | | | 10 (10.2) | | 67 (68.3) | | 21 (21.4) |
|  | **Outcomes** | | 162 (82.7) | | | 75 (76.5) | | 12 (12.2) | | 11 (11.2) |
|  | **Lessons learned** | | 96 (48.9) | | | 1 (1.0) | | 94 (95.9) | | 3 (3.0) |
|  | **Unintended consequences*** | | 100 (51.0) | | | 20 (20.4) | | 60 (61.2) | | 18 (18.3) |
| **Discussion** | **Conclusion** | | 165 (84.1) | | | 79 (80.6) | | 7 (7.1) | | 12 (12.2) |
| **General** | **General*** | | 118 (60.2) | | | 32 (32.7) | | 54 (55.1) | | 12 (12.2) |
| **TIDiER-Telehealth** | | | | | | | | | | |
| **Brief name** |  | | 70 (35.7) | | | 35 (35.7) | | 0 | | 63 (64.2) |
| **Why** |  | | 176 (89.8) | | | 81 (82.7) | | 14 (14.3) | | 3 (3.1) |
| **What** | **What (materials)** | | 113 (57.7) | | | 19 (19.4) | | 75 (76.5) | | 4 (4.1) |
|  | **What (procedures)** | | 174 (88.8) | | | 84 (85.7) | | 6 (6.1) | | 8 (8.2) |
| **Who provided** |  | | 66 (33.7) | | | 5 (5.1) | | 56 (57.1) | | 37 (37.8) |
| **How** |  | | 181 (92.3) | | | 83 (84.7) | | 15 (15.3) | | 0 |
| **Where** |  | | 157 (80.1) | | | 60 (61.2) | | 37 (37.8) | | 1 (1.0) |
| **When and how much** |  | | 109 (55.6) | | | 23 (23.5) | | 63 (64.2) | | 12 (12.2) |
| **Tailoring*** |  | | 103 (52.5) | | | 27 (27.5) | | 49 (50.0) | | 22 (22.4) |
| **Modifications*** |  | | 75 (38.3) | | | 23 (23.5) | | 29 (29.6) | | 46 (46.9) |
| **How well*** | **How (planned)*** | | 92 (46.9) | | | 16 (16.3) | | 60 (61.2) | | 22 (22.4) |
|  | **How (actual)*** | | 102 (52.0) | | | 16 (16.3) | | 54 (55.1) | | 10 (10.2) |
| 100% | | 80%-99% | | 60%-80% | 40%-60% | | 20%-40% | | 0%-20% | |
| * These categories are not mandatory within the original checklist. | | | | | | | | | | |

**Figure S1.** iCHECK (A) frequency of the 3 disagreement types recorded per paper for 18 studies and (B) frequency of the 3 disagreement types recorded per checklist category for the studies.

| **(A)**  **(B)** |  |  |
| --- | --- | --- |
|  |  |  |

**Figure S2.** TIDiER (A) frequency of the 3 disagreement types recorded per paper for the 18 studies and (B) frequency of the 3 disagreement types recorded per checklist category for the 18 studies.

**(A)**

**(B)**

**Figure S3.** Publication frequency based on years.


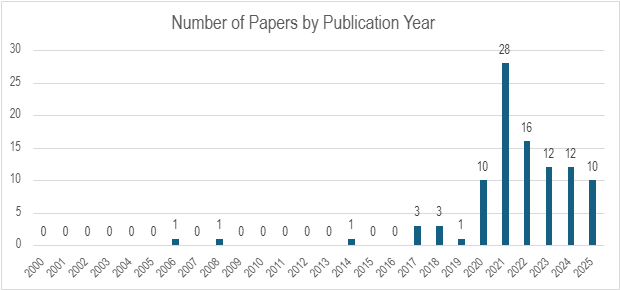


**Figure S4.** Discipline focus of journals (medical and digital health).


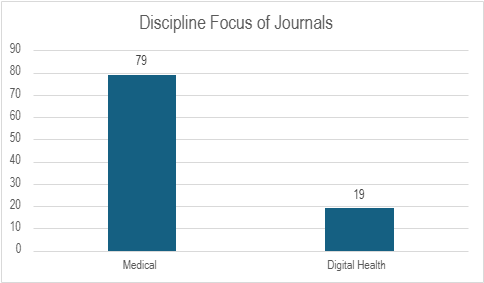


**Figure S5.** Number of papers that self-described as implementation or intervention in the title.
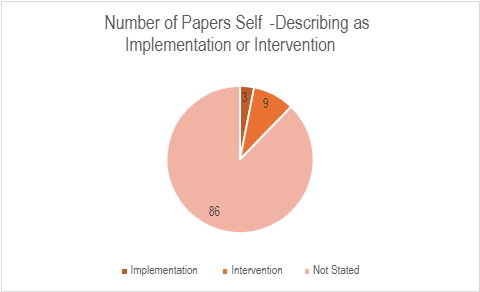


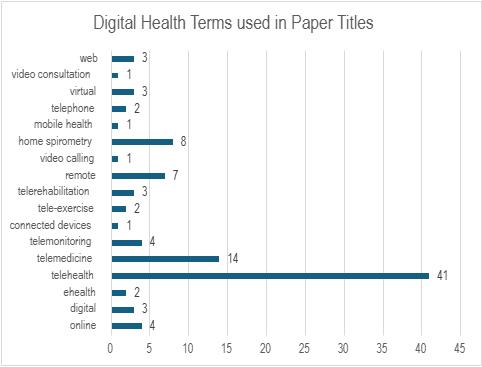
**Figure S6.** Digital health terms used in paper titles.

**Figure S7.** Overall category scores (%) of (A) iCHECK and (B) TIDiER.


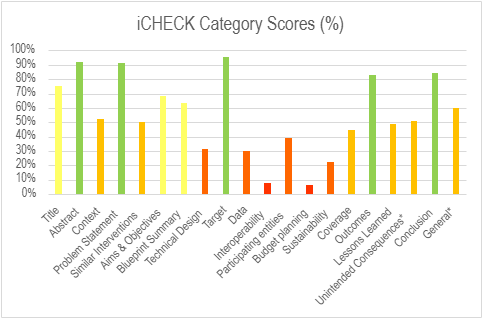
**(A)**


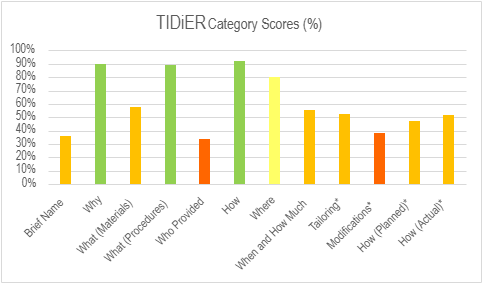
**(B)**

## References

1. Verkleij M, Georgiopoulos AM, Friedman D. Development and evaluation of an internet-based cognitive behavioral therapy intervention for anxiety and depression in adults with cystic fibrosis (eHealth CF-CBT): an international collaboration. *Internet Interv* 2021 Apr; 24:100372.
2. Graziano S, Boldrini F, Righelli D, Milo F, Lucidi V, Quittner A, Tabarini P. Psychological interventions during COVID pandemic: telehealth for individuals with cystic fibrosis and caregivers. *Pediatr Pulmonol* 2021 Jul 27; 56(7):1976-1984.
3. Jaclyn D, Andrew NS, Ryan P, Julianna B, Christopher S, Nauman C, Powers M, Gregory SS, George MS. Patient and family perceptions of telehealth as part of the cystic fibrosis care model during COVID-19. *J Cyst Fibros* 2021 May; 20(3):e23-e28
4. Hasan S, Cecilia Lansang M, Salman Khan M, Dasenbrook E. Managing cystic fibrosis related diabetes via telehealth during COVID-19 pandemic. *J Clin Transl Endocrinol* 2021 Mar; 23:100253.
5. Benz C, Middleton A, Elliott A, Harvey A. Physiotherapy via telehealth for acute respiratory exacerbations in paediatric cystic fibrosis. *J Telemed Telecare* 2021 Mar 16; 29(7):552-560.
6. Franz N, Rapp H, Hansen RN, Gold LS, Goss CH, Lechtzin N, Kessler LG. Health care costs related to home spirometry in the eICE randomized trial. *J Cyst Fibros* 2022 Jan; 21(1):61-69.
7. Hatziagorou E, Toulia I, Avramidou V, Kampouras A, Tsara V, Tsanakas J. Change in CF care during COVID-19 pandemic: single-center experience in a middle-income setting. *Pediatr Pulmonol* 2021 Sept; 56(9):3065-3067.
8. Corcoran J, Marley Campbell C, Ladores S. Transitioning to telehealth during the coronavirus disease 2019 pandemic: perspectives from partners of women with cystic fibrosis and healthcare providers. *Chronic Illn* 2023 Mar; 19(1):95-101.
9. Van Citters AD, Dieni O, Scalia P, Dowd C, Sabadosa KA, Fliege JD, Jain M, Miller RW, Ren CL. Barriers and facilitators to implementing telehealth services during the COVID-19 pandemic: a qualitative analysis of interviews with cystic fibrosis care team members. *J Cyst Fibros* 2021 Dec; 20 Suppl 3:23-28.
10. Ong T, Van Citters AD, Dowd C, Fullmer J, List R, Pai S-A, Ren CL, Scalia P, Solomon GM, Sawicki GS. Remote monitoring in telehealth care delivery across the U.S. cystic fibrosis care network. *J Cyst Fibros* 2021 Dec; 20 Suppl 3:57-63.
11. Beşer ÖF, Karaboğa EPU, Hepkaya E, Kılınç Sakallı AA, Dönmez Türkmen A, Dilek TD, Çokuğraş H, Çullu Çokuğraş F. The role of telehealth services in children with cystic fibrosis during coronavirus disease 2019 outbreak. *Telemed J E Health* 2022 Jun; 28(6):832-837.
12. Hendra K, Neemuchwala F, Chan M, Ly NP, Gibb ER. Patient and provider experience with cystic fibrosis telemedicine clinic. *Front Pediatr* 2021; 9:784692.
13. Solomon GM, Bailey J, Lawlor J, Scalia P, Sawicki GS, Dowd C, Sabadosa KA, Van Citters A. Patient and family experience of telehealth care delivery as part of the CF chronic care model early in the COVID-19 pandemic. *J Cyst Fibros* 2021 Dec; 20 Suppl 3:41-46.
14. Dowd C, Van Citters AD, Dieni O, Willis A, Powell L, Sabadosa KA. Design and methods for understanding the state of cystic fibrosis care amid the COVID-19 pandemic. *J Cyst Fibros* 2021 Dec; 20 Suppl 3:3-8.
15. Ahmed R, Greenfield M, Morley CP, Desimone M. Satisfaction and concerns with telemedicine endocrine care of patients with cystic fibrosis. *Telemed Rep* 2022; 3(1):93-100.
16. Kenis-Coskun Ö, Aksoy AN, Kumaş EN, Yılmaz A, Güven E, Ayaz HH, Sözer T, Ergenekon AP, Karadağ-Saygı E. The effect of telerehabilitation on quality of life, anxiety, and depression in children with cystic fibrosis and caregivers: a single-blind randomized trial. *Pediatr Pulmonol* 2022 May; 57(5):1262-1271.
17. Albon D, Thomas L, Hoberg L, Stamper S, Somerville L, Varghese P, Balasa E, Roman M, Britto MT, Miner M, Gehring E, Gammon C, Amin RS, Seid M, Powers M, CF Learning Network telehealth iLab working group. Cystic fibrosis learning network telehealth innovation lab during the COVID-19 pandemic: a success QI story for interdisciplinary care and agenda setting. *BMJ Open Qual* 2022 May; 11(2):e001844.
18. Rodkjær L, Jeppesen M, Schougaard L. Management of cystic fibrosis during COVID-19: patient reported outcomes based remote follow-up among CF patients in Denmark - a feasibility study. *J Cyst Fibros* 2022 Mar; 21(2):e106-e112.
19. Costa RLF, Costa RF, Gonçalves CP, Cohen RWF, Santana NN. Telemedicine of patients with cystic fibrosis during the COVID-19 pandemic. *Rev Paul Pediatr* 2022; 40:e2021118.
20. Nash EF, Choyce J, Carrolan V, Justice E, Shaw KL, Sitch A, Mistry H, Whitehouse JL. A prospective randomised controlled mixed-methods pilot study of home monitoring in adults with cystic fibrosis. *Ther Adv Respir* Dis 2022; 16:17534666211070133.
21. Bathgate CJ, Kilbourn KM, Murphy NH, Wamboldt FS, Holm KE. Pilot RCT of a telehealth intervention to reduce symptoms of depression and anxiety in adults with cystic fibrosis*. J Cyst Fibros* 2022 Mar; 21(2):332-338.
22. Franciosi AN, Wilcox PG, Quon BS. Cystic fibrosis respiratory microbiology monitoring during a global pandemic: lessons learned from a shift to telehealth. *Ann Am Thorac Soc* 2022 Mar; 19(3):498-500.
23. Fadaizadeh L, Hassanzad M, Valinejadi A, Taheri SMJ, Farnia P, Hassanzad N, Ghaffaripoor HA. Evaluation of mobile phone-based tele-monitoring of cystic fibrosis patients during the COVID-19 pandemic: a 3-year experience in Iran. *Biomed Biotechnol Res J* 2022; 6(2):261-265.
24. Somerville LAL, List RP, Compton MH, Bruschwein HM, Jennings D, Jones MK, Murray RK, Starheim ER, Webb KM, Gettle LS, Albon DP. Real-world outcomes in cystic fibrosis telemedicine clinical care in a time of a global pandemic. *Chest* 2022 May; 161(5):1167-1179.
25. Richardson CH, Orr NJ, Ollosson SL, Irving SJ, Balfour-Lynn IM, Carr SB. Initiating home spirometry for children during the COVID-19 pandemic - a practical guide. *Paediatr Respir Rev* 2022 Jun; 42:43-48.
26. Verkleij M, Georgiopoulos AM, Barendrecht H, Friedman D. Pilot of a therapist-guided digital mental health intervention (eHealth CF-CBT) for adults with cystic fibrosis. *Pediatr Pulmonol* 2023 Jul; 58(7):2094-2103.
27. Watanabe AH, Willis C, Ragsdale R, Biskupiak J, Moore K, Brixner D, Young D. Patient perspectives on the use of digital technology to help manage cystic fibrosis. *Pulm Med* 2023; 2023:5082499.
28. Shanthikumar S, Ruseckaite R, Corda J, Mulrennan S, Ranganathan S, Douglas T. Telehealth use in Australian cystic fibrosis centers: clinician experiences. *Pediatr Pulmonol* 2023 Oct; 58(10):2906-2915
29. Dixon E, Dick K, Ollosson S, Jones D, Mattock H, Bentley S, et al. Telemedicine and cystic fibrosis: Do we still need face-to-face clinics? *Paediatr Respir Rev* 2022; 42: 23-28.
30. Beaufils F, Enaud R, Gallode F, Boucher G, Macey J, Berger P, Fayon M, Bui S. Adherence, reliability, and variability of home spirometry telemonitoring in cystic fibrosis. *Front Pediatr* 2023; 11:1111088.
31. Collaco JM, Albon D, Ostrenga JS, Flume P, Schechter MS, Cromwell EA. Factors associated with receiving CF care and use of telehealth in 2020 among persons with cystic fibrosis in the United States. *J Cyst Fibros* 2023 May; 22(3):456-463.
32. Corinna Morlacchi L, Privitera E, Rossetti V, Santambrogio M, Bellofiore A, Rosso L, Palleschi A, Nosotti M, Blasi F. Telemonitoring: an opportunity in cystic fibrosis lung transplant recipients. *Heliyon* 2023 Oct; 9(10):e19931.
33. Edmondson C, Westrupp N, Short C, Seddon P, Olden C, Wallis C, Brodlie M, Baxter F, McCormick J, MacFarlane S, Brooker R, Connon M, Ghayyda S, Blaikie L, Thursfield R, Brown L, Price A, Fleischer E, Hughes D, Donnelly C, Rosenthal M, Wallenburg J, Brownlee K, Alton EWFW, Bush A, Davies JC. Unsupervised home spirometry is not equivalent to supervised clinic spirometry in children and young people with cystic fibrosis: results from the CLIMB-CF study. *Pediatr Pulmonol* 2023 Oct; 58(10):2871-2880.
34. Stalker HJ, Jonasson AR, Hopfer SM, Collins MS. Improvement in cystic fibrosis newborn screening program outcomes with genetic counseling via telemedicine. *Pediatr Pulmonol* 2023 Dec; 58(12):3478-3486.
35. Layton AM, Irwin AM, Mihalik EC, Fleisch E, Keating CL, DiMango EA, Shah L, Arcasoy SM. Telerehabilitation using fitness application in patients with severe cystic fibrosis awaiting lung transplant: a pilot study. *Int J Telemed Appl* 2021; 2021:6641853.
36. Morsa M, Perrin A, David V, Rault G, Le Roux E, Alberti C, Gagnayre R, Pougheon Bertrand D. Experiences among patients with cystic fibrosis in the mucoexocet study of using connected devices for the management of pulmonary exacerbations: grounded theory qualitative research. *JMIR Form Res* 2024 Jan 23; 8:e38064.
37. Poulsen M, Holland AE, Button B, Jones AW. Preferences and perspectives regarding telehealth exercise interventions for adults with cystic fibrosis: a qualitative study. *Pediatr Pulmonol* 2024 May; 59(5):1217-1226.
38. Rimbaldo K, Frayman KB, Shanthikumar S. The impact of telehealth based care on paediatric cystic fibrosis outcomes. *J Cyst Fibros* 2023 Jul; 22(4):706-709.
39. Bell JM, Dwyer TJ, Cunich M, Dentice RL, Hutchings O, Jo HE, Lau EM, Lee WY, Nolan SA, Munoz P, Raffan F, Shah K, Shaw M, Taylor NA, Visser SK, Yozghatlian VA, Wong KKH, Sivam S. Impact of cystic fibrosis multidisciplinary virtual clinics on patient experience, time commitments and costs. *Intern Med J* 2024 May; 54(5):809-816.
40. Piazza-Waggoner C, Ferguson KS, Daines C, Acton JD, Powers SW. Case study: providing evidence-based behavioral and nutrition treatment to a toddler with cystic fibrosis and multiple food allergies via telehealth. *Pediatr Pulmonol* 2006 Oct; 41(10):1001-1004.
41. Wilkinson OM, Duncan-Skingle F, Pryor JA, Hodson ME. A feasibility study of home telemedicine for patients with cystic fibrosis awaiting transplantation. *J Telemed Telecare* 2008; 14(4):182-185.
42. Murgia F, Corona B, Bianciardi F, Romano P, Tagliente I, Bella S. The application of telemedicine in the follow-up of lung transplantation in a patient with cystic fibrosis. *Clin Ter* 2014; 165(5):e382-e383.
43. Womack C, Farsin R, Farsad M, Chaudary N. Emerging alternatives to conventional clinic visits in the era of COVID-19: adoption of telehealth at VCU adult cystic fibrosis center. *Int J Gen Med* 2020 Nov; 13:1175-1186.
44. Ronan P, Mian A, Carr SB, Madge SL, Lorenc A, Robinson N. Learning to breathe with Tai Chi online - qualitative data from a randomized controlled feasibility study of patients with cystic fibrosis. *Eur J Integr Med* 2020 Dec; 40:101229.
45. Chen JJ, Cooper DM, Haddad F, Sladkey A, Nussbaum E, Radom-Aizik S. Tele-exercise as a promising tool to promote exercise in children with cystic fibrosis. *Front Public Health* 2018; 6:269.
46. Wood J, Mulrennan S, Hill K, Cecins N, Morey S, Jenkins S. Telehealth clinics increase access to care for adults with cystic fibrosis living in rural and remote Western Australia. *J Telemed Telecare* 2016 Jul 20; 23(7):673-679.
47. Tomlinson O, Shelley J, Trott J, Bowhay B, Chauhan R, Sheldon C. The feasibility of online video calling to engage patients with cystic fibrosis in exercise training. *J Telemed Telecare* 2019 Feb 25; 26(6):356-364.
48. Shakkottai A, Kaciroti N, Kasmikha L, Nasr SZ. Impact of home spirometry on medication adherence among adolescents with cystic fibrosis. *Pediatr Pulmonol* 2018 Apr; 53(4):431-436.
49. Gur M, Nir V, Teleshov A, Bar-Yoseph R, Manor E, Diab G, Bentur L. The use of telehealth (text messaging and video communications) in patients with cystic fibrosis: a pilot study. *J Telemed Telecare* 2016 May 13; 23(4):489-493.
50. Sturt J, Dliwayo TR, Forjaz V, Hamilton K, Bryce C, Fraser J, Griffiths F. Eliciting the impact of digital consulting for young people living with long-term conditions (LYNC study): cognitive interviews to assess the face and content validity of two patient-reported outcome measures. *J Med Internet Res* 2018 Oct 11; 20(10):e268.
51. Chrysochoou E-A, Hatziagorou E, Kirvassilis F, Tsanakas J. Telephone monitoring and home visits significantly improved the quality of life, treatment adherence and lung function in children with cystic fibrosis. *Acta Paediatr* 2017 Nov; 106(11):1882.
52. Warda N, Rotolo SM. Virtual medication tours with a pharmacist as part of a cystic fibrosis telehealth visit. *J Am Pharm Assoc* 2021; 61(5):e119-e125.
53. De Biase RV, Cristiani L, Paglia C, Alghisi F, Giordani B, Lucidi V, Bella S. Clinical and microbiological monitoring of cystic fibrosis patients, three years of follow-up via tele-medicine: an empirical research. *Clin Ter* 2020; 171(5):e381-e384.
54. Perkins RC, Davis J, NeSmith A, Bailey J, Powers MR, Chaudary N, Siracusa C, Uluer A, Solomon GM, Sawicki GS. Favorable clinician acceptability of telehealth as part of the cystic fibrosis care model during the COVID-19 pandemic. *Ann Am Thorac Soc* 2021 Sept; 18(9):1588-1592.
55. Compton M, Soper M, Reilly B, Gettle L, List R, Bailey M, Bruschwein H, Somerville L, Albon D. A feasibility study of urgent implementation of cystic fibrosis multidisciplinary telemedicine clinic in the face of COVID-19 pandemic: single-center experience telemedicine. *Telemed J E Health* 2020 Aug; 26(8):978-984.
56. Arden MA, Hutchings M, Whelan P, Drabble SJ, Beever D, Bradley JM, Hind D, Ainsworth J, Maguire C, Cantrill H, O'Cathain A, Wildman M. Development of an intervention to increase adherence to nebuliser treatment in adults with cystic fibrosis: CFHealthHub. *Pilot Feasibility Stud* 2021 Jan 04; 7(1):1.
57. Nobili RM, Gambazza S, Spada MS, Tutino AL, Bulfamante AM, Mariani A, Brivio A, Moioli L, Rizzato E, Sansotta N, Claut L, Faelli N, Norsa L, Colombo C. Remote support by multidisciplinary teams: a crucial means to cope with the psychological impact of the SARS-COV-2 pandemic on patients with cystic fibrosis and inflammatory bowel disease in Lombardia. *Int J Clin Pract* 2021 Jul; 75(7):e14220.
58. Albon D, Van Citters AD, Ong T, Dieni O, Dowd C, Willis A, Sabadosa KA, Scalia P, Reno K, Oates GR, Schechter MS. Telehealth use in cystic fibrosis during COVID-19: association with race, ethnicity, and socioeconomic factors. *J Cyst Fibros* 2021 Dec; 20 Suppl 3:49-54.
59. Wickerson L, Helm D, Gottesman C, Rozenberg D, Singer LG, Keshavjee S, Sidhu A. Telerehabilitation for lung transplant candidates and recipients during the COVID-19 pandemic: program evaluation. *JMIR Mhealth Uhealth* 2021 Jun 17; 9(6):e28708.
60. Kayser MZ, Valtin C, Greer M, Karow B, Fuge J, Gottlieb J. Video consultation during the COVID-19 pandemic: a single center's experience with lung transplant recipients. *Telemed J E Health* 2021 Jul; 27(7):807-815.
61. Rad EJ, Mirza AA, Chhatwani L, Purington N, Mohabir PK. Cystic fibrosis telemedicine in the era of COVID-19. *JAMIA Open* 2022 Apr; 5(1):ooac005.
62. Doumit M, Ledwos R, Plush L, Chuang S, Gray M, Jaffe A, McBride J. Telehealth application of an ultrasonic home spirometer. *Arch Dis Child* 2022 Aug; 107(8):752-754.
63. Fettes E, Riley M, Brotherston S, Doughty C, Griffiths B, Laverty A, Aurora P. "You're on mute!" Does pediatric CF home spirometry require physiologist supervision?. *Pediatr Pulmonol* 2022 Jan; 57(1):278-284.
64. Ozsezen B, Emiralioglu N, Tural D, Sunman B, Buyuksahin H, Yalcin E, Dogru D, Ozcelik U, Kiper N. Telephone surveillance during the COVID-19 pandemic: is it a helpful diagnostic tool for detecting acute pulmonary exacerbations in children with chronic lung disease?. *J Telemed Telecare* 2020 Nov 12; 28(9):694-702.
65. Cox NS, Eldridge B, Rawlings S, Dreger J, Corda J, Hauser J, Button BM, Bishop JR, Nichols A, Middleton A, Ward N, Dwyer T, Dentice R, Lazarus R, O'Halloran P, Lee JYT, Mellerick C, Mackintosh K, McNarry M, Williams CA, Holland AE, Youth Activity Unlimited – A Strategic Research Centre of the UK Cystic Fibrosis Trust. Web-based physical activity promotion in young people with CF: a randomised controlled trial. *Thorax* 2023 Jan; 78(1):16-23.
66. Bouteleux B, Beaufils F, Fayon M, Bui S. Home-spirometry exacerbation profiles in children with cystic fibrosis. *Pediatr Pulmonol* 2024 Mar; 59(3):552-561.
67. Yoon E, Hur S, Curtis LM, Benavente JY, Wolf MS, Serper M. Patient factors associated with telehealth quality and experience among adults with chronic conditions. *JAMIA Open* 2024 Jul; 7(2):ooae026.
68. Morrison L, McCrea G, Palmer S. Online activity - a beaming good initiative! Delivering alternative exercise opportunities for people with cystic fibrosis. *Physiother Theory Pract* 2024 Jul; 40(7):1609-1615.
69. Federici A, De Marchis M, Alghisi F, Fiocchi AG, Bella S. Telemonitoring for cystic fibrosis patients of Bambino Gesù Children's Hospital during COVID-19. *Clin Ter* 2022; 173(5):440-442.
70. Kodjebacheva GD, Tang C, Groesbeck F, Walker L, Woodworth J, Schindler-Ruwisch J. Telehealth use in pediatric care during the COVID-19 pandemic: a qualitative study on the perspectives of caregivers. *Children (Basel)* 2023 Feb 06; 10(2):311.
71. O'Hayer CV, O'Loughlin CM, Nurse CN, Smith PJ, Stephen MJ. ACT with CF: a telehealth and in-person feasibility study to address anxiety and depressive symptoms among people with cystic fibrosis. *J Cyst Fibros* 2021 Jan; 20(1):133-139.
72. Oates GR, Mims C, Geurs R, Bergquist R, Hager A, Guimbellot JS, Hartzes AM, Gutierrez HH. Mobile health platform for self-management of pediatric cystic fibrosis: impact on patient-centered care outcomes. *J Cyst Fibros* 2023 Sept; 22(5):823-829.
73. List R, Compton M, Soper M, Bruschwein H, Gettle L, Bailey M, Starheim E, Kalmanek J, Somerville L, Albon D. Preserving multidisciplinary care model and patient safety during reopening of ambulatory cystic fibrosis clinic for nonurgent care: a hybrid telehealth model. *Telemed J E Health* 2021 Feb; 27(2):193-199.
74. Faiçal AVB, Souza EL, Terse-Ramos R. Family perception of a telehealth program for people with cystic fibrosis during the COVID-19 pandemic in northeastern Brazil. *J Bras Pneumol* 2023 Nov 17; 49(5):e20230242.
75. Enochs C, Filbrun AG, Iwanicki C, Moraniec H, Lehrmann J, Stiffler J, Dagher S, Tapley C, Phan H, Raines R, Nasr SZ. Development of an interdisciplinary telehealth care model in a pediatric cystic fibrosis center. *Telemed Rep* 2021; 2(1):224-232.
76. Shanthikumar S, Moore E, Corda J, Reardon N, Louey S, Frayman K, Harrison J, Ranganathan S. Patient and family perspectives regarding the use of telehealth for cystic fibrosis care. *Pediatr Pulmonol* 2021 May; 56(5):811-813.
77. Compton M, List R, Starheim E, Somerville L, Williamson L, Murray R, Jennings D, Bruschwein H, Albon D. Home spirometry utilisation in telemedicine clinic for cystic fibrosis care during COVID-19 pandemic: a quality improvement process. *BMJ Open Qual* 2021 Aug; 10(3):e001529.
78. Horrell LN, Hayes S, Herbert LB, MacTurk K, Lawhon L, Valle CG, Bhowmick A. Telemedicine use and health-related concerns of patients with chronic conditions during COVID-19: survey of members of online health communities. *J Med Internet Res* 2021 Feb 18; 23(2):e23795.
79. Thee S, Stahl M, Fischer R, Sutharsan S, Ballmann M, Müller A, Lorenz D, Urbanski-Rini D, Püschner F, Amelung VE, Fuchs C, Mall MA. A multi-centre, randomized, controlled trial on coaching and telemonitoring in patients with cystic fibrosis: conneCT CF. *BMC Pulm Med* 2021 Apr 21; 21(1):131.
80. Hillen B, Simon P, Schlotter S, Nitsche O, Bähner V, Poplawska K, Pfirrmann D. Feasibility and implementation of a personalized, web-based exercise intervention for people with cystic fibrosis for 1 year. *BMC Sports Sci Med Rehabil* 2021 Aug 19; 13(1):95.
81. Woods BM, Bray LA, Campbell SB, Li P, Kazmerski TM, Hovater C, Pitts LN, Ladores S. Implementation and evaluation of a fertility preservation telehealth counseling intervention for males with cystic fibrosis. *J Cyst Fibros* 2024 Jul; 23(4):658-663.
82. Kilic K, Vardar-Yagli N, Nayir-Buyuksahin H, Guzelkas I, Dogru D, Saglam M, Calik-Kutukcu E, Inal-Ince D, Emiralioglu N, Yalcin E, Ozcelik U, Kiper N. Exercise intolerance, oxidative stress, and irisin in pediatric cystic fibrosis: can telehealth-based exercise training make a difference?. *Heart Lung* 2024; 68:145-153.
83. Bathgate CJ, Smith ED, Murphy NH, Quittner AL, Riekert KA, Goralski JL, Holm KE. Coping and learning to Manage Stress with cystic fibrosis (CALM): a multisite telehealth randomized controlled trial to reduce depression and anxiety symptoms in adults with cystic fibrosis. *J Cyst Fibros* 2025 Mar; 24(2):310-318.
84. Langfelder-Schwind E, Basile M, Moyal-Smith R, Polo J, McGinniss MA, Petersen J, Talavera J, Schwind H, Parad RB, Raraigh KS. Increasing access to genetic counselors with disease-specific expertise: development of a centralized cystic fibrosis genetic counseling telehealth model. *Pediatr Pulmonol* 2025 Dec; 60(12):e71416.
85. Ozyemisci Taskiran O, Albayrak H, Kog C, Atli E, Gonullu E, Yantac AE, Uyan ZS. The effect of a 12-week tele-exercise using immersive virtual reality on functional capacity in adolescents with cystic fibrosis: a randomized controlled, single (assessor) - blind study. *Respir Med* 2025 Nov; 248:108362.
86. Davis J, Gibson SL, Perkins RC, Greenberg J, Simoneau T, Sawicki GS. Perceptions of telehealth in pediatric pulmonary clinic post-pandemic. *Pediatr Pulmonol* 2025 Aug; 60(8):e71237.
87. Medbo J, Imberg H, Hansen C, Krantz C, de Monestrol I, Svedberg M. Telemedicine and home spirometry in cystic fibrosis: a prospective multicenter study. *Pediatr Pulmonol* 2024 Nov; 59(11):2967-2975.
88. Davis J, Perkins R, Bailey J, Chaudary N, Garcia B, Froh D, Powers M, Siracusa C, Sawicki GS. Acceptability of telehealth post-pandemic among clinicians across the United States caring for people with cystic fibrosis. *Pediatr Pulmonol* 2025 Feb; 60(2):e70000.
89. Schechter MS, Molzhon A, Everhart RS, Kang L, Weiskittle R, Castleberry B, Thompson NJ. Impact of UPLIFT, a group telehealth intervention, on symptoms of depression and anxiety in adults with CF. *J Cyst Fibros* 2025 Mar; 24(2):319-325.
90. Cox NS, Dal Corso S, Burge AT, Bondarenko J, Perryman J, Holland AE. Remote assessment of exercise capacity in adults with chronic respiratory disease: safety, reliability and acceptability. *Chron Respir Dis* 2025; 22:14799731251318033.
91. Vendrusculo FM, da Costa GA, Bagatini MA, Lemes BMHM, Faria CA, de Oliveira LC, Aquino ES, Donadio MVF. Feasibility of performing the 3-minute step test with remote supervision in children and adolescents with cystic fibrosis: a comparative study. *Pediatr Investig* 2024 Jun; 8(2):83-90.
92. O'Hayer CV, Smith PJ, Drescher CF, Bruschwein H, Nurse CN, Kushner HM, Ingle K, Stephen MJ, Hoag JB. ACT with CF: a randomized trial of acceptance and commitment therapy vs supportive psychotherapy for adults with cystic fibrosis. *Gen Hosp Psychiatry* 2024; 91:212-222.
93. Doumit M, Pacey V, Jaffe A, Gray K. Understanding the acceptability of the changing model of care in cystic fibrosis. *Respir Med* 2024; 234:107847.
94. Albon D, Ong T, Horton B, Brighton D, Shen S, List R, Antos N, Asfour F, Balasa E, Beachler D, Daines C, Froh D, Kier C, Nasr S, Sathe M, Sawicki G, Schechter M, Solomon G, Powers M. Cystic fibrosis learning network telehealth innovation lab during the COVID-19 pandemic: impact on access to care, outcomes, and a new CF care model. *Pediatr Pulmonol* 2025 May; 60(5):e71102.
95. Reis LMS, Pinheiro AAC, Silva Júnior MA, Gonçalves CP, Santana NN. Impact of telehealth during the COVID-19 pandemic on clinical and nutritional conditions of adolescents with cystic fibrosis. *J Bras Pneumol* 2024 May 27; 50(2):e20230397.
96. De Marchis M, Rivolta M, De Dominicis S, Ciarnella M, Leone P, Milo F, Di Giovanni D, D’amore C, Federici A, Boni A, Alghisi F, Bella S, Cannataro M, Cutrera R. WEB-REHAB Program for people with cystic fibrosis during COVID-19 pandemic: a feasibility study. *Minerva Respir Med* 2025 Mar; 64(1):1-8.
97. Douglas TA, Pourghaderi AR, Ahern S, Corda J, Earnest A, Mulrennan S, Ranganathan S, Ruseckaite R, Shanthikumar S. The impact of telehealth on clinical outcomes in adults and children with cystic fibrosis in Australia*. J Cyst Fibros* 2026 Mar; 25(2):369-374.
98. Qin Y, Hamana K, Gale N. Remote exercise services for people with cystic fibrosis: experiences and perceptions from people with cystic fibrosis and members of cystic fibrosis multidisciplinary teams. *Disabil Rehabil* 2025 Jun; 47(13):3353-3366.
